# The Caffeinated Brain Part 1: The Effects of Caffeine on Event-Related Potentials (ERPs)—A Systematic and Mechanistic Review

**DOI:** 10.3390/nu18101489

**Published:** 2026-05-07

**Authors:** James Chmiel, Agnieszka Malinowska, Donata Kurpas

**Affiliations:** 1 Institute of Physical Culture Sciences, Faculty of Physical Culture and Health, University of Szczecin, Al. Piastów 40B Block 6, 71-065 Szczecin, Poland; 2 Institute of Psychology, University of Szczecin, 71-017 Szczecin, Poland; 3 Division of Research Methodology, Department of Nursing, Faculty of Nursing and Midwifery, Wroclaw Medical University, 51-618 Wroclaw, Poland

**Keywords:** caffeine, event-related potentials, ERP, neurophysiology, nutrition

## Abstract

Introduction: Caffeine is a widely consumed adenosine receptor antagonist with well-documented effects on arousal and performance, but its time-resolved neurophysiological signature across stages of information processing remains fragmented across event-related potential (ERP) paradigms. Objectives: This systematic and mechanistic review aimed to (i) identify and catalog human ERP studies testing caffeine effects, (ii) synthesize findings by task domain and ERP component family, and (iii) evaluate moderators including dose, timing, abstinence/withdrawal control, sleep status, and habitual use. Methods: Following PRISMA 2020 and PRISMA-S, we searched multiple databases (PubMed/MEDLINE, Embase, APA PsycINFO, Web of Science Core Collection, Scopus, IEEE Xplore, and Cochrane Central Register of Controlled Trials) from inception to 28 November 2025 and conducted a structured narrative synthesis using SWiM (Synthesis Without Meta-analysis, no prespecified quantitative pooling). Risk of bias was assessed using RoB-2 (Risk of Bias 2, including crossover extension) and ROBINS-I (Risk Of Bias In Nonrandomized Studies of Interventions). Of 761 records, 63 controlled human studies met the inclusion criteria. The evidence most consistently supported stage- and context-dependent modulation. Within the P3 family, target-related P3b/P300 latency was frequently shortened, or fatigue-related slowing was prevented, often without parallel increases in amplitude. P300 amplitude findings were mixed and context-dependent: amplitude was often unchanged in rested or low-demand paradigms, but increased or was restored when caffeine counteracted fatigue, sleep loss, sustained attention demands, or high workload. Preparatory activity (CNV/slow negativity) showed selective effects, while early sensory components were comparatively stable in many paradigms; higher doses (approximately 200–400 mg) were associated with weaker early auditory sensory gating in some studies. Conclusions: Across heterogeneous paradigms, caffeine was associated with context-dependent ERP changes rather than a uniform amplification of ERP amplitudes. The most consistent pattern was shorter or preserved latency of late positive ERP components, particularly in tasks requiring stimulus evaluation or target detection. In some fatigue, sleep deprivation, sustained attention, or high-demand paradigms, caffeine was also associated with larger or restored P300/P3b amplitudes. These findings are compatible with state-dependent changes in attentional engagement or stimulus evaluation, but mechanistic interpretation remains limited by heterogeneity in task paradigms, ERP definitions, dosing, abstinence procedures, and participant caffeine use profiles. Methodological heterogeneity, small samples, inconsistent control of habitual use and withdrawal, and the predominance of healthy young adult samples limit generalizability, particularly to children, older adults, clinical populations, and long-term high-dose caffeine users.

## 1. Introduction

Caffeine (1,3,7-trimethylxanthine) is a naturally occurring methylxanthine found in coffee beans, tea leaves, cacao, kola nut, and many other plants, and it is also added to beverages, supplements, and some medications. Because it is widely consumed and crosses the blood–brain barrier, caffeine has become a model compound for studying how a small, lipid-soluble molecule can influence arousal, mood, and performance in everyday life. Large evidence bases exist across regulatory science, clinical pharmacology, and sleep/exercise research, allowing caffeine’s effects to be characterized from molecular mechanisms through population guidance [1,2].

After acute ingestion, caffeine is rapidly absorbed, readily enters the brain, and primarily acts at typical dietary doses through adenosine A1/A2A receptor antagonism, making it a useful model compound for studying how altered arousal state influences cognition [3].

Pharmacokinetically, caffeine is efficiently absorbed and distributed, with substantial inter-individual variability in clearance. Authoritative pharmacology references report an average adult plasma half-life on the order of hours, but with a wide range driven by physiology and environment (e.g., pregnancy, smoking, oral contraceptives) and by hepatic enzymatic activity. A comprehensive safety review summarizes typical adult half-life estimates (~3–7 h) and emphasizes that neonates clear caffeine far more slowly due to immature metabolic pathways [2,4].

One of caffeine’s most reproducible behavioral effects is the disruption of subsequent sleep, particularly when consumed later in the day or at higher doses [5]. A recent systematic review and meta-analysis quantified reductions in total sleep time and sleep efficiency and increases in sleep onset latency and wake after sleep onset following caffeine intake, consistent with caffeine’s antagonism of adenosine-mediated sleep pressure. Newer work also continues to refine how dose and timing interact, reinforcing that “how late” can matter as much as “how much” [6,7].

Although behavioral studies consistently show that caffeine can alter alertness, reaction time, vigilance, and task performance, behavioral outcomes alone do not reveal which stage of information processing has changed. A faster response, for example, may reflect enhanced sensory registration, more efficient attentional allocation, accelerated stimulus evaluation, improved response preparation, or altered performance monitoring. Event-related potentials (ERPs) are especially well-suited to this problem because they provide millisecond-level markers of partially separable processing stages, allowing caffeine effects to be localized more precisely within the stream from perception to action.

ERPs are voltage fluctuations extracted from the scalp electroencephalogram (EEG) that are time-locked to sensory, cognitive, or motor events. Because single-trial EEG contains substantial ongoing activity unrelated to the event of interest, ERPs are most estimated by averaging many epochs aligned to repeated events, which attenuates non-phase-locked background activity and reveals consistent event-locked structure. This makes ERPs especially useful for testing mechanistic accounts of information processing that make temporal predictions on the order of tens of milliseconds (e.g., early perceptual selection vs. later decision or response stages). In cognitive neuroscience, ERPs are often interpreted as a sequence of partially overlapping “components” (deflections with characteristic timing, scalp distribution, and functional sensitivity) rather than as responses from a single unitary generator [8,9].

Methodologically, ERP measurement is inseparable from signal-processing and reporting choices: referencing, filtering, baseline correction, artifact handling (eye blinks, saccades, muscle activity), and quantification (mean amplitude vs. peak amplitude; a priori windows vs. adaptive windows) can all change effect sizes and interpretability. Community guidance emphasizes transparent reporting of acquisition parameters, preprocessing steps, and statistical choices, because seemingly minor differences (e.g., filter settings or artifact rejection thresholds) can affect both component morphology and the risk of analytic flexibility. Modern best practice also increasingly treats ERP analysis as part of a reproducible workflow: explicit pipelines, shareable code, and standardized data structures reduce ambiguity and facilitate reanalysis across labs [10,11].

Because caffeine antagonizes adenosine receptors and shifts arousal and neuromodulatory tone, it is expected to influence cortical excitability and the allocation of attentional resources. In ERP terms, this could appear as changes in early sensory gain (e.g., P1/N1), mid-latency indices of selective attention and control, and late components related to context updating and decision formation (e.g., P3 subcomponents). Interpreting such effects also requires attention to habitual use and abstinence, because many observed benefits may reflect withdrawal reversal rather than net enhancement.

Historically, one of the most studied “endogenous” components is the P300 (often subdivided into P3a/P3b), a late positivity that is typically larger for task-relevant or infrequent events in oddball paradigms. Contemporary accounts link P3a to attentional orienting and P3b to context updating and decision-related processing, making P3 measures helpful in testing whether caffeine alters task engagement and resource allocation during stimulus evaluation [12,13]. To standardize terminology in this review, we use the term P3 family to refer to late positive ERP activity in the approximate 250–600 ms range, commonly elicited by task-relevant, infrequent, novel, or response-inhibitory events. We use P3b for the task-relevant, target-locked positivity, usually maximal over centro-parietal or parietal sites and commonly interpreted in relation to context updating, stimulus evaluation, and decision-related processing. We use P3a to refer to the more fronto-central positivity elicited by novel, distracting, or No-Go events, and it is commonly linked to attentional orienting or involuntary attention switching. We retain P300 only when the original study used this broader label or did not provide sufficient information to separate P3a from P3b. We use late positive component or late positive activity only as a nonspecific descriptor when the reported effect cannot be confidently assigned to P3a or P3b.

Language research highlights the N400, a centro-parietal negativity peaking around 400 ms, which is sensitive to semantic expectancy and contextual fit. Because the N400 magnitude varies with predictive context and processing demands, it provides a time-resolved marker for assessing whether caffeine shifts the efficiency of semantic access and integration across different alertness states [14,15].

Auditory change detection is often indexed by the mismatch negativity (MMN), a fronto-central negativity elicited when a deviant sound violates a learned regularity. MMN is frequently interpreted as reflecting prediction-error or memory-comparison processes that can be observed with minimal task-providing conditions, meaning a way to test whether caffeine modulates deviance detection and pre-attentive processing under varying arousal levels [16,17].

In vision, the N170 is a negative deflection maximal over occipitotemporal sites at ~170 ms, typically larger for faces than for many nonface objects, and sensitive to inversion. Its early latency helps distinguish effects on perceptual encoding from later evaluation stages, which can be useful when assessing whether caffeine influences early sensory or category-selective processing [18].

Finally, cross-study comparability depends strongly on analytic choices and reporting practices. Community resources and standards (e.g., benchmark paradigms with reference pipelines, structured data organization such as EEG-BIDS, and transparent workflow reporting) support reproducible ERP analysis and more credible synthesis of component-specific effects across laboratories [11,19,20].

Despite extensive behavioral and pharmacological evidence on caffeine, the ERP literature remains mechanistically fragmented. Previous studies have examined caffeine effects across diverse paradigms, doses, abstinence conditions, and participants. Still, the findings have rarely been integrated according to the neurocognitive processing stage that each ERP component is presumed to index. As a result, it remains unclear whether caffeine primarily enhances early sensory registration, attentional selection, stimulus evaluation, response preparation, and performance monitoring, or whether the apparent benefits mainly reflect fatigue or withdrawal reversal. This represents an important knowledge gap: there is currently no systematic synthesis that integrates caffeine-related ERP outcomes into a mechanistic framework linking component-level effects to specific stages of information processing while also accounting for moderators such as dose, timing, habitual use, abstinence, sleep status, and task demands.

The main objective of this systematic and mechanistic review was therefore to determine how caffeine modulates human ERP indices of information processing across task domains and ERP component families. Specifically, this review aimed to: (i) identify and catalog controlled human studies that tested caffeine effects using scalp-recorded ERPs; (ii) synthesize findings by task domain and ERP component family, including early sensory components, mid-latency attention/control components, late positive components, error/feedback-related components, and preparatory slow potentials; and (iii) evaluate whether caffeine effects are moderated by dose, timing, abstinence or withdrawal control, sleep or fatigue status, habitual caffeine use, and methodological choices.

The review was guided by the following research questions: (1) Which ERP components are most consistently affected by caffeine across human experimental studies? (2) Do caffeine-related ERP changes suggest the modulation of early sensory processing, attentional allocation, stimulus evaluation, response preparation, or performance monitoring? (3) Are caffeine effects more evident under specific baseline states, such as fatigue, sleep loss, or abstinence? (4) How do dose, timing of administration, habitual use, and study design influence the direction and consistency of ERP findings? By addressing these questions, the review seeks to clarify whether caffeine has a coherent neurophysiological signature or whether its ERP effects are conditional, task-dependent, and shaped by participant state and methodological variation.

## 2. Materials and Methods

### 2.1. Protocol, Registration, and Reporting Framework

This systematic review was designed and reported in accordance with the PRISMA 2020 statement. Search methods and reporting of database strategies followed PRISMA-S to support reproducibility. Given the expected heterogeneity across ERP paradigms, preprocessing and quantification choices, component definitions (time windows and electrodes/ROIs), and outcome reporting, we prespecified a structured narrative synthesis rather than using quantitative pooling. Synthesis and reporting therefore followed the SWiM (Synthesis Without Meta-analysis) guideline, including prespecification of study groupings, rules for selecting outcomes when multiple measures were reported, and a standardized approach for summarizing the direction (and, where available, the magnitude) of effects. This systematic review was registered on PROSPERO (CRD420261336020).

### 2.2. Eligibility Criteria

Eligibility criteria were defined a priori using a PICO framework.

Population. Human participants of any age (healthy or clinical populations), provided that ERPs were recorded using scalp EEG.

Intervention. Caffeine administration in any form (e.g., capsules/tablets; caffeinated coffee/tea; energy drinks), delivered acutely (single dose) or as repeated dosing. Studies of multi-ingredient products were eligible only if the caffeine effect could be isolated, defined a priori as either: (i) a caffeine-only arm and an otherwise matched placebo/vehicle control, or (ii) a factorial design permitting estimation of a caffeine main effect (or a clearly interpretable caffeine contrast) independent of other active ingredients. Studies in which caffeine was co-administered with other psychoactive substances without a caffeine-isolating contrast (e.g., caffeine + alcohol only, caffeine + theanine only, or complex “energy” blends without separable contrasts) were excluded from the primary synthesis; where such studies were otherwise highly relevant, they were cataloged separately as adjunct evidence and explicitly labeled as “non-isolable caffeine contrasts”.

Comparator. Placebo, decaffeinated control, no-caffeine control, or alternative caffeine dose/timing conditions, including within-subject crossover comparisons.

Outcomes. ERP measures derived from scalp EEG, including component amplitude and/or latency for any time-locked component (e.g., P1/N1, N2, P3/P300, MMN, ERN/Ne, FRN, CNV, LPP, N400, P600), recorded during any task paradigm (e.g., oddball, Go/No-Go, flanker, feedback/reward, language, sensory change detection). Studies whose outcomes were primarily brain–computer interface (BCI) features or classification performance (e.g., EEG-feature-based accuracy) were excluded unless conventional ERP component measures were reported with interpretable amplitude/latency outcomes. Purely time-frequency or non-event-locked EEG outcomes were excluded unless accompanied by conventional ERP measures.

Study designs. Randomized controlled trials (parallel-group or crossover), quasi-experiments, and other controlled experimental designs that included a defined exposure contrast. Purely observational studies without a controlled caffeine exposure contrast were excluded. Animal-only studies were excluded.

Exclusion criteria were defined before screening. Studies were excluded if they: (i) were animal or in vitro studies; (ii) did not include caffeine administration or did not provide an interpretable caffeine-specific exposure contrast; (iii) examined caffeine only as part of a multi-ingredient intervention without a caffeine-only arm, matched comparator, or factorial design allowing isolation of the caffeine effect; (iv) did not report conventional scalp-recorded ERP amplitude or latency outcomes; (v) reported only resting EEG, time-frequency, neuroimaging, behavioral, or BCI classification outcomes without interpretable ERP component measures; (vi) were observational studies without controlled caffeine exposure; (vii) were reviews, editorials, commentaries, protocols, or conference abstracts without sufficient original data; or (viii) represented duplicate or overlapping reports of the same dataset. When overlapping reports were identified, the most complete report was retained, and supplementary information from companion publications was used only when it clarified eligibility or extracted outcomes.

### 2.3. Information Sources

We searched multiple bibliographic databases from 1 January 1980 to 28 November 2025, including MEDLINE (via PubMed and/or Ovid), Embase, APA PsycINFO, Web of Science Core Collection, Scopus, and IEEE Xplore (to capture engineering-oriented EEG/BCI conference proceedings). Where available, we also searched CENTRAL (Cochrane Central Register of Controlled Trials). To reduce publication and selective reporting bias, we searched trial registries (ClinicalTrials.gov and the WHO ICTRP, where available). We screened (i) reference lists of included studies and key reviews and (ii) forward citations of included studies using citation indexing.

### 2.4. Search Strategy

To improve reproducibility, the full search strategy was developed iteratively by combining caffeine-related terms with ERP/EEG-related terms and component-specific terms. The PubMed/MEDLINE search strategy used the following core structure:

(caffeine OR caffein OR methylxanthine OR coffee OR tea OR “energy drink*”) AND (“event-related potential*” OR ERP OR ERPs OR electroencephalograph* OR EEG OR P300 OR P3 OR P3b OR P3a OR N400 OR “mismatch negativity” OR MMN OR “error-related negativity” OR ERN OR Ne OR “feedback-related negativity” OR FRN OR “contingent negative variation” OR CNV OR “late positive potential” OR LPP OR P600)**.

This strategy was adapted for each database using database-specific subject headings and syntax, including MeSH terms in MEDLINE and Emtree terms in Embase where applicable. Boolean operators, truncation, phrase searching, and proximity operators were adjusted according to the requirements of each interface. No restrictions were applied for publication year, and searches covered the period from database inception or 1 January 1980, depending on database coverage, to 28 November 2025. Searches were supplemented by backward citation searching of included studies and relevant reviews and by forward citation tracking of included studies. Trial registries were searched to identify unpublished or ongoing studies.

### 2.5. Study Selection

Records were imported into EndNote and deduplicated using automated matching, followed by manual verification of author, year, title, journal, and DOI details as needed. Screening was conducted in two sequential stages: (1) title/abstract screening and (2) full-text review. In both stages, two reviewers independently assessed each record against the prespecified eligibility criteria.

During title/abstract screening, records were retained for full-text review whenever eligibility could not be confidently excluded from the abstract alone. Abstracts were excluded only when at least one exclusion criterion was clearly met, namely: non-human study; no caffeine exposure or no relevant caffeine comparison; no ERP outcome derived from scalp EEG; non-original report (e.g., review, editorial, protocol); or clearly uncontrolled/observational design without an interpretable caffeine exposure contrast.

During full-text review, the same two reviewers independently evaluated each article using a standardized decision form based on the PICO criteria. For reproducibility, one primary reason for exclusion was assigned to each excluded full-text report. Primary exclusion categories were: (i) no eligible caffeine-isolating contrast; (ii) no conventional ERP outcome derived from scalp EEG amplitude/latency measures; (iii) non-eligible study design; (iv) non-human study; (v) duplicate/overlapping report of the same dataset; or (vi) insufficient information to confirm eligibility. Studies involving multi-ingredient products were included only when the caffeine effect could be isolated a priori, either through a caffeine-only arm with a matched comparator or through a factorial design permitting estimation of an interpretable caffeine-specific contrast. Studies focused primarily on BCI classification/features, time-frequency outcomes without conventional ERP measures, or interventions in which caffeine effects could not be separated from other active manipulations were excluded from the primary synthesis.

**Figure 1:**
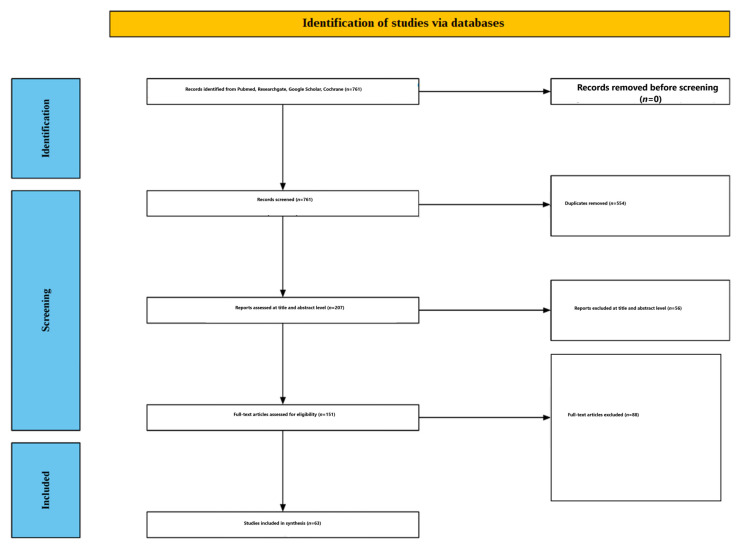
Flow chart depicting the different phases of the systematic review.

Disagreements at either screening stage were resolved by discussion; if consensus could not be reached, a third reviewer adjudicated. Reasons for exclusion at the full-text stage were documented and are summarized in Figure 1.

### 2.6. Data Extraction and Data Items

A standardized extraction form was piloted and then applied by two reviewers (one extractor, one verifier). Extracted items included:

- Study characteristics: year, country, setting, design (parallel/crossover), sample size, inclusion/exclusion criteria.
- Participant characteristics: age, sex/gender distribution, caffeine habit/typical intake, relevant clinical status, sleep restriction/manipulations if present.
- Caffeine exposure: form (capsule vs. beverage), dose (mg and/or mg/kg), timing relative to task, abstinence/washout instructions, confirmation methods (e.g., salivary/plasma caffeine when reported).
- ERP task and EEG methods: paradigm, stimuli, response requirements; EEG montage.
- ERP outcomes: component amplitude/latency (and variability), statistical contrasts, and direction of effect (caffeine vs. comparator). Where numerical data were not directly reported, values were extracted from figures, when possible, with digitization procedures documented.
- Behavioral outcomes (secondary): task performance indices reported alongside ERPs (e.g., reaction time, accuracy/error rate, omissions/commissions) were extracted when available to contextualize ERP effects and to evaluate whether ERP changes occurred with or without behavioral change.
- Data extraction was performed using a standardized form designed specifically for this review and piloted on a subset of included studies before full extraction. One reviewer extracted the data, and a second reviewer independently verified the extracted information against the full text. Discrepancies were resolved by discussion, with third-reviewer adjudication if required. When studies reported multiple ERP outcomes, priority was given to prespecified task-relevant ERP components and contrasts comparing caffeine with placebo or another no-caffeine comparator. For crossover studies, within-participant contrasts were extracted where available. For studies with multiple doses, each dose was extracted separately when reported. When results were presented only graphically, numerical values were extracted from figures where possible and labeled as estimated values.

### 2.7. Risk of Bias Assessment

For randomized trials, risk of bias was assessed using the Cochrane RoB-2 tool, including the RoB-2 extension for crossover trials when applicable. For nonrandomized controlled studies (e.g., quasi-experiments), risk of bias was assessed using the ROBINS-I tool. Risk of bias assessments were conducted independently by two reviewers, with disagreements resolved by consensus or third-party adjudication. We used risk of bias judgments to inform interpretation and (where feasible) sensitivity analyses.

### 2.8. Data Synthesis (SWiM)

Because quantitative pooling was not planned, we conducted a structured framework synthesis using the SWiM framework. Studies were grouped a priori by task domain (sensory, attention/oddball, inhibition/conflict, feedback/reward, language, and change detection) or by ERP component family (early sensory components; mid-latency control/selection components; late positive components; error/feedback-related components). Within each group, we summarized effects separately for amplitude and latency and coded the direction of effects (caffeine increase/decrease/no apparent change) relative to the comparator. Behavioral outcomes were summarized descriptively (without pooling) to indicate whether consistent performance changes accompanied ERP effects or were primarily neurophysiological. Given the heterogeneity of included paradigms, ERP findings were interpreted primarily within the functional context of each task rather than by assuming equivalence of similarly named components across studies. Cross-study comparisons were therefore made only at the level of broader processing domains—such as sensory encoding, attentional allocation, stimulus evaluation, inhibitory control, or preparatory control—rather than treating, for example, oddball P3, No-Go-P3, and resting-state measures as directly interchangeable indices. Our conclusions should thus be read as evidence for context-dependent modulation of these broader processing stages, rather than as a single, paradigm-independent ERP signature of caffeine.

## 3. Results

Figure 1 summarizes the study selection process. Searches across bibliographic databases and supplementary methods (reference lists and forward citation screening) identified 761 records. After de-duplication (n = 554), 207 records remained for title/abstract screening. At this stage, 56 records were excluded because they did not meet eligibility criteria (most commonly because they lacked a controlled caffeine exposure contrast and/or did not report ERP outcomes). The remaining 151 records underwent full-text assessment, and 85 were excluded because they did not report ERP measures derived from scalp EEG/or did not provide an eligible caffeine comparator. This yielded 66 records meeting the core inclusion criteria. During final eligibility verification—applying the prespecified rules for isolable caffeine contrasts and interpretable ERP outcomes—three additional records were excluded: one study in which caffeine was co-administered with another psychoactive substance without a caffeine-isolating contrast, one study whose primary outcomes were brain–computer interface (BCI) classification/features rather than conventional ERP component measures, and one study in which transcranial magnetic stimulation (TMS) was integral to the experimental contrast such that effects could not be attributed to caffeine alone. In total, 63 studies were included in the review and are summarized in Supplementary Table S1 [21,22,23,24,25,26,27,28,29,30,31,32,33,34,35,36,37,38,39,40,41,42,43,44,45,46,47,48,49,50,51,52,53,54,55,56,57,58,59,60,61,62,63,64,65,66,67,68,69,70,71,72,73,74,75,76,77,78,79,80,81,82,83].

To improve interpretability across heterogeneous paradigms, included studies were stratified a priori along two complementary axes: (i) task domain (sensory, attention/oddball, inhibition/conflict, feedback/reward, language, and change detection) and (ii) ERP component family (early sensory components, mid-latency control/selection components, late positive components, and error/feedback-related components). Within each stratum, findings were summarized separately for amplitude and latency, with attention to dose, timing, abstinence/withdrawal control, sleep/fatigue state, and habitual caffeine use.

Across the included studies, terminology for the late positive complex varied (e.g., “P3,” “P300,” “P3b,” “P3a/novelty-P3,” or “late positive complex”). In this review, we use P3b to refer to the task-relevant target-locked parietal positivity typically maximal over centro-parietal sites (often Pz/CPz), and P3a (or novelty-P3) to refer to the fronto-central orienting-related positivity elicited by novel/distractor or No-Go events. When a study did not clearly distinguish subcomponents, we retain the authors’ label (e.g., “P300”) but interpret it using the reported paradigm and scalp distribution.

### 3.1. Participant Characteristics

Across the caffeine–ERP literature, participants are most commonly healthy young adults recruited from university populations or the surrounding community, a strategy that reduces age-related variability in ERP amplitude and latency and supports within-subject pharmacological designs (e.g., refs. [22,24,29,32,36,50,51,53,54,55,56,61,73,75,76]). Typical age ranges cluster from the late teens through the early thirties, with several studies reporting mean ages in the early twenties to early thirties [22,24,29,32,36,50,51]. Sample sizes vary substantially, from small electrophysiology cohorts (e.g., ~9–16 participants) [26,29,34,50,51] to medium laboratory samples (around 20–40 participants) [23,32,37,41,44], and occasionally larger between-subject experiments (e.g., n = 94) designed to test caffeine in combination with other factors such as alcohol in social drinkers [28].

Eligibility criteria across studies are typically conservative, reflecting the sensitivity of late cognitive ERPs (especially P3/P300) to baseline neurophysiological and psychological heterogeneity. Participants are commonly required to be in good physical and mental health, free from neurological or psychiatric disorders, and not taking psychoactive medication; normal or corrected-to-normal vision or hearing is usually required, depending on whether visual or auditory paradigms are used [23,29,30,31,32,34,43,49,50,51]. Many studies also exclude individuals with medical contraindications to caffeine (e.g., cardiovascular conditions or caffeine hypersensitivity), both for participant safety and to improve the interpretability of arousal-linked ERP effects.

Handedness and smoking status are also frequently controlled. Many caffeine–ERP studies restrict participation to right-handed individuals to reduce variability related to hemispheric organization and to stabilize midline ERP measures [25,29,31,34,38,41,49,50,51,53,56,61,75,76,80]. Smoking is likewise often an exclusion criterion because nicotine can independently affect attention and ERP indices and may interact with stimulant effects [53,54,56,61]. Sex composition varies across the literature and is sometimes dictated by experimental aims: several studies used all-male samples (e.g., athletes or student cohorts) [21,25,37], and others focused exclusively on women to control sex-related variability in drug response or ERP amplitude [24,36,81], while many used mixed-sex samples with modest, often balanced, representation (e.g., refs. [30,31,34,41]).

Although the “healthy young adult” profile dominates, the evidence base also includes targeted samples designed to test caffeine effects under specific constraints. Athletic cohorts include competitive male taekwondo athletes characterized as low-habitual caffeine consumers [21] and endurance-trained young men tested during exercise-related preparation [40]. Fatigue and sleep loss contexts are prominent as well, including studies in sleep-deprived participants selected for minimal or absent habitual caffeine use [25] and mixed or moderate-consumption samples tested after extended wakefulness [31]. Other work has contrasted poor versus normal sleepers matched on age and sex [23] or examined caffeine’s influence on cognitive control in young adult social drinkers and in combined caffeine–alcohol conditions [28]. Beyond typical student cohorts, developmental and aging studies broaden the range considerably: pediatric samples include children aged ~8–12 years (including clinical hyperkinetic samples withdrawn from stimulant medication, and typically developing children screened for learning/neurodevelopmental problems) [48,52]. In contrast, older-adult work includes healthy participants spanning roughly the sixth to eighth decades of life [46].

Habitual caffeine exposure is treated as a meaningful participant characteristic because tolerance, withdrawal relief, and expectancy can influence acute effects. Many studies recruit regular but moderate coffee drinkers (often described as roughly 1–3 cups/day) [22,26,41] and implement abstinence periods intended to limit both acute caffeine carryover and severe withdrawal. Other studies include heavier habitual consumers (e.g., 3–6 cups/day) [29,49,50,51], explicitly select low or infrequent users to minimize tolerance and withdrawal confounds [38,43], or stratify participants into high- and low-consumption groups to test baseline differences or dose sensitivity [63]. Withdrawal-focused work, by design, recruits habitual users and imposes longer abstinence intervals to characterize neurophysiological change during cessation [57,60]. Across studies, habitual intake is typically assessed using brief inventories that cover familiar caffeine sources (coffee, tea, energy drinks, cola, etc.) and is sometimes used as a moderator in analyses [77,78].

Pre-session controls are a consistent feature across studies and are used to standardize arousal state and reduce contamination from other psychoactive substances. Participants are often instructed to abstain from caffeine for practical intervals such as 4–12 h [24,29,32,33,34,49,50,51] or, in repeated-measures designs, for more stringent 12–24 h [26,53,56,59,61,73,75,76,79], and to avoid alcohol and other stimulants over comparable periods [23,26,30,31,40,53,56,58]. Many studies also monitor recent sleep timing/quality because fatigue and time-on-task can alter P3/P300 timing and amplitude [56,58,64,74]. Where feasible, compliance is supported by standardized session timing, documentation of last caffeine intake, and (in some studies) biological verification (e.g., salivary caffeine) [53,61,76]. These design features are essential because caffeine can exacerbate anxiety symptoms and alter early auditory processing in anxiety-prone groups, which can complicate interpretation if the baseline state is not well-characterized [83].

Finally, as in most ERP research, the analyzed samples reflect both eligibility and data-quality constraints. Across studies, exclusions are commonly due to excessive EEG artifacts (e.g., eye blinks, muscle noise) or behavioral noncompliance (e.g., unusually high error rates), and final analyzed sample sizes may differ across measures depending on component estimability or artifact rejection procedures [53,56].

Overall, participant characteristics across the caffeine–ERP evidence base reflect a literature dominated by carefully screened, mostly non-smoking young adults with controlled stimulant exposure, complemented by targeted cohorts (athletes, sleep loss groups, alcohol co-administration samples) and broader lifespan samples (children and older adults) designed to probe how context and baseline state shape caffeine-related modulation of ERP components [21,22,23,24,25,26,27,28,29,30,31,32,33,34,35,36,37,38,39,40,41,42,43,44,45,46,47,48,49,50,51,52,53,54,55,56,61,73,75,76,77,78,79,83].

### 3.2. ERP Paradigms

Across the reviewed caffeine studies, ERPs were elicited using a set of well-established paradigms that index partly distinct stages of information processing—from early sensory–perceptual encoding, through attentional selection and stimulus evaluation, to executive control, performance monitoring, and anticipatory/motor preparation [21,22,23,24,25,26,27,28,29,30,31,32,33,34,35,36,37,38,39,40,41,42,43,44,45,46,47,48,49,50,51,52,53,54,55,56,57,58,59,60,61,62,63,64,65,66,67,68,69,70,71,72,73,74,75,76,77,78,79,80,81,82,83]. Most experiments used double-blind placebo-controlled designs and targeted canonical ERP components whose functional interpretations are relatively stable across tasks (e.g., P1/N1/P2 for early sensory–attentional processing; N2 and related negativities for control demands; the P3/P300 family for stimulus evaluation and context updating; and slower potentials indexing preparation) [21,22,23,24,25,26,27,28,29,30,31,32,33,34,35,36,37,38,39,40,41,42,43,44,45,46,47,48,49,50,51,52,53,54,55,56,57,58,59,60,61,62,63,64,65,66,67,68,69,70,71,72,73,74,75,76,77,78,79,80,81,82,83]. Paradigm structure (target rarity, inhibition demands, conflict, cueing, load, time-on-task) was frequently leveraged to isolate specific cognitive operations, with component quantification typically based on peak amplitude/latency (often at midline sites) and, in some cases, area-based or multivariate measures [53,54,55,74].

Oddball target detection paradigms were the dominant approach, implemented in both auditory and visual modalities [21,22,26,27,30,31,38,41,53,56,57,58,74,78]. Participants discriminate infrequent targets embedded among frequent standards, responding with a button press or by counting targets [21,22,53,57]. The principal outcome was usually the P3/P300 (often P3b), measured at midline electrodes (classically Fz/Cz/Pz) as the most significant late positive deflection in a task-defined window (commonly ~250–550 ms), with latency interpreted as stimulus evaluation timing and amplitude as attentional resource allocation/context updating [21,22,30,31,38,53]. Auditory oddball paradigms were used to index cognitive resilience in sport/fatigue contexts [21], to track P300 evolution across repeated blocks or sessions [26,27,56,74], to contrast pharmacological caffeine effects against expectancy/ritual effects by comparing caffeinated versus decaffeinated coffee under matched sensory cues [41], and to examine P300 dynamics under withdrawal/abstinence states or late-night fatigue challenges (including multi-ingredient energy drinks) [57,58]. Visual oddball tasks served similar aims, often emphasizing P3 latency/amplitude (and occasionally fractional area latency or area under the curve) as well as links to reaction time [22,30,31,53,56,74]. Some studies embedded oddball EEG within simultaneous EEG-fMRI, preserving the same target-versus-standard ERP logic while enabling comparisons to BOLD responses [38]. Extensions included compact “brain vital signs” auditory batteries that paired oddball tones (for N100 and P300) with semantic word-pair manipulations (for N400) [78], and analytic innovations such as multivariate decoding to assess whether supplementation stabilizes scalp patterns in the P2/P3 ranges as fatigue accumulates [74].

A second significant cluster of paradigms targeted response execution versus inhibition and broader executive control, most prominently Go/No-Go tasks [25,32,52,54,55]. Visual Go/No-Go designs (including sleep deprivation contexts) typically analyzed P2 (early perceptual/attentional engagement), N2 (conflict/inhibition), and P3 (higher-order evaluation/inhibitory success) separately for Go and No-Go trials across frontal, central, and parietal regions [25]. Auditory Go/No-Go tasks—often using equiprobable tones—were beneficial for decomposing processing into early sensory N1, attention-related processing negativity, and later P3 subcomponents differentiating Go-related response production (P3b) from No-Go-related inhibition (fronto-central No-Go-P3/P3a-like activity) [32,52,54,55]. Several studies explicitly addressed component overlap using separation approaches such as principal components analysis, mapping caffeine-related effects onto distinct stages rather than a single peak [54,55]. Developmental work extended this logic to children with equiprobable auditory Go/No-Go designs that extracted multiple components (including distinct N2 subcomponents) to test stage-by-stage models of categorization, preparation, and control [52].

Closely related, conflict and interference paradigms were used to probe medial–frontal control dynamics [28,68,73,77,82]. The flanker task was employed to evoke a fronto-central N2 (typically ~220–320 ms), sensitive to compatibility (compatible vs. incompatible) and to sequential adaptation (previous-trial compatibility), allowing tests of caffeine effects on both conflict monitoring and trial-to-trial control adjustments [28]. Stroop paradigms provided another interference framework: many studies focused on stimulus-locked P300 as an index of attentional allocation during conflict, sometimes alongside components linked to interference resolution (e.g., N450 or later posterior positivities) [77]. Some Stroop studies treated habitual caffeine use as an individual-difference predictor rather than an acute manipulation [77]. Others used unconventional delivery routes (e.g., mouth rinse or nasal spray) to dissociate pharmacological from orosensory/expectancy mechanisms, occasionally adding source-localization methods during administration intervals to capture rapid state changes that may be reflected in averaged ERPs [68,82]. Cued reward–conflict variants further linked preparatory activity (CNV, contingent negative variation) to downstream conflict processing [73].

To examine selective attention, paradigms included both spatial and nonspatial/feature-based designs, as well as broader search/load manipulations [29,49,51,61,67,70]. Spatial-selective attention tasks instructed participants to attend to one visual hemifield and respond to rare targets at the attended location, producing classic attention effects on early visual P1/N1 at lateral occipital sites (often contralaterally enhanced) and later modulations (P2/N2), with caffeine evaluated for amplitude changes, attention interactions, and potential latency shifts [51]. Nonspatial feature-based attention paradigms (e.g., attending to color) enabled attention difference waves (attended minus unattended) that reveal early selection signatures (frontal selection positivity, posterior selection negativity) followed by N2b/P3b effects; caffeine was tested for its impact on attention-specific differentiation (e.g., N2b attended–unattended separation) versus attention-independent “exogenous” enhancements (e.g., an overall frontal P2 increase) [29]. Related visual tasks manipulated physical stimulus dimensions (e.g., spatial frequency/orientation) to separate exogenous ERP effects from endogenous relevance effects via subtraction logic, and some also quantified motor preparation using lateralized readiness potentials [49]. Visual search and load paradigms required the detection of target letters under focused versus divided attention and low versus high display load, yielding early sensory components (P1/N1), search-related negativities, and a later parietal P3b reflecting categorization; caffeine was evaluated for whether it modulates early registration, search efficiency, or primarily late evaluation timing/strength [61]. Naturalistic discrimination tasks (e.g., detecting rare “imminent accident” traffic scenes) retained the P3b logic while increasing ecological validity and linking ERP shifts to selective attention performance under sleep deprivation and/or combined ingredient administration [67]. Dual-task frameworks paired a central discrimination with demanding peripheral monitoring to test dose-dependent shifts in P3-indexed resource allocation under high workload [70].

A further set of studies emphasized sustained attention and continuous information processing, embedding ERP extraction within longer tasks [24,43,46,50,64,65]. Rapid information processing paradigms (RIP/RVIP) required continuous monitoring of digit streams for predefined sequences; ERPs time-locked to correct detections were often summarized as a late positive complex (~250–500 ms) indexing evaluation during continuous performance, with amplitude/latency compared across doses or beverage conditions (including caffeinated coffee vs. decaffeinated variants with other constituents) [24,46]. Self-paced concentration/vigilance tasks (e.g., Bourdon-type computerized paradigms) averaged ERPs across time windows for all stimuli. They frequently highlighted early frontal positivity (around 200–240 ms) and later parietal positivity (P3 windows), enabling explicit tests of time-on-task effects and lapse-related signatures even when overt performance changed little [50]. The Attentional Network Task provided a structured, sustained attention context with cueing manipulations; the early occipital N1 (~80–140 ms) served as a sensitive index of early engagement across cue conditions, allowing evaluation of whether caffeine produces global sensory–attentional enhancement versus selective modulation of alerting/orienting networks [43]. Long-latency auditory-evoked potential/vigilance designs (N1-P2-N2 complexes) were also used to study attention toward irrelevant auditory input during prolonged performance [64,65].

Beyond stimulus-locked processing, multiple paradigms targeted anticipatory attention, preparation, performance monitoring, and motor-related activity [33,39,40,47,69,71,72,75,76]. For performance monitoring, demanding task-switching procedures were used to elicit errors and quantify the error-related negativity (ERN; peaking within ~0–150 ms after incorrect responses) and subsequent error positivity (Pe; ~200–400 ms), typically maximal at fronto-central sites, enabling tests of whether caffeine modulates post-response monitoring independently of accuracy changes [33]. For preparation, the principal paradigm was the warned reaction time design used to elicit the contingent negative variation (CNV), a slow negativity developing between a warning cue (S1) and an imperative stimulus (S2); these studies tested whether caffeine alters preparatory allocation and whether effects depend on traits such as extraversion or emotional stability [69,71,72]. Cued cognitive control extensions embedded preparation in richer contexts: cued reward-Stroop designs used CNV to index cue-triggered preparatory control and later positivities to capture downstream evaluation effects [73], while task-switching paradigms quantified cue-locked P3 activity plus sustained slow negativities during cue–target or response–stimulus intervals as markers of anticipatory control and task set updating, enabling dose-based tests of whether caffeine selectively strengthens switch preparation [75,76]. Motor preparation and effort-related neurophysiology were examined with movement-related cortical potentials/readiness potentials (Bereitschaftspotential), time-locked to self-initiated actions (e.g., dorsiflexion, pedaling kicks, intermittent contractions), quantified via slow premovement negativity onset, amplitude at defined premovement times, and/or area/power, typically maximal at central midline sites (Cz/FCz) with broader topographic mapping [39,40,47].

Finally, several studies used specialized auditory ERP protocols to index early filtering and pre-attentive change detection [63,64,65,80]. The P50 paired-click sensory gating paradigm assessed suppression of the second click (S2) relative to the first (S1), often summarized as the S2/S1 ratio, an index of early gating [63]. The mismatch negativity (MMN) paradigm isolated automatic deviance detection (~100–250 ms), sometimes within selective attention listening contexts and sometimes in interaction designs testing whether caffeine counteracts drug-related slowing of deviance processing ([80]). Together, these paradigms span early sensory responsiveness and gating (P50, N1/P2, MMN) in [63,64,65,80], selective attention and stimulus evaluation (P3 family, N2b) [21,22,29,30,31,32,38,41,49,51,53,56,61,67,70,74,78], executive control and adaptive monitoring (N2, conflict/interference components, ERN/Pe) [28,33,77], and sustained/anticipatory control plus motor preparation (CNV, cue-locked activity, readiness potentials) [39,40,47,69,71,72,73,74,75,76], allowing the caffeine–ERP literature to localize where, within the processing stream, caffeine-related changes most reliably emerge [21,22,23,24,25,26,27,28,29,30,31,32,33,34,35,36,37,38,39,40,41,42,43,44,45,46,47,48,49,50,51,52,53,54,55,56,57,58,59,60,61,62,63,64,65,66,67,68,69,70,71,72,73,74,75,76,77,78,79,80,81,82,83].

### 3.3. Caffeine Interventions

Across the included ERP studies, caffeine interventions were implemented in several acute and abstinence-based formats that varied by dose expression (fixed vs. weight-adjusted), delivery vehicle, timing, and whether caffeine was administered alone or within combined or multi-ingredient formulations [21,22,23,24,25,26,27,28,29,30,31,32,33,34,35,36,37,38,39,40,41,42,43,44,45,46,47,48,49,50,51,52,53,54,55,56,57,58,59,60,61,62,63,64,65,66,67,68,69,70,71,72,73,74,75,76,77,78,79,80,81,82,83]. Most experiments delivered caffeine as a short-term, controlled acute challenge on a single test day under double-blind, placebo-controlled conditions, most often using within-subject cross-over designs so participants completed both caffeine and placebo sessions separated by a washout (commonly ~1 week to ~1 month, or longer) [21,22,25,26,27,29,30,32,33,34,35,36,38,40,41,42,43,47,49,50,51,53,54,55,58,61,62,66,67,69,70,71,72,73,75,76,80,81,82]. A smaller subset randomized participants between beverage/drug conditions (e.g., caffeine vs. placebo and, in some cases, alcohol combinations) [28,41,44,45]. To reduce expectancy and sensory confounds, caffeine was frequently delivered as capsules matched with placebo capsules [30,31,32,43,54,55,63,67,72,83] or added to decaffeinated coffee with matched sensory characteristics (sometimes paired with deception that “normal coffee” was being consumed) [24,26,29,33,34,35,49,50,51,59,61,62,70,73,75,76]. Other protocols used a coffee beverage with quantified caffeine content (e.g., one cup) [39], aqueous “pure caffeine” solutions with masking agents [79], or functional/multi-ingredient drinks and supplements intended to model commercially relevant products (e.g., caffeine combined with glucose or citicoline; energy drink-like mixtures; botanical/vitamin “energy” supplements; L-theanine + caffeine with additional comparators such as tea) [44,45,56,58,66,74]. A smaller set tested non-ingestive sensory delivery to decouple oral/nasal pathways from standard gastrointestinal absorption, including caffeine mouth rinses and caffeine nasal sprays in placebo-controlled crossover sessions [68,82]. Finally, withdrawal/resumption paradigms formed a distinct intervention class: instead of acute dosing, habitual users completed multi-day caffeine abstinence (and sometimes blinded reintroduction/resumption), with ERPs tracked repeatedly across withdrawal days [57,60].

Dose selection spanned low-to-high ranges but clustered into practical bands. Many studies used moderate fixed doses around 200–250 mg to approximate everyday real-world consumption while simplifying blinding [21,29,32,35,42,43,49,50,51,54,55,59,62,64,72,73,81]. Others used higher fixed doses (e.g., ~400–500 mg) in challenge or fatigue-countermeasure contexts, including clinical/reactivity probes [22,25,27,30,31,60,83]. Lower fixed doses (e.g., ~100 mg) were used in interaction- and mechanism-oriented designs [63,80], and some supplement/energy-product studies used commercially plausible low amounts (e.g., ~55 mg) or energy-drink-equivalent doses (e.g., ~80 mg) within ingredient mixtures [56,58,74]. Many protocols instead expressed dosing by body mass to standardize exposure, typically ~2.5–6 mg/kg (often 3 mg/kg), with dose–response extensions that compared multiple weight-adjusted doses (e.g., 1.5/3.0/6.0 mg/kg; 3 vs. 5 mg/kg; up to ~7.5 mg/kg) [23,24,26,28,33,34,35,36,38,40,41,47,49,53,61,70,73,75,76,79]. Children’s studies used lower absolute amounts appropriate to age/body size (e.g., ~80 mg or ~3–6 mg/kg) [48,52]. In fatigue/sleep loss countermeasure protocols, caffeine was sometimes delivered as a single bolus or as split/maintenance regimens (e.g., an initial dose followed by a smaller follow-up dose, or a total dose distributed across two administrations) to cover prolonged deprivation and repeated testing [21,25,31,35,42].

Most protocols standardized pre-session abstinence from caffeine (and often alcohol/other stimulants and sometimes smoking) to control recent intake and baseline state, with abstinence windows commonly ~10–24 h, though some used shorter restrictions of only a few hours to reduce withdrawal while preserving ecological validity [23,26,29,30,31,32,33,34,35,38,49,50,51,53,54,55,56,58,59,61,64,70,73,75,76,79]. In a subset—particularly when attempting to normalize baseline sensitivity across clinical groups—abstinence was extended up to about a week [83], and compliance was occasionally verified biochemically (most often via salivary caffeine measures) in weight-adjusted oral dosing paradigms [53,61,73,76]. ERP testing was typically scheduled to match expected pharmacokinetics, with recordings usually beginning ~30–60 min post-ingestion (and sometimes explicitly ~40–50 min), although several studies collected multiple post-dose time points (e.g., early/late assessments over hours; baseline/~30 min/~90 min) or repeated ERP sampling across extended vigilance/time-on-task periods (e.g., ~2 h) to map the time course of effects [21,23,26,27,32,36,38,43,50,52,56,63,64,67,74,79].

Participant selection and habitual caffeine use were frequently treated as intervention-relevant characteristics because tolerance and withdrawal reversal effects can shape acute responses. Samples ranged from non/low consumers [21,25,38,43], to moderate habitual users (often ~1–2 cups/day) [22,26,31,32,36,49,54,55,56,59,61,62,70,73,75,76,79], to heavier habitual users (e.g., ~3–6 cups/day) [29,50,51]. Some studies explicitly recruited habitual coffee drinkers to examine caffeine within realistic consumption contexts or to separate caffeine pharmacology from coffee cues [41], contrasted high vs. low-habitual users as a factor (e.g., sensory gating) [63], deliberately included both users and non-users to limit withdrawal reversal interpretations [58], or operationalized exposure via habitual intake/deviation from usual intake in field-like contexts (e.g., athletes) [78]. Finally, caffeine was often embedded within broader state manipulations or factorial frameworks to test countermeasure or interaction hypotheses while keeping caffeine itself as the controlled intervention: fatigue, sustained time-on-task, exercise workloads, and total sleep deprivation paradigms [21,25,26,31,35,40,42,47,50]; combined/interacting conditions such as caffeine with alcohol [28,41,80,81], caffeine with motivational/reward manipulations [73], caffeine in multi-drug comparator sets (e.g., alongside d-amphetamine or benzodiazepines) [69,71,72], or caffeine paired with other exposures (e.g., UMTS mobile-phone exposure) in multi-condition crossover designs [53].

The classification of caffeine dosages in the reviewed literature is primarily harmonized through two metrics: body weight-dependent dosing (mg/kg) and fixed absolute dosing (mg), often cross-referenced with “cup of coffee” equivalents. Low doses are generally defined as approximately 3 mg/kg of body weight or as absolute amounts ranging from 55 mg to 200 mg. Specifically, 3 mg/kg is frequently considered a low dose because it mimics the caffeine content of about 1 cup of brewed coffee. Some research further refines this by identifying 1.0 to 1.5 mg/kg as the lower end of the low-to-moderate spectrum.

Moderate doses are typically around 3.0 mg/kg or in fixed doses of 200 mg to 250 mg. For instance, 250 mg is often described as a moderate dose equivalent to approximately two strong cups of coffee. In studies focusing on sustained attention and auditory processing, these moderate levels are used to identify selective enhancements in neural processing without the overstimulation sometimes associated with higher amounts.

High doses are consistently defined across the literature as 5.0 mg/kg to 6.0 mg/kg or fixed amounts of 400 mg to 500 mg. At these levels, dosages are often compared to consuming three or more cups of coffee or multiple energy drinks. The most extreme “high” dose cited in the provided studies was 7.5 mg/kg, which significantly sped reaction times but also showed signs of a performance plateau or overstimulation. The goal across these studies is achieved by utilizing weight-based dosing to ensure pharmacological consistency across diverse participant groups, particularly when comparing athletes, children, or different age groups. While absolute doses like 250 mg or 400 mg are common in clinical trials involving healthy adults, they are often contextualized by the participants’ habitual intake, ranging from low consumers (<150 mg/day) to moderate or heavy users (300–440 mg/day), to account for potential tolerance or withdrawal effects. These standardized benchmarks allow researchers to compare electrophysiological outcomes, such as P300 and N2 amplitude modulation, across various cognitive tasks and physiological states.

#### Acute Versus Habitual Caffeine Exposure and Consumer Status

Most included studies examined acute caffeine administration, usually as a single dose given shortly before ERP recording. These studies therefore primarily inform the short-term neurophysiological effects of caffeine on sensory processing, attentional allocation, stimulus evaluation, response preparation, and performance monitoring. Acute doses varied substantially across studies, ranging from low doses approximating one cup of coffee to higher laboratory doses of approximately 3–6 mg/kg or 200–500 mg.

A smaller subset of studies addressed repeated short-term exposure, particularly in fatigue or sleep deprivation paradigms where caffeine was administered more than once or used to maintain alertness across prolonged wakefulness. These studies were interpreted separately from single-dose rested-state experiments because caffeine effects under sleep loss or sustained task demand may reflect the restoration of impaired processing rather than enhancement above baseline.

Habitual caffeine use also varied across studies. Many experiments recruited regular or moderate caffeine consumers, often with short pre-session abstinence periods. In such designs, acute caffeine effects may reflect a combination of direct stimulant action, relief of mild withdrawal, and tolerance-related attenuation. Other studies recruited low or infrequent users to reduce withdrawal and tolerance confounding, while withdrawal-focused studies specifically examined ERP changes during abstinence or caffeine resumption. Because definitions of “regular,” “moderate,” “low,” and “occasional” use were not standardized across studies, habitual intake was treated as a moderator and interpreted qualitatively rather than pooled quantitatively.

### 3.4. P300 Amplitude

Table 1 presents a component-level summary of consistent and inconsistent caffeine-related EP/ERP findings.

Across the ERP literature, caffeine’s most frequently reported amplitude effect involves the P300 complex (P3/P3b), generally interpreted as reflecting the intensity of attentional resource allocation and context updating for task-relevant events. However, the direction and robustness of this effect are not uniform: outcomes depend strongly on baseline state (fatigued/sleep-restricted vs. rested), task demands, dose, and repetition across blocks or sessions. Under conditions that tax alertness or require sustained effort, caffeine most often acts as a resource-supporting modulator, preserving or augmenting late positive activity when it would otherwise be reduced. For example, in taekwondo athletes exposed to combined mental and physical fatigue, low-dose caffeine (~200 mg; ~3 mg/kg) selectively protected/enhanced post-fatigue P300 amplitude with site specificity (larger increases at Cz and Pz but not Fz), consistent with maintained resource allocation rather than faster stimulus evaluation [21]. Parallel patterns appear in fatigue and sleep loss manipulations, in which P3 amplitude is typically depressed: caffeine tends to restore or increase later positivity during task-relevant processing when participants are tired [42], and in choice reaction paradigms, P3 amplitude enhancements are often most evident when placebo amplitudes are diminished by fatigue [35]. Similar “neural buffering” emerges in sustained attention settings in which behavioral benefits are weak or absent: caffeine can increase parietal P3 amplitude in later windows during self-paced concentration despite minimal performance change [50], and in vigilance-style attention, caffeine increases the Attention-P3 amplitude and global field strength during correct detections, even when overt performance does not reliably improve [62]. In response control paradigms, caffeine’s amplitude effects are frequently component- and function-specific rather than global: in auditory Go/No-Go tasks, caffeine robustly enhances Go-related P3b over centro-parietal sites with little or no latency shift, implying stronger engagement in target evaluation/response selection rather than acceleration [32,54], and PCA-based analyses reinforce this preferential amplification of Go-P3b activity [55]. Some studies also report broader oddball-related P3 amplitude increases, including short-lived post-ingestion boosts (e.g., ~30 min after 500 mg) that counteract placebo-related declines suggestive of habituation or waning alertness across repeated recordings [27], and within-subject oddball findings showing post-caffeine amplitude increases at Fz, Cz, and Pz [37]. Even where placebo and caffeine groups are difficult to separate at the group level, there are indications of localized late-component amplification—e.g., in habitual coffee drinkers, ingestion influenced late activity at Cz regardless of caffeine expectancy, but only caffeine produced a statistically reliable increase across the broader N2-P3 interval [41].

At the same time, multiple experiments demonstrate that caffeine does not reliably increase P300 amplitude in all contexts. Several visual oddball studies show little to no amplitude change despite latency shortening (often at frontal sites), suggesting caffeine can act as a temporal accelerator without magnifying response size [22,30]. Similarly, some deprivation protocols report stable P300 amplitude even when sleep loss would be expected to reduce it [31], and dose–response work in rapid information processing tasks finds no systematic late positive amplitude change across a wide dose range [24]. Converging with this dissociation, simultaneous EEG-fMRI oddball work reports marked caffeine-related reductions in P300 latency, with amplitude remaining unchanged [38], and visual search manipulations of focused/divided attention and display load likewise reveal latency shortening without reliable amplitude enhancement [61]. Significantly, caffeine can also be associated with smaller P300 amplitudes under certain sustained or repetitive conditions: in an auditory oddball task repeated across multiple blocks, caffeine produced reliably reduced P300 amplitude relative to placebo, with the difference growing as repetition progressed—consistent with altered deployment/efficiency of resources across time-on-task rather than simple disengagement [26]. Relatedly, in lengthy procedures where placebo groups show progressive increases in P3 amplitude, interpreted as compensatory effort, low-dose caffeine can attenuate that rise, consistent with a reduced need to upregulate resources to maintain performance [56]. Finally, dose and workload can shape whether amplitude effects emerge and where they localize. In a demanding visual dual-task, P3 peak amplitude increases dose-dependently, with the greatest effects in posterior regions and minimal latency change, suggesting increased late-stage processing “gain” under high load [70].

The relationship between caffeine dosage and P300 amplitude does not always follow a linear pattern. Regarding linear trends, one study utilizing doses of 1.0, 3.0, and 7.5 mg/kg found that P3 amplitude increased significantly as the dose increased, particularly over posterior cortical regions. Other research found that, while caffeine improved behavioral processing speed at low-to-moderate doses (1.5–3.0 mg/kg), these benefits disappeared at higher doses (6.0 mg/kg), and no significant dose–response relationship was found for ERP amplitudes. For instance, in error-related processing, both 3 mg/kg and 5 mg/kg doses increased ERP amplitudes, but there was no significant difference between the low and high doses.

Caffeine’s effects are highly sensitive to the specific cognitive demands of the task. Caffeine has been shown to enhance P300 amplitude in auditory oddball paradigms requiring stimulus discrimination, yet it produced no changes in simpler single-tone tasks that do not require discrimination. In Go/No-Go paradigms, caffeine consistently enhances the Go-P3b component associated with response execution but often has minimal or no effect on No-Go components related to inhibitory control. The benefits of caffeine on P300 amplitude are often more pronounced in high-demand conditions, such as when stimuli are degraded or when participants must perform complex visual dual-tasks. Furthermore, caffeine has been found to increase P300 amplitude in choice reaction time tasks but not in simple reaction time tasks.

### 3.5. P300-P3b Latency

P300/P3b latency emerges as a recurring—though clearly context-dependent—marker of caffeine’s acute effects. Because P300 latency is commonly interpreted as an index of the time required for stimulus evaluation and categorization, earlier P3 timing is typically taken to reflect faster completion of target evaluation rather than simply “more” processing. Many placebo-controlled oddball and attention studies converge on the idea that caffeine can shorten P300/P3b latency, often without parallel amplitude increases and sometimes without significant behavioral gains. In visual oddball paradigms, for example, latency reductions are frequently most evident at frontal midline sites: several studies using relatively high doses (e.g., 400 mg) report significantly earlier P300 peaks at Fz, with more minor or nonsignificant shifts at Cz and little to no facilitation at Pz, suggesting that caffeine’s temporal benefits may be most pronounced in frontal systems that support attentional control and decision processes rather than uniformly accelerating posterior categorization networks [22,30]. Converging evidence comes from auditory oddball work (including simultaneous EEG-fMRI), where caffeine (e.g., 250 mg) substantially shortened P300 latency across midline electrodes while leaving amplitude essentially unchanged—reinforcing the notion of a selective timing shift in target evaluation dynamics [38]. More sensitive analytic approaches strengthen this interpretation: single-trial modeling and alternative latency metrics (e.g., fractional area latency, midpoint latency) show earlier stimulus-locked P3b timing after caffeine, and in at least one case, the latency shift statistically accounted for a meaningful portion of caffeine-related reaction time speeding, whereas response-locked P3b timing was unaffected—implicating earlier evaluative/decision stages rather than motor execution processes [36,53]. Beyond basic oddball detection, similar “speeding” profiles appear in selective attention and search tasks, where caffeine shortens P3/P3b latency most clearly for targets or task-relevant stimulus classes (i.e., once a relevance gate has been passed) and may be strongest when attentional focus is easier to maintain (e.g., lower search load) [49,61].

A related pattern is that caffeine can stabilize P300 latency under fatigue or time-of-day stress, preventing the typical fatigue-related prolongation rather than pushing latency below baseline. Low-to-moderate doses or caffeine-containing mixtures have been reported to transiently reduce P3 midpoint latency shortly after ingestion (with effects fading later), and to prevent late-night fatigue-related P300 slowing in placebo conditions—consistent with caffeine preserving classification speed when arousal would otherwise decline [56,58]. Under high real-world attentional strain (e.g., sleep loss or complex scene discrimination), caffeine-containing combinations have also been associated with robust reductions in P3b latency across centro-parietal sites, again consistent with accelerated or protected target evaluation in demanding contexts [67].

However, P300 latency is not uniformly responsive to caffeine. In some fatigue-plus-sport settings, caffeine increases P300 amplitude after exertion without altering latency at Fz/Cz/Pz, suggesting enhanced resource allocation without faster evaluation speed in that context [21]. Other designs—especially those dominated by sustained-performance constraints, substantial dual-task load, or sleep deprivation conditions—report stable P3 latency despite caffeine, even when amplitude or earlier ERP components shift, suggesting that caffeine’s temporal benefits may be absent when the main bottleneck lies outside late stimulus classification (e.g., structural capacity limits, upstream perceptual gating, or other task-pipeline constraints) [24,31,70,79].

### 3.6. N2 Amplitude

Across the studies summarized, caffeine-related modulation of N2 amplitude is present but clearly heterogeneous and strongly task- and measurement-dependent, in part because “N2” was operationalized in multiple ways (e.g., classic No-Go-N2, attention-linked N2b, or peak-to-peak composites such as P2-N2 or N2–P300). In paradigms emphasizing inhibitory control, caffeine effects on the N2 family range from absent or small to selective, subcomponent-specific changes. In an expectancy-controlled executive function design, caffeine did not reliably modulate No-Go-N2 amplitude, consistent with minimal impact on early conflict detection under those conditions [62]. By contrast, PCA-based Go/No-Go work identified a No-Go-specific P2/N2b complex and found that caffeine reduced the frontal N2b-related negativity (alongside a reduction in the associated P2 positivity), interpreted as reduced inhibitory conflict/effort during No-Go processing [55]. Other Go/No-Go evidence suggests caffeine can increase inhibition-related N2 under challenge: following total sleep deprivation, caffeine modestly preserved inhibitory-stage dynamics, including enhanced frontal No-Go N2 amplitude (and shorter No-Go N2 latencies), consistent with partial maintenance of inhibition/conflict signaling when fatigue would otherwise erode it [25]. Developmental findings further underscore component specificity: in children completing an equiprobable auditory Go/No-Go task, caffeine robustly increased No-Go N2b amplitude while simultaneously reducing a later frontal N2 subcomponent (fN2c), implying a strengthening of core inhibitory operations alongside a dampening of later/ancillary evaluative activity [52]. In rested-versus-fatigued designs, caffeine-related N2 increases were more evident under sleep deprivation or fatigue [42].

In conflict monitoring contexts (e.g., flanker paradigms), caffeine can also heighten the conflict-related N2 signal, with this effect depending on trial history. During a flanker task, caffeine was associated with larger (more negative) N2 responses on incompatible trials and exaggerated the neural compatibility effect following low-conflict trials, suggesting caffeine kept monitoring systems more strongly “online” even when conflict was not expected; importantly, the same pattern implied a shift toward more sustained monitoring rather than improved flexible, trial-by-trial adaptation [28]. Cued preparation and task-switching studies likewise indicate that caffeine’s impact on N2 can reflect contextual reweighting of control signals: caffeine reduced the shift-repetition N2 difference (primarily via effects on repeat trials) in one report [75], whereas another found caffeine increased N2 amplitude across conditions, consistent with a more general enhancement of control-related signaling [76]. Yet not all paradigms show sensitivity: an auditory oddball caffeine ingestion study reported only nonsignificant N2 amplitude increases [79], and an alcohol–caffeine adenosine study found no significant N2b amplitude or latency changes across conditions [80].

Outside classic inhibition/conflict framings, caffeine can enhance N2-like indices of selective attention, though this too depends on how N2 is defined and quantified. In a color-based selective attention paradigm, caffeine amplified the attentional N2b effect by increasing neural separation between attended and unattended stimuli at the N2b stage, indicating a stronger “selection-for-action” contrast without necessarily shifting timing [29]. Similarly, in an aging selective-search study, caffeine increased N2b amplitude in both young and older adults without changing onset latency, aligning with the idea that caffeine boosts energetic resources supporting attentional selection rather than the temporal structure of selection [59]. However, null effects also occur in attention-heavy tasks: in a visual search study, caffeine did not affect the amplitude or timing of the N2/search-related negativity [61].

Finally, studies using peak-to-peak composites (rather than isolating an N2 peak) add further mixed directions that can reflect combined changes across components. During dual-task vigilance with concurrent auditory stimulation, caffeine produced a lower P2-N2 amplitude versus placebo, consistent with suppressed processing of irrelevant auditory input. At the same time, attention remained anchored to the visual vigilance task [64]. In contrast, in an attention study combining L-theanine and caffeine, caffeine increased the N2–P300 peak-to-peak amplitude, suggesting stronger later attentional engagement—though this composite cannot be attributed uniquely to N2 because it partly reflects P3 changes as well [66]. Taken together, the literature supports a clear conclusion: caffeine’s influence on N2 amplitude is not uniform, and is shaped by (i) whether N2 indexes selective attention (often enhanced; e.g., N2b effects in [29,59]) versus inhibition/conflict detection (often minimal, reduced, or selectively altered; e.g., refs. [55,62], but sometimes enhanced under challenge as in [25,42]), (ii) trial type and subcomponent distinctions (e.g., N2b vs. other frontal negativities in [52]), (iii) task context and sequential effects (as in flanker conflict history effects [28] and shift-repeat dynamics [75]), and (iv) whether amplitude reflects an actual N2 peak versus a composite metric (as in [64,66]).

### 3.7. Contingent Negative Variation (CNV) and Preparatory Slow Negativity

Beyond stimulus-locked P3 effects, caffeine also modulates anticipatory ERP activity—slow negative potentials that build up as people prepare for an upcoming event—most prominently the CNV- and closely related preparatory slow negativities in cue–target or response–stimulus intervals. Conceptually, the CNV develops between a warning cue and an imperative stimulus and is commonly linked to expectancy, sustained attention, and motor preparation; empirically, however, caffeine’s influence on CNV/preparatory negativity is less uniform than for many stimulus-locked components, often depending on individual differences, task demands, and how the CNV is quantified.

In warned reaction time paradigms, caffeine can shift the early CNV component in ways that are frequently interpreted as changes in preparatory resource allocation or cortical “efficiency,” but with the critical caveat that the size—and sometimes even the apparent direction—of the effect is strongly moderated by stable person-level factors. When caffeine was compared with a placebo alongside another stimulant (d-amphetamine), both drugs reduced early CNV amplitude overall. Yet, this reduction was markedly larger in more extraverted individuals, indicating that caffeine’s impact on preparatory potentials is not uniform across people but varies with baseline arousal traits, as indexed by personality measures [69]. A within-subject comparison of caffeine and the sedative chlordiazepoxide likewise suggested that early, orientation-related CNV tracks a personality-by-drug pattern rather than a single main effect: caffeine tended to increase early CNV in higher-extraversion participants and decrease it in lower-extraversion participants, whereas the depressant showed the opposite configuration—consistent with the view that caffeine shifts preparatory brain states depending on where an individual lies on an arousal continuum [71]. Extending this emphasis on dynamics and moderation, a study including diazepam, caffeine (200 mg), and placebo reported a biphasic caffeine pattern on early CNV (CNV-I)—a light, early increase followed by later suppression relative to placebo—while the late CNV component nearer the imperative stimulus was comparatively drug-insensitive; critically, emotionally stable versus emotionally labile participants showed opposing CNV-I responses that could cancel out in the group mean, underscoring why CNV effects can be more informative when individual differences are modeled rather than averaged away [72].

In modern cognitive control paradigms, caffeine’s effects on anticipatory negativity often look less like a global amplitude shift and more like selective amplification of preparation when extra control or motivation is engaged. In a reward-cued Stroop design, reward cues elicited a larger CNV than no-reward cues, and caffeine specifically strengthened this reward-related CNV enhancement—producing a clearer reward–no-reward separation under caffeine than under placebo—consistent with caffeine boosting preparatory control, particularly when incentives increase the stakes of upcoming performance [73]. Task-switching studies converge on a similar pattern: caffeine can intensify slow preparatory activity during the preparatory interval, with effects often most evident in switch-specific components rather than in a nonspecific baseline shift. In one cued switching paradigm, caffeine (3 or 6 mg/kg) increased the shift-related slow negativity late in the preparatory window (near target onset), enlarging the switch-repeat difference without depending on whether the switch required reconfiguring one versus two task set elements—suggesting amplification of general anticipatory control rather than task-specific rule updating [75]. In a predictable switching task, caffeine similarly enlarged the switch-repeat difference in late slow negativity (especially over posterior sites), driven mainly by increased negativity on switch trials. In contrast, repeat trials remained relatively stable, consistent with the idea that caffeine enhances neural processes that prepare for a new task set rather than simply shifting overall negativity [76].

At the same time, there are well-characterized contexts in which caffeine does not reliably modulate CNV magnitude, highlighting that CNV can be comparatively insensitive to acute caffeine, even when subjective alertness and behavior change. In a study contrasting self-rated poor sleepers with normal sleepers, CNV magnitude robustly differed between groups—poor sleepers showed consistently larger CNVs and faster responses—yet caffeine (2.5 mg/kg) did not significantly change CNV magnitude or the CNV-reaction time relationship in either group; the time course of CNV across repeated post-dose measurements was essentially parallel under caffeine, implying that observed CNV differences were trait-like rather than driven by acute caffeine sensitivity [23]. A similar null profile was reported in older adults during a sustained attention RVIP task: although caffeinated coffee produced expected subjective and behavioral benefits, a late CNV index at Fz (contrasting late vs. earlier CNV windows) was not reliably altered relative to placebo or decaffeinated conditions [46].

### 3.8. P50 Sensory Gating Ratio

Caffeine can also alter earlier filtering operations, as evidenced by the P50 sensory gating ratio (S2/S1) in the paired-click paradigm. Under typical conditions, the brain suppresses the response to the second click (S2) relative to the first (S1), producing a lower ratio that reflects effective gating of redundant input; a higher ratio indicates weaker suppression and, functionally, a “leakier” sensory filter. Acute caffeine shifts this parameter toward reduced gating. When healthy participants received placebo or escalating oral doses of caffeine (100, 200, 400 mg) across separate sessions, summary measures of the P50 ratio showed a clear dose-related pattern: 200 mg and 400 mg reliably increased the P50 ratio relative to placebo, whereas 100 mg did not differ meaningfully from placebo, indicating a threshold-like effect in which moderate-to-high caffeine doses measurably impair the normal suppression of repeated auditory information [63]. Although the time course across post-dose measurements (15–90 min) was variable enough that a simple dose-by-time interaction was not robust, collapsing across post-dose samples (mean ratio) or focusing on the maximum ratio captured the effect, implying that caffeine’s gating impact can be temporally heterogeneous across individuals even when the overall direction of change is consistent [63]. The ratio increase did not depend on gender or on whether participants were habitual high- or low-caffeine users, suggesting that the acute pharmacological effect can override baseline differences in daily consumption. However, habitual users showed lower baseline S2 amplitudes, consistent with somewhat stronger suppression before any acute dosing [63]. Mechanistically, the gating change appeared to arise from a combined tendency toward a smaller conditioning response (reduced S1) and a larger test response (increased S2), which together push the ratio upward without requiring a latency shift; indeed, P50 latencies were not significantly altered by caffeine, underscoring that this parameter reflects a change in magnitude-based inhibitory regulation rather than the timing of early auditory encoding [63]. In practical terms, the P50 findings complement the P300 literature by showing that caffeine’s ERP footprint is not confined to “more attention” or “faster evaluation”. At sufficiently high doses, caffeine can also measurably weaken an early sensory inhibitory gate, potentially increasing the passage of redundant auditory input into downstream processing even as later-stage performance and evaluative potentials may be enhanced in other tasks.

### 3.9. Mismatch Negativity (MMN) Latency

Another ERP parameter that captures caffeine’s influence at an earlier, largely automatic stage of processing is the MMN peak latency, an index of pre-attentive auditory change detection. In a dichotic listening paradigm designed to probe how alcohol disrupts auditory deviance processing, alcohol reliably delayed MMN generation, producing a clear prolongation of MMN peak latency relative to placebo. Caffeine alone did not significantly alter MMN latency or amplitude in this setup, suggesting that, under baseline conditions, the early deviance detection mechanism was relatively stable and not readily sped up by caffeine. However, when caffeine was co-administered with alcohol, MMN latency was significantly reduced compared with alcohol alone, moving back toward placebo-like values. This pattern implies that caffeine can normalize or counteract a drug-induced slowing of automatic change detection, consistent with the interpretation that adenosine receptor antagonism can mitigate alcohol’s delaying effect on MMN timing. Importantly, this antagonistic action was selective: caffeine did not similarly reverse alcohol-related effects on all later components, indicating that caffeine’s clearest impact here is expressed as protection of the timing of early deviance detection under pharmacological challenge rather than as a generalized speeding of all ERP stages [80].

### 3.10. Mismatch Negativity Amplitude

In the older-adult coffee study that explicitly quantified MMN as the deviant-minus-standard mismatch waveform at Fz, acute ingestion of caffeinated coffee did not reliably change MMN amplitude relative to placebo, and neither caffeinated coffee nor high-chlorogenic-acid decaf differed from regular decaf on this MMN measure, indicating that the automatic detection of auditory novelty remained essentially stable across treatments in that protocol [46]. This kind of null finding fits with the broader impression from several caffeine–ERP datasets that caffeine’s clearest effects tend to emerge in components tied to task engagement, target evaluation, and controlled attention. In contrast, truly automatic sensory discrimination signals can be comparatively resistant to modulation. This interpretation is reinforced by simultaneous EEG-fMRI work showing that early sensory-evoked potentials are preserved under caffeine, even as other neural indices of higher-order processing shift in timing [38].

### 3.11. P2 (Amplitude and Latency)

Across the studies summarized, the P2 component emerges as one of the more frequently reported caffeine-sensitive ERP markers. Still, its modulation is best characterized as context-dependent rather than a uniform “boost.” When caffeine affects P2, it does so either by increasing frontal/fronto-central positivity (amplitude effects)—often in vigilance- or fatigue-challenged settings—or by slightly advancing the timing of the P2 peak (latency effects), most consistently when arousal is compromised or when early classification speed is emphasized. At the same time, several Go/No-Go datasets and component-separation approaches indicate that caffeine can also be associated with reduced P2-like positivity in specific trial types, suggesting that caffeine sometimes reallocates processing across successive stages rather than amplifying every mid-latency component.

A relatively consistent amplitude pattern appears in paradigms where P2 is interpreted as an index of early attentional engagement, perceptual gating, or arousal-linked readiness over frontal sites. In visual Go/No-Go performance after prolonged wakefulness, caffeine enhanced P2 amplitude at frontal electrodes for Go trials. It also increased No-Go P2 over frontal/central regions, consistent with caffeine strengthening early-stage processing that supports sustained responsiveness under fatigue [25]. Similar frontal P2 increases are reported in attention-focused tasks without strong sensory confounds in a color-based selective attention paradigm. Caffeine increased a frontally distributed P2 (roughly in the ~180–280 ms range) in a manner that did not depend on whether stimuli were attended, supporting an interpretation of general early responsivity rather than selective sharpening at this stage [29]. Converging evidence from sustained attention contexts shows the same direction: during a continuous concentration task, caffeine produced a robust increase in mean P2 amplitude across frontal and fronto-central sites (e.g., AFz/Fz/F3/F4), consistent with heightened readiness or stronger recruitment of frontal systems even when overt performance changes were limited [50]. Likewise, in a spatial-selective attention task, caffeine produced a main effect on frontal P2 amplitude across frontal electrodes, while leaving classic sensory attention effects and component latencies largely unchanged, again supporting an arousal-related modulation that coexists with intact sensory selection mechanisms [51]. However, amplitude changes are not always broad or uniform. In an auditory Go/No-Go study, caffeine left the No-Go components essentially unchanged. Still, it produced a focal enhancement of Go P2 amplitude over midline sites, suggesting that P2 amplification can be more prominent during response execution than during inhibitory processing in some paradigms [32].

Importantly, other Go/No-Go evidence indicates that caffeine can be linked to reductions in P2 or P2-like positivity, especially when P2 is embedded within overlapping component structure and interpreted as reflecting how long the system “dwells” in an early evaluation stage. In an equiprobable auditory Go/No-Go task, caffeine was associated with a reduction in Go-related P2 amplitude, most notably over frontal regions, which was interpreted as potentially reflecting more efficient discrimination, allowing processing to advance to later response-related operations with less sustained mid-latency positivity [54]. Similarly, a fine-grained component analysis of a related Go/No-Go design reported that caffeine could reduce the P2-like positivity contained within a composite P2/N2b complex on No-Go trials, consistent with a rebalancing of mid-latency control operations during withholding rather than a simple across-the-board enhancement [55]. These findings are a useful corrective to any single-direction summary: P2 is caffeine-sensitive, but the direction of the effect can depend on whether the task emphasizes sustained vigilance, rapid response execution, or inhibitory control, and on whether analysis methods separate overlapping mid-latency processes.

Latency findings complement the amplitude story by suggesting that caffeine can sometimes accelerate P2 timing, particularly when baseline arousal is reduced. In an auditory oddball study, caffeine produced a general tendency toward shorter latencies across several ERP peaks. Still, the clearest statistical signal was for P2 latency, which showed a significant reduction in the omnibus analysis after caffeine ingestion, even though post hoc electrode-by-electrode patterns were not uniformly significant—implying a modest, distributed timing benefit rather than a strong focal effect [37]. A conceptually similar speeding effect is observed under sleep deprivation: caffeine shortened P2 latencies after prolonged wakefulness, aligning with the view that caffeine can restore or protect early-stage processing speed when fatigue would otherwise slow it [25]. In contrast, in paradigms where caffeine clearly increases frontal P2 positivity (e.g., sustained attention and some selective attention tasks), latency shifts are often absent, suggesting that caffeine can enhance the strength of P2-related activity without consistently shifting its peak [50,51]. Related timing results in other oddball-style work similarly point to subtle P2 latency sensitivity: latencies of multiple components can trend earlier after caffeine, with P2 sometimes standing out as the most detectable timing change in main analyses, again implying that the latency effect—when present—is modest and context-sensitive rather than universal [79].

### 3.12. N100 Family (Including N1-1)

The N1 family—often labeled N1 in visual paradigms and N100/N1 in auditory paradigms—shows caffeine sensitivity that is best described as selective and state-dependent, rather than a uniform “early sensory boost.” When effects appear, they often take the form of amplitude increases (more negative N1), consistent with enhanced early sensory–attentional gain or protection against fatigue-related decline, and, less consistently, modest latency shortening (earlier N1 peak), suggesting slightly faster early encoding. At the same time, both null results and direction reversals occur, especially when the auditory stream is task-irrelevant or when caffeine exposure is defined relative to an individual’s habitual intake rather than experimentally manipulated.

In controlled experimental tasks designed to isolate processing stages, caffeine can sharpen early visual encoding indexed by the N1. In choice reaction paradigms, caffeine increased the amplitude of the visual N1 (particularly over occipital sites). It shortened N1 peak latency by several milliseconds, a combined pattern consistent with enhanced early sensory processing rather than purely later decision-stage speeding [35]. Related evidence from sustained attention designs suggests that caffeine can stabilize early cortical responses over time: in an Attentional Network Task with low-habitual caffeine users, caffeine produced a reliable increase in occipital N1 amplitude from pre- to post-ingestion, whereas placebo showed little change or slight reductions consistent with emerging fatigue, implying that caffeine can maintain or elevate early visual activation during prolonged attentional engagement even when cue-specific attentional patterns remain broadly intact [43]. In other words, caffeine’s most interpretable N1 effects often emerge when the task taxes sustained readiness or when early sensory responsiveness would otherwise drift downward.

In auditory Go/No-Go paradigms, early N1-like activity is sometimes decomposed into subcomponents, most notably N1-1, which is treated as a frontally or vertex-maximal negativity peaking around 100 ms. In the child equiprobable auditory Go/No-Go dataset, N1-1 was described as an early, frontally maximal negativity peaking at roughly ~105–110 ms after tone onset, and caffeine’s effect was condition-dependent: for Go stimuli, changes were small and topographically limited, whereas for No-Go stimuli, caffeine produced a reliable enhancement of N1-1 amplitude, most evident near central midline/vertex sites, consistent with stronger early engagement when the stimulus signaled withholding [52]. This pattern reinforces a broader theme across the N1 family: caffeine can bolster early processing, but it tends to do so when the early signal is behaviorally consequential (here, categorization in service of inhibition), not necessarily as a blanket amplification of the earliest auditory response across all trial types.

Not all auditory N1/N100 findings, however, point toward increased negativity. A naturalistic “brain vital signs” protocol illustrates that caffeine effects on N100 can depend on deviation from one’s personal norm rather than simple presence/absence. In downhill mountain bike athletes, reporting caffeine consumption in the prior 24 h (yes/no) was not meaningfully associated with N100 measures, but reporting more caffeine than usual was linked to a specific N100 profile: smaller N100 amplitude alongside earlier N100 latency relative to riders whose intake was closer to habitual levels [78]. This combination—reduced magnitude but faster timing—underscores that early auditory responsiveness can shift in more than one direction depending on how caffeine exposure is framed (acute experimental dosing versus real-world overconsumption relative to baseline). It also highlights why caffeine habits can be a meaningful confound when ERPs are used as baseline reference measures in applied settings.

### 3.13. N2b Family and Related Selective Attention Negativities

The N2b family emerges as a mid-latency negativity that is often caffeine-sensitive, but primarily in terms of amplitude modulation rather than a consistent shift in timing. The most coherent pattern is that caffeine changes how strongly the brain expresses selection-for-action and discrimination-related processing in the N2 time range—sometimes increasing N2b amplitude when the negativity reflects active selection of relevant information, and in other contexts reducing N2b-like conflict/inhibition signals when the task demands withholding a response. Caffeine may lessen the need for extended control. This context dependence is important because “N2b” is not always functionally identical across paradigms: in perceptual selection tasks, it is often treated as an index of stimulus discrimination/selection, whereas in Go/No-Go decompositions, N2b-like activity can be intertwined with conflict and inhibitory control operations.

#### 3.13.1. N2b in Selective Attention and Stimulus Discrimination

In paradigms where N2b indexed selective attention or stimulus discrimination, caffeine generally increased the magnitude of attentional differentiation without reliably advancing onset latency. In a color-based selective attention task, an attentional N2b over centrofrontal sites (approximately 240–280 ms, maximal near Cz) showed only weak attended–unattended separation under placebo, but a clearer attention effect under caffeine. This effect was driven by more negative responses to attended non-targets and more positive responses to unattended non-targets, thereby enlarging the separation between relevant and irrelevant information at the N2b stage, while leaving onset latency unchanged [29]. A similar pattern was observed in a selective-search paradigm including both younger and older adults, in which N2b was interpreted as indexing the selection of relevant over irrelevant stimuli. Here, caffeine increased N2b amplitude in both age groups, most clearly over posterior sites, again without changing onset latency [59].

#### 3.13.2. N2b-like Negativities in Inhibition and Conflict-Related Paradigms

A different pattern emerged in paradigms emphasizing inhibitory control or conflict processing, where the functional meaning of the N2b-like negativity differed from that in selective attention tasks. In an equiprobable auditory Go/No-Go study in children, an N2b component was identified specifically on No-Go trials (peaking around 210 ms with a fronto-central, right-weighted distribution), and caffeine produced a marked increase in No-Go N2b amplitude. This effect was interpreted as enhanced processing related to active inhibitory control rather than early sensory analysis [52].

By contrast, in other Go/No-Go studies that more explicitly separated overlapping processes, caffeine-related changes in a composite P2/N2b complex on No-Go trials tended to reduce both the frontal negative N2b-like component and the accompanying central positivity [55]. These effects were interpreted as reflecting reduced conflict or inhibitory demand when withholding a response under caffeine. Thus, in inhibition-focused paradigms, caffeine did not produce a uniform directional effect on N2b-like activity. Instead, the direction of change depended on whether the negativity was interpreted as reflecting strengthened control operations or reduced conflict-related processing demands.

#### 3.13.3. Latency Effects Within the Broader N2 Window

Although the dominant N2b family pattern was amplitude modulation, there was limited evidence for caffeine-related timing effects within the broader N2 range. In a choice reaction time paradigm, where stimulus-selection demands were greater than in simple reaction time, caffeine shortened N200 latency, particularly over right-hemisphere sites, even when P300 latency did not change reliably [81]. This suggests that, in some choice settings, caffeine may accelerate relatively early selection-related processing. However, such latency effects were not the predominant finding across the N2b literature and appear better characterized as task-specific than general.

### 3.14. Processing Negativity (PN)

Across the studies summarized here, PN appears as a mid-latency, sustained negative-going activity that follows early sensory components and is typically interpreted as an index of selective processing and stimulus categorization—often larger when stimuli are task-relevant and require active selection. The caffeine-related findings for PN are notable less for a single uniform direction of change than for a topographically and condition-specific redistribution of negativity across Go versus No-Go contexts. Rather than simply “scaling” PN up or down everywhere, caffeine tends to alter where and under which trial demands PN-like activity is expressed, consistent with a shift in how attentional effort is allocated across networks and across time.

Evidence for this nuanced pattern is reported most clearly in equiprobable auditory Go/No-Go paradigms, where PN is typically larger for Go stimuli than for No-Go stimuli because Go tones require active selection and response preparation, whereas No-Go tones are behaviorally suppressed. In the child equiprobable auditory Go/No-Go study, PN was treated as a predominantly temporal negativity peaking at 145–200 ms, linked to categorization and active processing. Caffeine did not simply increase PN uniformly. For Go stimuli, caffeine enhanced the defining temporal negativity of PN. At the same time, some frontal/midline regions showed relative reductions, a configuration consistent with caffeine strengthening the PN element most characteristic of the component (categorization-related activity in temporal regions) rather than producing a global arousal-driven amplitude increase. For No-Go stimuli, the pattern diverged: PN became globally less negative under caffeine, but the key temporal negativity that defines PN was largely unchanged, implying that caffeine did not boost PN’s core categorization signal during inhibition trials and instead reduced more diffuse or nonessential negativity outside PN’s primary temporal generators [52]. This combination of effects supports the interpretation that caffeine can modulate PN in a functionally selective way—enhancing the central categorization-related expression of PN for response-relevant stimuli while trimming broader, less specific negativity when withholding is required.

Adult Go/No-Go findings are compatible with this stage- and topography-selective view, while also emphasizing that PN can shift depending on whether analysis treats the ERP as a single waveform or separates overlapping processes. In one analysis of an equiprobable auditory Go/No-Go task, caffeine produced a slight enhancement of PN to Go stimuli, particularly over frontal and hemispheric regions, aligning with faster reaction times and fewer omission errors and suggesting increased allocation of attention to response-relevant sounds [54]. However, when ERPs were decomposed into more sharply separated latent components in a closely related design, caffeine’s apparent PN effects looked more redistributive than amplificatory. For Go trials, frontal midline PN was reduced under caffeine even as earlier discrimination-related negativity (e.g., N1-1) increased, consistent with the idea that caffeine may front-load processing into earlier stages of discrimination, thereby reducing the need for sustained selective attention later in the PN interval [55]. For No-Go trials, the pattern flipped in a theoretically informative way: caffeine enhanced PN, as evidenced by increased frontal midline negativity, suggesting stronger attentional engagement or monitoring when withholding a response, while simultaneously reducing central negativity, again implying a reweighting of attentional control rather than a uniform gain increase [55].

### 3.15. Late Slow-Wave Activity After Inhibition (Late Positivity/Late Negativity/Slow-Wave)

Late activity beyond the canonical N2/P3 interval—variously labeled late positivity (LP), late negativity (LN), slow-wave (SW), or negative slow-wave (NegSW)—shows comparatively inconsistent caffeine sensitivity across the included studies. These components typically extend from several hundred milliseconds after stimulus onset (often >500 ms) and are commonly interpreted as reflecting sustained control, post-decisional evaluation, maintenance of an inhibitory set, or disengagement/closure following successful action or inhibition. Across Go/No-Go paradigms, caffeine’s most reliable effects are more often reported earlier (e.g., N2 family and P3 family activity). In contrast, very late slow-wave activity is either weakly modulated, inconsistently quantified, or becomes clearer only when analyses explicitly separate overlapping late processes.

In the child equiprobable auditory Go/No-Go study, very late components were explicitly identified and quantified. An LN in the Go condition and an LP in the No-Go condition were observed after ~700 ms. For Go trials, LN showed only weak, spatially restricted caffeine effects that did not reach statistical reliability. For No-Go trials, LP likewise showed only small, nonsignificant increases, limited in topography. A related late frontal slow negativity after No-Go stimuli (NegSW) showed only a trend-level shift toward increased frontal negativity under caffeine without reaching conventional significance, reinforcing the impression that extended slow activity is a comparatively variable locus of caffeine effects in conventional waveform analyses [52].

Adult Go/No-Go findings suggest that late slow-wave components can show clearer caffeine modulation in some contexts, particularly on No-Go trials, and especially when analyses isolate late subcomponents. In one equiprobable auditory Go/No-Go dataset, caffeine produced relatively little change in late positivity overall, with the more consistent post-P3 effect being a modest enhancement of a No-Go slow-wave component in central hemispheric regions [54]. In a more detailed component-separation analysis of a closely related design, caffeine-related modulation of late activity was more apparent: caffeine enhanced No-Go slow-wave activity (including increased central positivity and a later pattern combining increased frontal negativity with increased central positivity). Also, it increased No-Go late positivity more globally across the scalp, interpreted as strengthened sustained inhibitory processing and/or a more pronounced “wrap-up” phase following successful inhibition [55].

### 3.16. Temporal T-Complex

While much of the auditory caffeine literature emphasizes vertex N1-P2 amplitude/latency, one study highlights a subtler target: the temporal T-complex (Ta/Tb) associated with secondary auditory regions and interhemispheric timing relationships. Here, caffeine did not strongly alter vertex N1 or P2 latencies, and it mainly prevented placebo-like habituation of N1-P2 amplitude. But caffeine did shorten the Ta latency of the T-complex (small but significant), indicating a measurable acceleration in secondary auditory cortical timing that was not obvious at the vertex [65]. Even more distinctive was the finding on ipsilateral–contralateral Ta latency differences during monaural stimulation: placebo showed no systematic change, whereas caffeine increased this asymmetry in a subset of participants (and ethanol did so more robustly). This points to a caffeine-sensitive dimension that standard midline ERP summaries can miss—lateralized timing relationships rather than single-site peak latencies [65].

### 3.17. Auditory P1

Evidence for a caffeine effect on the auditory P1 component is minimal and largely indirect. The clearest mention of an auditory P1 comes from the equiprobable auditory Go/No-Go study in children ([52]), where P1 was identified as an early sensory component peaking very rapidly after tone onset (roughly 70 ms) and showing a frontal distribution. In that dataset, caffeine (80 mg) produced only small, localized changes in the Go-evoked P1 that did not amount to a broad, uniform amplification or a clear latency shift, suggesting that caffeine did not robustly alter the earliest stage of auditory sensory encoding. The same study reported that, in the No-Go condition, early sensory-stage effects of caffeine were also limited at the P1 stage, with the more reliable caffeine-related modulations emerging later (e.g., in later N2 and P3 family components associated with categorization and control). A complementary adult Go/No-Go auditory study [32] similarly treated P1 as an early sensory response (around ~40 ms in that paradigm) and described it as topographically similar for Go and No-Go tones, with caffeine producing no statistically reliable changes in the early sensory components; again, caffeine effects were described as absent or negligible at the earliest auditory stage relative to later positive components linked to response execution. Taken together, these findings converge on a consistent interpretation: while caffeine often modulates later ERP activity tied to attention, decision-making, and control, its impact on auditory P1—when measured in simple auditory discrimination or Go/No-Go contexts—appears weak, inconsistent, and not clearly systematic, implying that caffeine’s electrophysiological action is more evident after initial sensory registration rather than at the earliest auditory cortical response.

### 3.18. Early Visual P1 and C1P1-Range Activity

A further ERP parameter used to test whether caffeine boosts the very earliest stages of sensory encoding is the early visual positivity in the C1/P1 range (roughly 80–140 ms), often interpreted as reflecting initial feedforward processing in primary and early extrastriate visual cortex. Across these studies, caffeine’s influence appears weaker and less consistent than its effects on later attentional and evaluative components, though it can emerge under certain formulations and tasks. In the energizing-drink experiment that combined a modest caffeine dose with glucose, the active drink produced more positive-going activity in the earliest visual interval (around 80–100 ms), with the effect strongest over electrodes contralateral to the stimulated visual field, consistent with an enhancement of early sensory responses in the visual cortex rather than a purely late, decision-related shift [44]. By contrast, when caffeine was tested in paradigms where early spatial-attention effects on P1 are robust, caffeine itself did not reliably amplify that early sensory gain: in a spatial-selective attention task, the classic attention-related P1 enhancement was present, but caffeine showed no clear main effect on occipital P1 and no latency changes, implying that early visual sensory gain mechanisms can remain intact yet essentially unchanged by caffeine [51]. A similar boundary case appears in simultaneous EEG-fMRI work, where caffeine altered vascular signals strongly but left visual evoked potentials—defined in an early peak-to-peak window over occipital electrodes—statistically unchanged in both amplitude and latency, reinforcing the idea that caffeine does not necessarily boost the earliest visual evoked response even when it measurably shifts other neural indices in more cognitive tasks [38].

### 3.19. Error-Related Negativity and Error Positivity

A distinct ERP parameter that caffeine modulates, separate from stimulus-locked attention and evaluation, is the family of error-related components observed after an incorrect response, most prominently the error-related negativity (ERN) and the error positivity (Pe). These response-locked signals are typically interpreted as indices of action monitoring, with the ERN reflecting rapid, largely automatic detection that something has gone wrong, and the Pe reflecting subsequent evaluative or awareness-related processing of the error. In the task-switching study that directly targeted action monitoring, caffeine produced a clear, selective strengthening of ERN: both a low dose (3 mg/kg) and a higher dose (5 mg/kg) increased ERN amplitude relative to placebo. The same pattern held for the Pe, with caffeine yielding a larger post-error positivity in the 200–400 ms range [33]. What makes this finding especially informative is that the ERN/Pe enhancement did not appear to be a trivial byproduct of improved performance; when the authors statistically controlled for caffeine-related differences in the error rate, the amplitude increases remained significant, suggesting that changes in error effects did not fully explain caffeine’s effects and may have involved altered action monitoring processes [33]. Equally importantly, the effect was specific: stimulus-locked components measured on correct trials (including P2, N2, and P3 in that paradigm) did not show comparable caffeine-related changes, arguing against a simple global amplification account and suggesting instead a more selective effect on processes associated with action monitoring [33]. In this sense, caffeine’s impact on ERN/Pe complements the broader literature on attention-related components by suggesting that caffeine may influence not only stimulus processing but also performance monitoring processes, increasing the salience or neural “gain” of error detection and subsequent error evaluation, even when other ERP markers of perceptual and evaluative processing remain unchanged.

### 3.20. Feature-Selection Components: Frontal Selection Positivity and Occipital Selection Negativity

A useful ERP parameter for separating “general arousal” effects from genuinely selective attention effects is the pair of early feature-selection components often seen in nonspatial attention tasks: the frontal selection positivity (FSP) and the occipital selection negativity (SN). These components typically emerge in the ~150–200 ms range when participants selectively attend to a feature (such as color), and they are taken to reflect early top-down biasing of sensory processing—frontal engagement for selection (FSP) alongside enhanced feature processing in visual cortex (SN). In the color-based selective attention study, both FSP and SN were robust under placebo and remained robust after caffeine, showing the expected differentiation between attended and unattended colors, but their amplitudes were not significantly altered by caffeine; the attentional selection mechanism itself looked intact and essentially unchanged in strength at this early stage despite caffeine speeding reaction times and producing clear modulation of other ERP components later in the stream [29]. What makes this pattern informative is that caffeine’s electrophysiological impact in the same dataset was not absent—it showed up elsewhere (for example, in stronger attentional differentiation at the N2b stage and increased early P2 positivity)—but the classic early selection markers that index feature-based filtering were comparatively resistant.

### 3.21. Lateralized Readiness Potential Onset (LRP)

A further ERP-derived parameter used to localize caffeine’s effects within the perception–action chain is the LRP, especially its stimulus-locked onset latency, which marks the onset of response preparation lateralization toward the responding hand. In a visual selective attention flanker-style task that separated stimulus evaluation from motor preparation, caffeine (3 mg/kg) did not noticeably reshape the stimulus-locked ERP morphology in the classic P3 range. Still, it did produce a clear shift in motor preparation timing: the LRP began earlier after caffeine than after placebo, indicating that information about the relevant target was transmitted sooner to the motor system and that central response preparation was initiated earlier, particularly in conditions with competing flanker information where preparation is normally delayed [34]. This kind of effect supports an account in which caffeine can shorten the time required to progress from perceptual identification to activating the correct response hand, even when the timing of later stimulus evaluation markers (such as P3 peak latency) remains unchanged. However, LRP findings also show that caffeine does not universally speed motor preparation. In another visual selective attention paradigm, caffeine shortened P3 latency for targets and task-relevant stimuli but left LRP onset latency identical under caffeine and placebo, implying that caffeine’s behavioral speeding (when present) could arise from earlier evaluative stages or later execution-related processes rather than from an earlier start of motor preparation per se [49].

### 3.22. Movement-Related Cortical Potentials and Readiness Potentials

Caffeine’s effects are not limited to stimulus-locked cognition; they also appear in movement-related cortical potentials (MRCPs), including the readiness potential that builds up before voluntary movement and the motor-related negativity expressed around movement execution. In sustained force production, caffeine can reduce the cortical motor drive needed to produce the same physical output. During repeated submaximal knee extensions, caffeine (6 mg/kg) reliably attenuated MRCP amplitude during the contraction epochs, with the signal at Cz becoming less negative under caffeine than placebo even though torque and EMG remained stable, implying that the brain was generating equivalent force with reduced motor-cortical activation; this attenuation tracked subjective experience as well, because more negative MRCPs were associated with higher perceived effort, and caffeine’s reduction in MRCP negativity aligned with lower effort ratings [47]. In contrast, when the question is whether a small, everyday dose meaningfully reshapes MRCP morphology during discrete movements, effects can be minimal: after a cup of coffee (~85 mg caffeine) during repeated ankle dorsiflexions, MRCP component amplitudes (readiness potential, negative slope, motor potential) showed no consistent caffeine-related changes beyond normal within-session variability, even though classification accuracy for detecting MRCPs improved slightly in the post-coffee session, suggesting that caffeine may not strongly alter the average waveform but can still nudge signal detectability in applied contexts [39]. Under more complex physiological stress, caffeine can also alter premovement dynamics, suggesting a change in the motor system’s ramp-up efficiency. In an exercise protocol manipulating workload, the readiness potential (Bereitschaftspotential) normally expanded and grew more negative with rising exertion under placebo, including greater frontal recruitment at maximal effort; with caffeine alone, premovement activity showed earlier or stronger engagement of central/parietal motor regions at lower workloads and less frontal expansion under exhaustion, consistent with a more focused motor preparation pattern that may reduce the need for additional frontal “boost” when fatigued [40].

### 3.23. N2 Latency

N2 latency is generally not a robust locus of caffeine effects, with most paradigms reporting either no change or, at most, modest nonsignificant trends. In the adult visual Go/No-Go study conducted under 36 h of total sleep deprivation ([25]), however, caffeine produced a clear, context-specific latency effect. During No-Go trials, caffeine was associated with shorter N2 latencies than placebo after sleep deprivation, especially over frontal regions. This pattern is consistent with caffeine speeding up the timing of processes involved in conflict detection and the initiation of inhibitory control when participants are severely fatigued, even though caffeine did not broadly normalize later P3 timing in the same study.

Outside of that fatigue-challenged setting, N2 latency effects were largely absent. In the auditory Go/No-Go task in young adults [32], caffeine produced no statistically reliable changes in N2 latency (for either Go or No-Go), suggesting that early-to-mid control operations indexed by N2 were not accelerated in that simpler auditory inhibition context. Similarly, in an auditory oddball study that measured N1/P2/N2/P3 latencies before and after caffeine [37], N2 latency tended to shorten across sites. Still, these reductions were not statistically significant, implying that any caffeine-related speeding at the N2 stage was weaker than later amplitude effects (notably on P3). Finally, in the spatial-selective attention study [51], caffeine was reported not to alter component latencies overall, consistent with a primarily amplitude- and interaction-based influence rather than a timing shift at N2.

### 3.24. N2c

Findings on N2c are clearest from the equiprobable auditory Go/No-Go study in children [52], where N2c was treated as a later negative component associated with response-related processing rather than simple sensory encoding. In the Go condition, N2c peaked at roughly ~260 ms and showed a fronto-central midline maximum, consistent with involvement of systems supporting response preparation and stimulus-to-response translation. Caffeine (80 mg) produced a robust amplitude enhancement of this Go-related N2c, which was broadly distributed across the scalp, with especially pronounced increases over frontal and lateral regions. This pattern aligns with the interpretation that caffeine strengthens the engagement of preparatory control processes once a response is being organized. A related but importantly different effect was reported for No-Go trials in the same study, where a frontal N2c variant (fN2c) was identified later (around ~260–280 ms) with a frontal distribution. Here, caffeine did not increase the component; instead, fN2c amplitude was significantly reduced, and the reduction was characterized as global and strongest over fronto-central/midline areas.

### 3.25. N140-P200 Complex Amplitude

An additional ERP parameter that caffeine can modulate—sometimes in the opposite direction to the “enhancement” pattern seen for later cognitive components—is the amplitude of early visual evoked complexes, such as the N140-P200. In children diagnosed as hyperkinetic, caffeine produced a clear, dose-related attenuation of the N140-P200 evoked response amplitude, most reliably at occipital sites: as caffeine dose increased (placebo → ~3 mg/kg → ~6 mg/kg), the evoked response became smaller, with particularly strong reductions at O2 (highly significant) and a weaker but still significant reduction at O1, alongside a more limited right-parietal effect at P4 [48]. Notably, these amplitude changes occurred without convincing latency shifts, indicating that caffeine primarily reduced the magnitude of early visual cortical responsiveness rather than consistently speeding or delaying its timing [48]. This pattern is informative because it shows that caffeine’s electrophysiological impact is not uniformly “upward”—early sensory-evoked activity can be damped under some conditions and populations. It also contrasts with adult findings in which caffeine left early visual evoked potentials essentially unchanged while altering later cognitive markers in other tasks, suggesting that caffeine’s effects on early sensory ERPs may depend strongly on developmental state, baseline neurophysiology, and the specific evoked component being measured [38].

### 3.26. N170 and N250 Negative-Bias Indices in Face Processing

Another ERP parameter that helps map the limits of caffeine’s acute influence is the set of early-to-mid-latency face-sensitive components, especially the N170 (often linked to structural encoding of faces) and the later N250 (often tied to more individuated or affect-related face processing). In the older-adult coffee study that included an Emotional Face Recognition Task, these components were quantified at posterior-occipito-temporal sites (PO7/PO8) and summarized as “negative-bias” ERP indices (sad minus happy peak amplitude) to test whether acute coffee manipulations would shift emotional processing at the level of early face-perceptual signals. Despite caffeinated coffee producing clear subjective alertness changes and some cognitive benefits elsewhere in the battery, neither the N170 nor the N250 negative-bias measures showed a reliable caffeine-related modulation, and even the high-chlorogenic-acid decaf condition, while reducing behavioral negative-bias in accuracy, did not produce a corresponding shift in the N170/N250 bias indices [46]. In practical terms, this pattern suggests that acute caffeine (in this protocol and age group) did not measurably alter the early electrophysiological signatures of face encoding or the sad-versus-happy differentiation captured by these ERP peaks, even when mood and attention measures were affected, reinforcing the broader theme that caffeine’s most dependable ERP effects tend to appear in components tied to task engagement and evaluative control rather than in early category-specific perceptual markers.

### 3.27. P3a and Novelty-Related P3 Activity

An ERP parameter that has been probed for caffeine sensitivity is the more “orienting” branch of the P3 family—often labeled P3a (and closely related novelty-P3 activity)—which is typically fronto-centrally distributed and linked to involuntary attention switching toward salient events, particularly in tasks that include No-Go signals or novel/distractor stimuli. Across studies, caffeine’s influence on this parameter appears mixed, suggesting that it does not reliably amplify orienting responses and may even dampen them under some conditions. In the auditory Go/No-Go study in young adults, No-Go stimuli elicited a centrally maximal P3a-like response. Yet, caffeine produced no statistically reliable changes in No-Go P3a amplitude or latency, indicating that the neural signature of attentional orienting to inhibitory signals was essentially stable under caffeine in that paradigm [32]. In children performing an equiprobable auditory Go/No-Go task, caffeine again did not act as a uniform booster of late activity; instead, while it enhanced some control-related negativities (notably No-Go N2b), it reduced the No-Go P3a in central and left-hemisphere regions, a pattern the authors interpreted as consistent with less need for orienting or attentional switching once earlier categorization and inhibition processes were more efficiently engaged under caffeine [52]. Converging “null” evidence comes from the simultaneous EEG-fMRI study using an auditory oddball with multiple stimulus classes: caffeine robustly shortened target P300 latency but did not significantly affect the novelty-P300 (nor the P200 to non-targets), suggesting that caffeine’s most reliable timing effects in that dataset were tied to task-relevant target evaluation rather than to novelty-driven orienting responses [38].

### 3.28. P450 Amplitude

Another ERP parameter reported as caffeine-sensitive in applied, multi-ingredient interventions is the P450 amplitude, a later positive component often discussed as reflecting sustained attention and working-memory updating during ongoing task engagement. Because the original study labeled this effect as P450 and because the task context and topography do not permit confident classification as P3a or P3b, we treat it here as a distinct late positive activity rather than merging it with P3 family findings. In the functional-beverage study that combined caffeine with citicoline, participants who consumed the active drink showed reliably higher P450 amplitudes than those who received placebo across a broad set of electrodes spanning prefrontal, frontal, fronto-central, central, and parietal regions, with the strongest and most consistent increases concentrated in frontal and prefrontal sites (including F3/Fz/F4 and FC3/FCz/FC4, extending through central and centro-parietal midline locations such as Cz and CPz) [45]. The topography of this enhancement is important because it aligns the effect with networks that support sustained cognitive control and active maintenance of task goals, rather than with a narrowly sensory change at posterior sites. At the same time, the interpretation has an inherent limitation. Because the drink contained both citicoline and caffeine, the P450 amplification cannot be cleanly attributed to caffeine alone, and the most defensible conclusion is that a caffeine-containing intervention can coincide with a distributed increase in late positive activity during cognitive testing that resembles an upshift in attentional engagement and working-memory-related recruitment [45].

### 3.29. Parietal NP1

Only one of the studies explicitly reported a caffeine effect on a parietal NP1 measure, and that effect was subtle and qualified rather than a straightforward “increase” or “decrease.” In the spatial-selective attention experiment [51], the authors examined very early visual ERP activity and identified an NP1-like deflection in the parietal time range of roughly 90–110 ms. Caffeine did not produce a clear, uniform change in NP1 latency or a robust main effect on NP1 amplitude across the scalp. Instead, the reported effect was an interaction between caffeine and hemisphere: caffeine’s influence on parietal NP1 activity was stronger over the left parietal cortex than the right. Importantly, the authors interpreted this cautiously because the hemisphere interaction did not translate into a clear, easily localized effect at individual electrode sites and did not coincide with broader caffeine-related changes in the classic early occipital attention components within that task. Functionally, this pattern suggests that, if caffeine modulates NP1 at all in this paradigm, it may do so in a lateralized and regionally specific manner—consistent with the broader theme across ERP caffeine studies that early sensory-stage effects are often modest and context-dependent—rather than reflecting a general amplification of early perceptual encoding.

### 3.30. Peak-to-Peak Visual Evoked Potential Amplitude

Another ERP parameter that has been used to test caffeine’s influence on early sensory encoding—while also revealing an important dissociation between vascular and neural measures—is the peak-to-peak amplitude of the early visual evoked potential (VEP). In the simultaneous EEG-fMRI study, the VEP amplitude was defined as the peak-to-peak difference between an early negative deflection and a subsequent positive deflection recorded at occipital electrodes (O1, Oz, O2), reflecting the strength of the earliest stimulus-driven visual response. Despite caffeine producing a pronounced reduction in cerebral blood flow and attenuating BOLD responses in the visual cortex during the checkerboard task, the electrophysiological VEP remained unchanged in both amplitude and latency, indicating preserved early visual neural responsiveness even when hemodynamic signals were substantially altered [38]. This stability of the peak-to-peak VEP parameter is consequential because it suggests that, at least for low-level visual stimulation, caffeine does not reliably increase or decrease the magnitude of the earliest evoked cortical response; instead, caffeine’s measurable neural effects may be more likely to appear in later, task-dependent components, while early sensory ERPs can remain largely invariant even under substantial systemic physiological shifts.

### 3.31. Early Sensory P1-N1 and N1-P2 Amplitude Measures During Withdrawal and Resumption

An ERP parameter that has been used to probe caffeine’s influence—especially in habitual users—is the early peak-to-peak amplitude of sensory complexes, particularly P1-N1 and N1-P2 in auditory ERPs. In a controlled withdrawal–reintroduction design, caffeine withdrawal itself produced a strong subjective syndrome (fatigue, lethargy, headache in many participants), yet ERP timing measures were largely stable, and overt task performance showed little consistent impairment. Against that backdrop of relatively preserved latencies, the early sensory amplitude measures emerged as the more sensitive ERP link to caffeine status. Still, they did so in a very specific way: rather than showing a uniform main effect of withdrawal or reintroduction across the whole sample, changes in P1-N1 and N1-P2 amplitudes during the resumption phase correlated with individuals’ habitual caffeine intake. Participants who typically consumed more caffeine tended to show larger amplitude shifts in these early components when caffeine was reintroduced after abstinence, suggesting that regular exposure may “tune” early cortical responsiveness, so that reinstating caffeine produces a larger change in the gain of sensory responding. Importantly, these amplitude relationships were not strongly explained by pharmacokinetic differences (such as caffeine half-life), suggesting that the relevant driver was habitual consumption level and its neuroadaptive consequences rather than simple differences in acute caffeine availability over time. The overall pattern, therefore, positions early sensory amplitude complexes as a subtle but informative parameter: they may not show dramatic group-average changes with short-term withdrawal, yet they can reveal individual differences in how strongly the auditory cortex’s early response magnitude rebounds with caffeine, even when later cognitive ERP timing and reaction time remain relatively unaffected [60].

### 3.32. Late Positive Complex During Stroop Stimulus Processing

A further ERP parameter that highlights how caffeine can shape later-stage cognitive processing is the late positive complex (LPC) elicited during Stroop stimulus evaluation, typically expressed as a parietally distributed positivity well after early conflict signals. In the reward-cued Stroop paradigm, incongruent stimuli produced the expected conflict-related negativity in the mid-latency range. Still, this earlier interference marker was essentially unchanged by caffeine, suggesting that caffeine did not substantially alter initial conflict detection. The clearer caffeine effect emerged later, in the LPC window over parietal regions, where activity is often taken to reflect sustained stimulus evaluation, task-relevant updating, or extended control-related processing needed to implement the correct response. Reward prospect increased LPC amplitude on its own, consistent with deeper processing when performance has motivational value, and caffeine produced a parallel enhancement: LPC amplitudes were generally larger after caffeine than after placebo. Critically, caffeine did not merely add a constant offset; it strengthened the reward-related LPC enhancement, yielding the largest late positivity when participants had consumed caffeine and were also expecting a potential reward. This pattern links caffeine to a selective amplification of late-stage, task-relevant processing under motivational demand, implying that caffeine can increase the depth or stability of stimulus-response resolution in the later processing stream, even when earlier conflict-related ERP signatures remain relatively unaffected [73].

### 3.33. N1-P2 Complex and Long-Latency Auditory-Evoked Amplitudes

Caffeine also modulates long-latency auditory-evoked activity in the N1-P2 range. However, the direction of change depends strongly on context and what the auditory stream “means” relative to the participant’s task goals. When auditory clicks are presented while attention is primarily engaged elsewhere, caffeine can appear to suppress certain attention-sensitive amplitude metrics, consistent with a tighter focus on the primary task rather than enhanced processing of irrelevant sound. During a two-hour visual vigilance task accompanied by continuous auditory clicks, caffeine did not reliably alter N1 or P2 latencies compared with placebo. Still, it reduced the P2-N2 amplitude relative to placebo throughout the session. Because P2-N2 amplitude in this paradigm was interpreted as reflecting the degree of attentional allocation to the auditory stimulus, the caffeine-related reduction suggests that participants under caffeine maintained their attention on the demanding visual task and were less drawn into processing the irrelevant auditory stream, even as time-on-task produced a general decline in amplitudes across all conditions [64]. A compatible pattern emerges from work that explicitly tracked habituation: after placebo, vertex N1-P2 amplitude declined from baseline (a typical habituation/fatigue effect), whereas after caffeine, the vertex N1-P2 complex was largely preserved near baseline levels. In other words, caffeine did not necessarily “boost” N1-P2 above baseline. Still, it counteracted the amplitude drop observed without stimulant support, suggesting a stabilizing effect on cortical responsiveness when repeated stimulation would otherwise dampen the response [65].

At the same time, caffeine’s influence on N1-P2 amplitude can reverse in settings where the manipulation is not “auditory distraction during a visual task” but rather a direct probe of sensory reactivity or clinical vulnerability. In an anxiogenic challenge study comparing generalized anxiety disorder, panic disorder, and healthy controls, caffeine produced broader dose-related changes in auditory-evoked responses, and the GAD group showed exaggerated ERP reactivity. One of the clearest group differences was a larger caffeine-related reduction in N1-P2 amplitude in GAD compared with controls, indicating that, in a population characterized by heightened anxiety and arousal sensitivity, caffeine can drive stronger modulation of early auditory cortical responsiveness rather than merely stabilizing it [73]. Even in withdrawal-focused work, where overall ERP timing was relatively preserved, early amplitude measures (P1-N1 and N1-P2) showed relationships with habitual intake during caffeine resumption, suggesting that these early auditory amplitudes can be sensitive to caffeine history even when overt performance remains stable [60]. Taken together, the N1-P2 family illustrates a useful theme across the caffeine–ERP literature: caffeine does not exert a single monotonic “increase” on early auditory amplitudes, but instead can either preserve, reduce, or disproportionately alter N1-P2 range measures depending on whether the auditory input is task-relevant, a competing distractor, or a probe of heightened arousal states.

### 3.34. N400

Most caffeine–ERP work targets attention (P3) or early sensory components (N1/P50), but the MTB “brain vital signs” protocol included N400, indexing semantic processing. In that cross-sectional baseline setting, caffeine presence in the prior 24 h (yes/no) did not meaningfully relate to ERP amplitudes or latencies overall. The more informative result was conditional: athletes reporting more caffeine than usual showed an N100 that was smaller and earlier, but other components, including P300 and N400, were not clearly associated with caffeine deviation in the reported models [78].

### 3.35. N450

In contrast to acute dosing experiments, one study examined whether habitual daily caffeine intake predicts stronger neural markers of inhibitory control during the Stroop task. The answer was largely negative. The classic N450 interference component did not show a significant congruency effect in their data, and neither its overall amplitude nor its congruency contrast was reliably related to habitual caffeine (mg/day). A later posterior positivity (600–900 ms) did show a significant congruency effect overall. Still, habitual caffeine did not significantly modulate the effect size again (the interaction trended but did not meet the study’s threshold) [77]. This provides an important counterpoint to acute ERP enhancements: “more caffeine in daily life” does not straightforwardly translate into larger conflict-related ERP signatures in a cross-sectional design, at least for these Stroop indices and this sample.

### 3.36. N500 Difference Wave Area as a Marker of Extended Working-Memory Processing

A further ERP parameter that highlights caffeine’s more selective and context-dependent influence is the N500 difference wave—a late negative-going activity (often quantified by its area over a broad post-stimulus window) that has been interpreted as reflecting extended, working-memory-related processing demands, particularly in tasks that require choosing among alternatives rather than making a simple response. In a design that compared placebo, alcohol, caffeine, and their combination within the same participants, the N500 difference wave was most clearly expressed in the choice reaction time task and showed a broad centro-parietal distribution, consistent with the idea that it indexes sustained processing beyond early selection and P300-stage evaluation. Caffeine on its own did not reliably change N500 peak amplitude or latency, suggesting that, at a moderate dose, it does not necessarily increase or accelerate this extended processing stage when participants are otherwise unimpaired. However, caffeine did modulate the extent of N500 activity in a very specific circumstance: when co-administered with alcohol, caffeine significantly reduced the overall area of the N500 difference wave (measured across a large 220–900 ms window), especially at frontal and left-hemisphere sites. This pattern implies that caffeine can alter late, sustained processing not by enhancing it universally, but by reshaping how much extended working-memory engagement is recruited under combined pharmacological states—here, effectively compressing or attenuating the prolonged negative activity that normally accompanies complex response selection when alcohol is present. In other words, the N500 parameter shows that caffeine’s ERP signature is not always a straightforward “boost” to cognitive processing; instead, it can be most visible as a modulation of late, sustained processing demands when the broader neurocognitive state is perturbed, revealing interactions at the level of extended processing dynamics rather than early sensory encoding or simple stimulus evaluation alone [81].

### 3.37. Search-Related Sustained Negativity During Visual Search

An ERP parameter that helps localize caffeine’s effects is the search-related sustained negativity observed during visual search, a negative-going activity that becomes more pronounced when participants must engage in controlled, often serial, scanning of multiple items and reject non-targets. In the visual search paradigm that manipulated focused versus divided attention and varied display load, this sustained negativity behaved exactly as a marker of search demand should: it increased as more items had to be checked, reflecting the added need for controlled inspection when the display became more complex. Crucially, caffeine did not reliably change either the amplitude or the timing of this search-related negativity. Even though participants responded faster under caffeine, the electrophysiological signature of controlled search—the sustained negative activity tied to rejecting non-targets and stepping through candidate items—remained essentially unchanged. This dissociation is informative because it suggests that caffeine’s performance benefits in visual search were not achieved by making the core search operation itself more efficient or by strengthening the neural mechanisms of early selective filtering; instead, caffeine’s influence emerged later, at the level of stimulus evaluation indexed by P3b latency, while the sustained negativity continued to scale with display load in the same way under caffeine and placebo. In other words, the search-negativity parameter provides a kind of “negative evidence” that is theoretically useful: caffeine can speed responses and accelerate later evaluation without measurably altering the electrophysiological footprint of controlled search operations, implying that the limiting step in this task remains unaltered. Still, the downstream categorization/decision processes are expedited once the search has delivered enough information to support a target judgment [61].

### 3.38. Distinguishing Caffeine-Specific Effects from Combined Effects

#### 3.38.1. Caffeine and Taurine

In a study involving movement-related cortical potentials (the readiness potential or BP), the specific influences of taurine and caffeine were clearly differentiated:

- Caffeine-Specific Effects: Caffeine alone accelerated and amplified premovement cortical activity. It resulted in a significantly earlier BP onset and an “overshoot” of BP power at central and parietal sites.
- Combined Effects (Caffeine + Taurine): The addition of taurine attenuated caffeine’s specific acceleration and amplification. This led to a more continuous, gradual increase in BP magnitude and a more efficient, spatially restricted motor preparation pattern compared with caffeine alone.

#### 3.38.2. Caffeine and L-Theanine

Studies on selective attention and auditory ERPs quantified the interaction between these two substances:

- Caffeine-Specific Effects: Caffeine alone improved recognition reaction time and increased the N2–P300 peak-to-peak amplitude, a marker of neural resource allocation.
- Combined Effects: The combination produced the greatest improvement in reaction time and the largest N2–P300 amplitude.
- Distinction: Factorial analysis revealed that the combined effect was additive rather than synergistic, meaning both ingredients contributed their individual strengths without a complex interaction.

#### 3.38.3. Caffeine and Alcohol

The studies highlight how caffeine can selectively antagonize or alter the neural signatures of alcohol:

- Antagonistic Effects: Alcohol alone prolonged the latency of the MMN component (slowing automatic auditory change detection). When caffeine was co-administered, it significantly reduced this latency, returning it toward placebo levels. Caffeine alone did not affect MMN, showing its role was specific to counteracting alcohol.
- Combined Interference: In conflict monitoring tasks (N2 component), alcohol alone blunted neural responsiveness, while caffeine alone enhanced it. However, the combination of the two created an abnormal pattern of neural adaptation—specifically, a reduced N2 following high-conflict trials—that was not present in the alcohol-only or caffeine-only groups.

#### 3.38.4. Caffeine and Glucose

In studies using “energizing drinks” containing both caffeine and glucose, the sources suggest a general facilitation of processing:

- Combined Effects: Participants showed faster reaction times and increased accuracy, alongside enhanced early sensory (C1/P1) and later cognitive (N2 and P3) ERP amplitudes.
- Distinction: The researchers noted that there were no significant interactions between the drink and attentional demands, indicating the combination increased overall processing capacity and efficiency rather than selectively altering specific attentional mechanisms.

### 3.39. A Structured Comparison of Low, Moderate, and High Doses of Caffeine on ERPs Results

Based on the sources, the effects of caffeine are often nonlinear and vary with dosage, the specific neural process being measured, and the individual’s state (e.g., rested vs. fatigued). The following is a structured comparison of low, moderate, and high doses as defined and investigated across the studies. A summary of dose–response trends is presented in Table 2.

#### 3.39.1. Low Doses (Approx. 1.5–3 mg/kg or 55–80 mg)

Low doses are typically associated with targeted improvements in performance and specific neural markers without the signs of overstimulation seen at higher levels.

(a) Behavioral Performance:

Doses as low as 1.5 mg/kg significantly improve processing speed in rapid information processing tasks. In children, 80 mg reduces reaction times and decreases omission errors. A dose of 55 mg (from an energy supplement) was sufficient to reduce the subjective and neural effects of accumulating mental fatigue. However, it did not significantly alter behavioral reaction times in that study.

(b) ERP Effects:

- P300 Component: A 200 mg dose (approx. 3 mg/kg) enhances P300 amplitude following fatigue in athletes, suggesting better neural resource allocation.
- Error Processing: Both 3 mg/kg and 5 mg/kg doses significantly increase the amplitude of the error-related negativity (ERN) and error positivity (Pe), indicating enhanced automatic and conscious error monitoring.
- Motor Preparation: At 3 mg/kg, the onset of the lateralized readiness potential (LRP) is shortened, meaning information is transmitted to the motor system earlier.

#### 3.39.2. Moderate Doses (Approx. 3–5 mg/kg or 200–300 mg)

Moderate doses are the most frequently studied and generally show robust effects on both behavior and cortical arousal markers.

(a) Behavioral Performance:

Consistently reduces reaction times and improves accuracy across various tasks. Benefits are often more pronounced when task demands are high, such as when perceptual input is degraded.

(b) ERP Effects:

- Attentional Resources: Moderate doses (e.g., 200–250 mg) reliably increase P3b amplitude, reflecting heightened phasic cortical arousal and increased allocation of resources to task-relevant stimuli.
- Early Processing: Enhances the N1 amplitude, indicating improved early sensory processing and accelerated perceptual encoding.
- Selective Attention: Increases the neural contrast between relevant and irrelevant information, as seen in the enhanced N2b component.
- Sensory Gating: A 200 mg dose (but not 100 mg) significantly impairs auditory sensory gating, as evidenced by an increased P50 ratio.

#### 3.39.3. High Doses (Approx. 5–6 mg/kg or 400–500 mg)

High doses can elicit maximal neural responses but may also lead to diminishing returns or overstimulation.

(a) Behavioral Performance:

At 6.0 mg/kg, the benefits to processing speed observed at lower doses may disappear, with signs of overstimulation such as increased performance errors and reduced efficiency. However, other studies at this dose still show significantly faster reaction times mediated by earlier stimulus evaluation.

(b) ERP Effects:

- Processing Speed: A 400 mg dose significantly reduces P300 latency at the frontal site (Fz), indicating enhanced neural processing speed in regions linked to executive function.
- Amplitude Modulation: A very high dose of 500 mg can increase P300 peak amplitude to 150% of baseline during an oddball task.
- Inhibitory Control: In children, a high dose (6 mg/kg) causes a dose-related reduction in the N140–P200 complex amplitude, suggesting attenuated cortical responses to visual stimuli.
- Motor Activity: At 6 mg/kg, caffeine significantly attenuates MRCP amplitude during muscle contractions, suggesting that less cortical motor drive is required to generate the same force.

### 3.40. Risk of Bias Assessment

The bias risk assessment is presented in Table 3 (Rob-2) and Table 4 (Rob-1).

Analysis of the included studies reveals several potential risks of bias that could affect the validity and generalizability of the findings regarding caffeine’s effects on the brain.

#### 3.40.1. Selection and Performance Bias

The vast majority of the analyzed studies utilized randomized, double-blind, placebo-controlled crossover designs, which significantly reduces selection bias and accounts for inter-individual variability. This design ensures that each participant acts as their own control, a critical feature in ERP research where baseline neural activity varies widely. To enhance performance blinding, several researchers employed sophisticated measures such as disguising the taste and smell of the treatment using decaffeinated coffee or deceptive information protocols where participants were led to believe they were consuming caffeinated beverages regardless of the actual content.

However, some studies utilized single-blind designs, which increases the risk of performance bias if experimenters inadvertently influenced participant responses. Furthermore, a few studies employed open, within-subject designs, which are susceptible to expectancy effects. A notable exception to the crossover trend was the use of between-subject designs in specific contexts, such as evaluating energy drinks or citicoline combinations, which introduces the risk of confounding variables despite randomization.

#### 3.40.2. Procedural Controls and Reporting Bias

A primary strength across the sources is the implementation of strict caffeine and alcohol abstinence periods, typically ranging from 4 to 24 h prior to testing to ensure a clear baseline. The risk of reporting bias regarding compliance was mitigated in several high-quality studies through biochemical verification via saliva samples. Conversely, studies relying solely on self-reported abstinence carry a higher risk of “hidden” caffeine presence in the baseline measurements. Washout periods between sessions generally varied from one week to one month, which is pharmacologically sufficient to prevent carryover effects, though shorter periods of 48 h or 2 to 4 days may pose a residual risk in slow metabolizers.

#### 3.40.3. Small Sample Sizes and Specific Populations

A significant number of the studies utilized very small sample sizes, often ranging from 9 to 16 participants. These small cohorts increase the risk of Type II errors (failing to detect a real effect) and make the findings susceptible to the influence of outliers.

Furthermore, many studies focused on highly specific demographics, which limits the generalizability of the results to the broader population:

- Athletes: One study focused exclusively on competitive taekwondo athletes.
- Children: Two studies examined children, with one specifically looking at those diagnosed as “hyperkinetic”.
- Specific Professions/Statuses: Samples included military volunteers, medical students, and university students.
- Gender Imbalance: Some studies were restricted to one gender, such as only women or only men.

#### 3.40.4. The “Withdrawal Reversal” Confounder

The most prominent risk of bias across these studies is the potential for caffeine withdrawal reversal to be mistaken for a net enhancement of performance.

- Abstinence Requirements: Most protocols required habitual caffeine users to abstain for 10 to 24 h before testing.
- Impact of Withdrawal: Caffeine withdrawal is a documented syndrome characterized by headache, lethargy, and reduced alertness. Consequently, the improvements observed after caffeine administration may reflect the restoration of baseline functioning by alleviating withdrawal symptoms, rather than a true performance gain.
- Mitigation Efforts: Some researchers attempted to control for this by including non-habitual or low consumers or by comparing habitual users with non-users to see if effects persisted.

Across the 63 studies, only 6 studies directly examined the withdrawal reversal issue: two explicitly assessed caffeine withdrawal or abstinence effects [58,61], and two addressed the issue by comparing habitual and non-/low-caffeine users or testing whether effects differed by habitual use [59,64]. A larger subset controlled recent caffeine exposure through short pre-session abstinence periods, but these designs do not fully rule out withdrawal reversal, particularly in habitual users. Several studies instead minimized the confound by recruiting low-, non-, or infrequent caffeine consumers. Study [78] examined habitual daily caffeine intake as a continuous predictor of behavioral and ERP Stroop effects. This is directly relevant because the study explicitly framed itself as avoiding the usual acute-administration/withdrawal confound. Study [78] examined recent caffeine intake and deviation from usual caffeine intake in the prior 24 h as predictors of baseline ERP measures in athletes.

#### 3.40.5. Issues with Blinding and Study Design

While many studies employed a rigorous double-blind, randomized crossover design, others had weaker controls:

- Single-Blind or Open Designs: At least one study was single-blind, and another used an open, within-subject design where the participants and researchers were aware of the conditions. These designs are highly susceptible to participant expectancy and researcher bias.
- Expectancy and Placebo Effects: Research indicates that the ritual of drinking coffee (sensory cues and expectations) can elicit physiological and behavioral changes independent of caffeine. One study specifically found that caffeine produced larger effects than expectations, but that expectations alone did not significantly influence ERP components.
- Washout Periods: While most used a one-week washout, some had very short periods, such as four hours, which may be insufficient to clear previous caffeine from the system fully.

#### 3.40.6. Inter-Individual Variability

Several studies reported substantial inter-individual variability in participants’ responses to caffeine. This variability can obscure mean differences, suggesting that group-level conclusions may not apply to all individuals.

Additionally, some studies reported modest or nonsignificant behavioral results despite finding changes in neural markers. This discrepancy suggests a potential “file drawer” risk, in which subtle neural changes are highlighted while a lack of real-world behavioral impact is downplayed. Conversely, in some cases, behavioral improvements were noted without corresponding changes in neural markers.

#### 3.40.7. Implications of Risk of Bias for Interpretation of Key ERP Findings

The risk of bias assessment materially affects the confidence that can be placed in the main ERP conclusions. The most consistent finding of the review—shortening or preservation of P300/P3b latency—is supported by several randomized, double-blind, placebo-controlled crossover studies and is therefore less vulnerable to isolated study-level bias than amplitude-based findings. However, this conclusion should still be interpreted with caution because many studies used small samples, heterogeneous paradigms, and abstinence periods that may have induced partial caffeine withdrawal in habitual users. Consequently, some apparent caffeine-related improvements in P300 latency or behavioral speed may reflect reversal of withdrawal-related slowing rather than enhancement above an individual’s normal caffeinated baseline.

Risk of bias is especially important for interpreting P300/P3b amplitude. Amplitude effects were more heterogeneous than latency effects and were reported in several studies with small samples, specific populations, or weaker control conditions. Small samples increase imprecision and make amplitude estimates vulnerable to outliers. At the same time, inadequate blinding or sensory differences between caffeine and placebo conditions may amplify expectancy-related changes in arousal and task engagement. Therefore, caffeine-related P300 amplitude increases should be interpreted as suggestive and context-dependent, particularly credible when observed in fatigue, sleep deprivation, or high-demand paradigms with adequate placebo control, but not as definitive evidence of a general amplitude-enhancing effect.

The ROBINS-I evaluations further indicate that nonrandomized, open-label, pre–post, or cross-sectional studies should not be weighted equally with randomized controlled trials when drawing mechanistic conclusions. Such studies are useful for hypothesis generation, especially regarding habitual use, withdrawal, and real-world caffeine exposure. Still, they are more vulnerable to confounding by baseline arousal, caffeine tolerance, expectancy, sleep status, and participant selection. Accordingly, the synthesis gives greater interpretive weight to randomized, blinded, placebo-controlled crossover studies, particularly those that verify biological abstinence or caffeine exposure, and uses weaker designs primarily as supportive or contextual evidence.

### 3.41. Effects of Acute Versus Chronic Exposure on Caffeine in ERPs

The effects of acute versus chronic exposure in ERPs are presented in Table 5.

### 3.42. Effects of Caffeine on ERPs in Habitual and Non-Habitual Users

The effects of caffeine on ERPs in habitual and non-habitual users are presented in Table 6.

### 3.43. Acute Caffeine in Low or Low–Moderate Habitual Consumers: Larger and Cleaner ERP Effects

The strongest and most interpretable acute effects tend to appear when participants are low-habitual users or explicitly selected to minimize tolerance.

Study [21] is a good example. Low-habitual caffeine-consuming taekwondo athletes received a single 200 mg dose. After mental and physical fatigue, caffeine increased P300 amplitude at Cz and Pz, while latency was unchanged. This pattern suggests enhanced resource allocation, not faster evaluation. Because the sample was low in habitual intake, the ERP effect is less likely to reflect reversal of dependence and more likely to reflect a genuine acute pharmacological response. Study [43] also deliberately recruited low consumers (<150 mg/day) to avoid tolerance and withdrawal confounds.

Here, 200 mg caffeine increased the visual N1 amplitude after ingestion but did not selectively alter cue-related attentional subcomponents. Again, the effect was present but relatively specific: caffeine amplified early sensory–attentional processing without globally reshaping the task’s attentional architecture. Study [38] went further by recruiting non- or infrequent consumers. After 250 mg caffeine, P300 latency shortened markedly in the auditory oddball task, while amplitude did not change. This is important for the reviewer’s concern: in low consumers, caffeine can produce a relatively “pure” acute speeding of evaluation, with less ambiguity about whether the effect reflects tolerance reversal. Together, these studies show that low baseline intake tends to reveal clearer acute ERP effects. However, the form of the effect still depends on task demands: sometimes amplitude increases ([21]), sometimes latency shortens ([38]), and sometimes earlier sensory–attentional components are affected ([43]).

### 3.44. Acute Caffeine in Moderate or Habitual Users: Smaller, More Variable, and Often Topographically Restricted Effects

In moderate or habitual users, acute caffeine still affects ERPs, but the pattern becomes more heterogeneous, which is exactly what one would expect if tolerance and conditioned responses are operating. Studies [22,30] are highly similar: moderate users received 400 mg caffeine, and both found a significant shortening of frontal P300 latency at Fz, with weaker or nonsignificant effects at Cz/Pz. Amplitude did not change reliably. This suggests that, in habitual users, caffeine may still speed processing, but the effect is attenuated and regionally restricted, especially to frontal attentional control systems. Study [26] also used regular moderate coffee drinkers. Here, caffeine shortened P300 latency but reduced P300 amplitude across blocks. That is the opposite of [21], where the amplitude increased. This difference is likely meaningful: in habitual users, acute caffeine may shift processing toward greater efficiency/less neural recruitment in some contexts, rather than larger positivity. Studies [50,51] included habitual users and found caffeine-related increases in frontal P2 and, in [50], later P3 amplitude, despite limited or absent behavioral effects. These findings fit a pattern in which habitual users still show acute ERP modulation, but the effects are often subtle, component-specific, and not necessarily mirrored by behavior.

Study [41] is especially important for the reviewer’s point. In habitual coffee drinkers, both caffeinated and decaffeinated coffee produced ingestion-related changes, and the omnibus ERP analysis showed no clear caffeine vs. placebo group difference. Only post hoc analyses indicated a modest caffeine-specific increase in Cz. This implies that, in habitual consumers, coffee-related sensory cues, expectancy, and learned rituals can mimic or mask acute caffeine–ERP effects. So, compared with low users, habitual users show weaker, noisier, and more confounded acute ERP responses. This is exactly why pooling them with low users risks overgeneralization.

### 3.45. Withdrawal State Can Produce ERP Changes by Itself, and Acute Caffeine May Partly Reflect Withdrawal Reversal

Study [57] directly examined caffeine withdrawal in habitual users consuming at least 300 mg/day. During abstinence, auditory P300 amplitude declined, while visual P300 latency shortened. This is crucial: ERP changes occurred without any acute caffeine administration, showing that baseline abstinence status can alter ERPs in its own right. Study [60] found that withdrawal produced strong subjective sedation but only subtle ERP changes overall; however, early auditory ERP amplitude changes correlated with habitual intake during caffeine resumption. This suggests that the electrophysiological impact of abstinence/resumption is not uniform, and its magnitude depends partly on prior consumption level. Studies [24,26,29,33,34,37,49,50,51] all required caffeine abstinence before testing, typically 12–24 h. In moderate or heavier regular users, that abstinence window may have placed at least some participants into early withdrawal, meaning that part of the apparent “acute caffeine effect” could reflect restoration to baseline rather than enhancement above baseline. That possibility is supported indirectly by the study [27]. In the oddball task, placebo led to a drop in P300 amplitude over repeated recordings, whereas caffeine prevented that decline. The authors interpreted this as counteracting habituation or reduced alertness. But in habitual users after extended abstinence, this “protection” may partly reflect reversal of emerging withdrawal-related underarousal. Similarly, study [41] suggests that habitual coffee drinkers experience electrophysiological changes when consuming coffee, likely because learned cues and expectancy are intertwined with prior dependence history.

### 3.46. Tolerance and Baseline Caffeine Intake Alter Not Only the Effect Size, but Also the Direction of ERP Effects

The studies do not simply show that habitual intake weakens effects. They show that baseline intake can change which ERP parameter moves. In lower-use samples, caffeine often produced either:

- Larger amplitudes ([21,37]);
- Or clearer latency shortening ([38,43] for N1 amplitude increase rather than latency).

In habitual users, the effects were more mixed:

- Latency shortening without amplitude change ([22,30,38] if treated as low/infrequent users but still useful mechanistically);
- Amplitude reduction with latency shortening ([26]);
- Modest frontal positivity changes ([50,51]);
- Or minimal/no ERP effect despite behavioral change ([24,46]).

This pattern is compatible with tolerance. If the same dose is given to both low and habitual users, the habitual group may show:

- Smaller net pharmacological effects;
- More dependence on task context;
- More contamination by abstinence reversal or expectancy.

Study [63] adds another angle. Baseline habitual intake affected sensory gating physiology: high users had lower baseline S2 amplitudes than low users, even though acute caffeine impaired gating similarly across groups at higher doses. So habitual intake can alter the starting electrophysiological state, even when the acute challenge effect looks similar at the group level.

### 3.47. Fatigue and Sleep Loss Substantially Change Caffeine’s ERP Profile

A second major stratification missing from a pooled discussion is baseline arousal state. The caffeine–ERP effect in rested participants differs from that under fatigue or sleep deprivation.

In fatigued or sleep-deprived states, caffeine often acts most clearly on earlier or arousal-linked components, and less on late executive components.

Study [25] after 36 h of sleep deprivation is the clearest example. Caffeine enhanced P2 amplitude and shortened P2 latency, with some modest effects on No-Go-N2, but it did not restore P3 amplitude or latency. So under severe sleep loss, caffeine mainly preserved early perceptual–attentional responsiveness, not higher-order executive control. Study [35] similarly found that caffeine increased N1 negativity and P3 amplitude, especially in fatigued participants, while P3 latency remained unchanged. This indicates that fatigue increases the room for caffeine to enhance energetic/resource allocation aspects of processing, even when central evaluation speed is not accelerated. Study [42] reported that fatigue reduced N2 and P3 amplitudes, and that caffeine increased N2 and P3 amplitudes and shortened P3 latency, with effects strongest in fatigued subjects. Here, caffeine appears to normalize higher-order selective attention when arousal is compromised partially. Study [56] is also informative: a low-dose caffeinated energy supplement attenuated the fatigue-related rise in visual P3 amplitude across a long session and transiently shortened P3 latency at 30 min. The interpretation is different from [21] or [37]: a larger P3 is not always “better.” In prolonged tasks, rising P3 amplitude may reflect compensatory effort, and caffeine may reduce that compensatory demand.

Across fatigue studies, caffeine does not have a single ERP signature. Depending on the task and baseline state, it may:

- Raise amplitude by restoring diminished resources ([21,35,42]);
- Shorten latency by preserving speed ([42,56,58]);
- Or reduce a fatigue-related compensatory increase ([56]).

This is exactly why acute caffeine effects should be separated by state of arousal.

### 3.48. Some “Acute Caffeine” Studies Are Actually Mixture or Expectancy Studies and Should Not Be Used Interchangeably with Pure Caffeine Challenges

A further reason not to overgeneralize is that several studies are not pure acute caffeine tests.

Studies [44,45,56,58] used caffeine combined with glucose, citicoline, taurine, glucuronolactone, guarana, or vitamins. Their ERP changes cannot be attributed confidently to caffeine alone. For example, ref. [44] reported broad early- and late-ERP enhancements, but the drink contained both caffeine and glucose. Study [62] isolated caffeine from expectation and found that caffeine increased Attention-P3 and No-Go-P3, whereas expectation alone did not. This helps validate some acute effects, but it also shows why expectancy must be considered, especially in habitual coffee drinkers. Study [41] showed that the coffee ritual itself altered ERPs, with caffeine adding only a modest increment. This is particularly relevant for chronic users, where conditioned effects may be substantial. These studies are useful, but they should be discussed separately from pure acute caffeine administration in abstinent participants.

## 4. Discussion

The studies reviewed show that caffeine-related ERP effects are heterogeneous across components, paradigms, doses, and participant states. This variability likely reflects differences in baseline arousal, fatigue, task demands, habitual caffeine use, and the specific functional meaning of each ERP measure. Accordingly, the findings are best interpreted within a state-dependent framework rather than as evidence for a uniform electrophysiological effect of caffeine. This interpretation is also constrained by study quality: the strongest support comes from randomized, blinded, placebo-controlled crossover studies, whereas findings from small, open-label, single-blind, pre–post, or nonrandomized studies were treated as lower-certainty evidence because they are more vulnerable to withdrawal reversal, expectancy effects, confounding, and imprecision.

### 4.1. Effects on P300 Amplitude

Mixed P300 amplitude findings suggest that P300 is a state- and context-sensitive index of neural resources engaged during stimulus evaluation, updating, and response control, rather than a fixed marker that should change uniformly with any stimulant. P3b amplitude scales with task-relevant resource allocation and workload, whereas P3a reflects orienting to salient or novel events shaped by attentional control and stimulus-driven capture [84,85,86,87].

At typical doses, caffeine acts primarily through adenosine receptor antagonism, especially at A1 and A2A receptors. This reduces adenosinergic inhibition and shifts the sleep–wake balance toward alertness. Higher doses may recruit additional mechanisms and side effects, which may help explain null or bidirectional ERP findings [88,89,90]. One plausible link to P300 is neuromodulation: P3 amplitude has been related to phasic activity of the locus coeruleus–norepinephrine (LC-NE) system, which is strongly influenced by baseline state and task demands [91,92]. Accordingly, the clearest P300 increases under fatigue or sleep loss may reflect the restoration of phasic gain and attentional engagement when homeostatic sleep pressure would otherwise blunt stimulus processing, consistent with evidence that sleep deprivation reduces P300 amplitude and prolongs latency [89,92,93,94]. Therefore, P300 effects should be interpreted as downstream consequences of altered neuromodulatory gain within fronto-parietal stimulus evaluation networks, not as evidence that caffeine directly stimulates a single P300 generator.

Findings in which caffeine attenuated a fatigue-related rise in P300 amplitude, or left amplitude unchanged despite better performance or shorter latency, support a complementary interpretation. In some paradigms, larger P3S may reflect compensatory effort rather than better processing, especially across repeated blocks. Because P3 amplitude depends on how limited capacity is allocated, a drug that improves efficiency may produce a smaller or unchanged P3 while behavior improves [84,85]. In LC-NE terms, caffeine may move participants from a low-arousal, low-gain state, in which larger phasic responses are needed to sustain performance, toward a more efficient operating point requiring less additional recruitment for the same or better output [91,92].

Null effects are also theoretically expected, especially in young, healthy, well-rested samples. First, P300 amplitude may show ceiling effects when vigilance is already high, and task demands are modest [84,85,88]. Second, caffeine effects may be nonlinear, with excessive arousal increasing distractibility, anxiety, and response noise [91,95,96]. Third, habitual use, tolerance, and withdrawal reversal may mask net enhancement and make effects highly dependent on abstinence status [97,98,99,100].

Task- and topography-specific differences, such as Go/No-Go versus oddball paradigms or lateralized effects, also suggest that caffeine acts upstream on attention networks and neuromodulatory tone rather than on a single “P300 generator.” Adenosine–dopamine interactions and expectancy effects may further shape executive control and effort allocation [101,102,103,104,105]. Overall, caffeine appears to restore late evaluative resource allocation when arousal is suboptimal, reduce compensatory effort when processing becomes more efficient, and produce little net amplitude change when performance is already near ceiling or when tolerance, withdrawal, expectancy, and genotype-related sensitivity (e.g., CYP1A2, ADORA2A) increase between-subject variability.

The risk of bias reduces confidence in any simple conclusion that caffeine increases P300 amplitude. Several amplitude-positive studies had small samples, specific populations, pre–post designs, or incomplete control of expectancy and withdrawal. These design features are particularly important for P300 amplitude, as it is sensitive to arousal, motivation, fatigue, task engagement, and resource allocation. Therefore, increases in amplitude should not be interpreted as a universal pharmacological amplification effect. Instead, the more defensible conclusion is that caffeine-related P300 amplitude increases are most credible when they occur in well-controlled fatigue, sleep loss, or high-demand paradigms, where caffeine plausibly restores depleted attentional resources.

### 4.2. Effects on N2 Amplitude

The N2 family comprises overlapping negativities (roughly 200–350 ms) linked to mismatch or novelty detection, selective attention, and cognitive control [106]. This functional plurality makes the heterogeneity of caffeine-related N2 findings expected, because different N2 subcomponents reflect different computations and may respond differently to changes in arousal, effort, and control strategy. This makes N2 particularly suitable for linking caffeine pharmacology to circuit-level control. If adenosine antagonism increases alertness and task engagement, N2 effects should be most evident in tasks that require medial–frontal conflict monitoring and lateral prefrontal implementation of control, rather than in paradigms with low conflict or selection demands.

In inhibition and conflict tasks such as Go/No-Go, flanker, and task-switching paradigms, the most coherent interpretation is that control is state-dependent. Conflict monitoring accounts propose that medial–frontal systems, often linked to ACC, detect conflict and signal the need for increased control [107,108]. In Go/No-Go tasks, the No-Go N2 has often been interpreted as reflecting conflict monitoring, that is, competition between a prepotent Go tendency and the task rule, rather than inhibition as a unitary mechanism [109]. If caffeine increases task engagement and alertness by antagonizing adenosine, two opposite outcomes are plausible.

If caffeine stabilizes the task set and reduces uncertainty, conflict during No-Go evaluation may decrease, and N2 may become smaller, even when performance is equal or better. This would fit the idea that caffeine improves efficiency without requiring a larger conflict signal. Such effects may arise because caffeine removes adenosinergic inhibition and facilitates catecholaminergic regulation of frontal networks, improving the maintenance of task goals and reducing lapses that would otherwise generate conflict [88,110,111].

Under fatigue or sleep pressure, however, caffeine may restore monitoring and inhibitory control, thereby producing a larger, more negative N2. In this case, the monitoring system is effectively brought back online and detects conflicts or mismatches more efficiently. This parallels the logic used for P3 protection under fatigue, but at an earlier control-related stage, and follows from adenosine’s role in sleep–wake regulation and caffeine’s ability to counteract fatigue-related reductions in arousal and cognitive efficiency [88,110,112].

This state dependence may also help explain sequential or trial history effects. By increasing tonic arousal, caffeine may sustain monitoring across trials and amplify conflict signals even when a more flexible system would otherwise downregulate monitoring after low-conflict contexts [91,92,113].

In attention-selection paradigms in which “N2” reflects N2b or selection-related negativity, caffeine-related increases in the attended–unattended separation suggest strengthened selection rather than faster timing. Caffeine reliably improves alertness and aspects of attention, particularly under low arousal, and its benefits are more consistent for detection and vigilance than for higher cognition broadly defined [88,114,115]. By increasing neural responsiveness, caffeine may enhance sensory–attentional gain, producing a stronger N2b selection contrast without changing onset latency [110,116].

Mixed results for composite peak-to-peak N2 measures are unsurprising, as these metrics combine multiple subcomponents and polarities. In one context, caffeine may reduce a P2-N2 composite by suppressing distractor processing; in another, it may increase an N2-P3 composite because the later positivity changes more strongly. Thus, apparent inconsistencies may reflect measurement rather than contradiction [84,106,110].

Null N2 effects are also plausible. Caffeine benefits are stronger for alertness and vigilance than for complex executive control, and caffeine may alter strategy, such as the speed–accuracy balance or the degree of proactive control, without consistently altering a specific N2 subcomponent across individuals [98,99,115].

### 4.3. Effects on Contingent Negative Variation and Preparatory Slow Negativity

CNV and related preparatory slow negativities are best interpreted as readouts of preparatory state-setting, that is, how strongly fronto-motor systems configure themselves in advance. CNV generation reflects coordinated activity across medial–frontal and motor regions, cingulate and frontoparietal control systems, and task-relevant sensory areas [117,118,119]. Because CNV is upstream of stimulus processing, its expression depends strongly on the extent of proactive preparation the task invites, the amount already present at baseline, and which CNV subwindow is measured [120]. This helps explain why caffeine produces less uniform CNV effects than many stimulus-locked components.

At typical doses, caffeine acts mainly through adenosine receptor antagonism, especially at A1 and A2A receptors, counteracting inhibitory, sleep pressure-related effects on neural excitability and network responsiveness. This is relevant because preparatory slow negativities are closely tied to anticipatory control, including maintenance of task goals, setting response thresholds, and preparing sensory-motor mappings. These processes are especially sensitive to arousal and catecholaminergic gain. In LC-NE terms, caffeine may shift the balance between tonic readiness and phasic responding, changing the shape and timing of CNV buildup rather than simply increasing its amplitude.

This account also explains why CNV effects are often selective rather than global. Reward cues and task-switch cues are situations in which proactive control is recruited before target onset [121,122]. Because A2A receptors interact closely with dopamine systems involved in motivation, effort allocation, and goal-directed vigor, caffeine may sharpen the separation between high- and low-value preparatory states and preferentially enhance preparation when reconfiguration is required.

Stable individual differences likely further moderate CNV effects. Baseline arousal, personality-linked arousal regulation, caffeine pharmacokinetics and pharmacodynamics, and withdrawal reversal may all shift the direction or magnitude of the response [123,124,125]. Variation in CYP1A2 metabolism and ADORA2A-related sensitivity may especially contribute to bidirectional findings in heterogeneous samples. Thus, inconsistent CNV results are expected unless studies model baseline state and caffeine use explicitly.

Time-dependent CNV patterns also fit caffeine pharmacology. Because caffeine is rapidly absorbed and has a several-hour half-life, early post-dose increases in alerting drive may enhance cue-triggered orientation or preparatory ramping. In contrast, later effects may spare task-relevant preparation while reducing early CNV as arousal stabilizes or participants strategically downregulate effort. This can produce early enhancement followed by later suppression. Within the same framework, null CNV effects can coexist with subjective or behavioral improvement if caffeine acts mainly through alertness, motivation, or reduced effort cost rather than through a consistent change in the extracted CNV measure.

### 4.4. Effects on Mismatch Negativity

MMN peak latency indexes how quickly the auditory system registers violations of short-term regularities. Because this timing is constrained by afferent delays, local recurrent dynamics, and synaptic integration, it is not surprising that caffeine did not simply accelerate MMN latency under baseline conditions [126,127,128]. At typical human doses, caffeine primarily antagonizes adenosine receptors, especially A1 and A2A, removing an inhibitory brake that accumulates with time awake and suppresses neuronal excitability and transmitter release. However, MMN latency depends less on global arousal than on when a deviance signal crosses a local detection threshold within the auditory cortex and associated frontal sources [126,127,128]. Thus, caffeine may increase gain and readiness without retiming the earliest cortical computations, which are already operating near the ceiling.

An informative exception is that caffeine shortened MMN latency when alcohol had prolonged it. Alcohol increases extracellular adenosine, largely by inhibiting the equilibrative nucleoside transporter 1 (ENT1), thereby enhancing adenosinergic suppression and promoting sedative slowing [129,130,131]. Elevated adenosine can dampen glutamate release and hyperpolarize postsynaptic targets, slowing the buildup and transmission of deviance-related prediction errors [132,133,134]. In that slowed state, caffeine may partially reverse adenosine-mediated delay, consistent with restored thalamocortical and intracortical processing and with broader evidence that caffeine counteracts alcohol-related somnolence and performance decrements through shared adenosinergic pathways [135,136,137]. Thus, caffeine may not generally speed pre-attentive deviance detection, but it may normalize it when another drug has exaggerated adenosine-related slowing.

MMN amplitude reflects the magnitude of the mismatch response and depends on the fidelity of the sensory memory trace, NMDA-dependent prediction-error signaling, and neuromodulatory precision-weighting [126,127,128,138,139,140]. Null effects of caffeine on MMN amplitude, including in older adults, are therefore plausible if adenosine antagonism increases arousal and cortical excitability without materially changing the sensory representation of the standard, the adaptation profile, or the synaptic computations that determine prediction-error magnitude. This fits the broader pharmacology: low-to-moderate caffeine mainly removes adenosinergic inhibition rather than directly driving the glutamatergic mechanisms most often invoked in MMN generation.

Aging adds an important interpretive layer. In healthy aging, MMN amplitude is often reduced, but this effect is heterogeneous and depends on factors such as the interstimulus interval and the demands placed on sensory memory maintenance [141,142,143]. If a paradigm already elicits a robust mismatch response in older adults, caffeine may have little room to further increase its amplitude. Recent animal and systems work also suggests that adenosine helps stabilize early auditory throughput rather than simply scaling novelty responses [144,145,146]. Under normal conditions, blocking that stabilizing influence may not amplify MMN. Instead, MMN amplitude may be more sensitive to manipulations that directly affect precision-weighting neuromodulators, such as cholinergic tone, or the microcircuit computations underlying prediction errors [139,147,148]. In this framework, caffeine would be expected to affect MMN most clearly when mismatch processing is already suppressed by state, such as sleep pressure or intoxication, or when task demands recruit controlled attention.

### 4.5. Effects on P2 (Amplitude and Latency)

P2 amplitude effects appear strongest and most consistently positive-going in vigilance-challenged contexts. P2 is commonly treated as a mid-latency marker of early evaluative processing and attentional or perceptual gating, intermediate between early sensory registration and later context updating processes. Reviews and empirical work link P2 to early information selection, stimulus classification, and task-relevance evaluation, often with a fronto-central distribution [149,150,151,152]. Under fatigue, this stage often shows reduced amplitude and slower processing, suggesting that early classification and resource engagement become less reliable as sleep pressure rises [153,154]. Caffeine-related restoration of P2-like positivity in those settings can therefore be interpreted as a state-normalization effect, consistent with broader evidence that caffeine improves vigilance and reaction time efficiency under sleep loss or restricted sleep [155,156,157].

Several mechanisms could support this fronto-central readiness boost without requiring a caffeine-specific effect on P2 generators. First, adenosine receptors regulate wake–sleep balance and cortical activation, and manipulating A1/A2A signaling in the prefrontal cortex affects acetylcholine release, EEG power, and behavioral arousal [158]. Second, caffeine’s arousal effects are closely tied to catecholaminergic systems, including A2A-D2 interactions in striatal circuits, and human imaging studies suggest caffeine-related changes in dopaminergic receptor availability [110,159]. Third, LC-NE models propose that increased neuromodulatory gain amplifies the processing of salient or task-relevant information. Thus, when caffeine elevates arousal, the same stimulus stream may recruit more consistent early selection or evaluation, producing a broader frontal or fronto-central P2 increase.

Mixed direction findings in Go/No-Go paradigms are also compatible with this account if P2 amplitude is treated as reflecting processing allocation and timing rather than simply “more processing.” P2 reflects multiple overlapping mid-latency operations, and its structure can vary with task demands and decomposition method [9,151,152]. If caffeine increases global arousal or gain, it may sometimes reduce the need to sustain an early evaluative stage, especially when rapid discrimination and response execution are prioritized. More efficient processing may therefore produce a smaller or briefer P2-like positivity while shifting resources to later response selection or control stages.

Latency findings provide a relatively clear marker of state restoration. Under sleep pressure, adenosine signaling increases and tends to slow or destabilize cortical processing; independently of caffeine, sleep deprivation often impairs stages ranging from early attention to later updating [153,154,160]. By blocking adenosine receptors, caffeine may counter this slowing and improve cortical excitability and synaptic responsiveness [161,162]. Accordingly, the largest P2 latency benefits would be expected under fatigue or sleep deprivation, with weaker or absent effects when baseline arousal is already adequate.

These mechanisms also help explain why P2 effects may appear relatively general rather than tightly stimulus-selective. If caffeine primarily alters global arousal and neural gain, early mid-latency components may scale without neatly mapping onto attended-versus-unattended distinctions. This fits broader evidence that caffeine’s most reliable cognitive benefits cluster around alertness, vigilance, and fatigue-related performance. At the same time, the mixed direction of P2 amplitude effects across Go/No-Go contrasts suggests that caffeine’s arousal effects interact with task structure and baseline state, producing either larger P2 as stronger early engagement or smaller, briefer P2-like positivity when processing moves more quickly into later decision or control stages.

Habitual use and tolerance may further contribute to heterogeneity, as both can alter adenosine receptor expression and shift the arousal response to an acute dose. In addition, caffeine’s state effects are often visible in broader EEG markers of cortical activation, such as reduced alpha power and reduced slow activity [163,164,165]. Overall, P2 appears to act as a mid-latency, state-sensitive gate: caffeine can increase its amplitude when the system needs stronger early engagement, shorten its latency when early evaluative speed is impaired by sleep pressure, and reduce P2-like positivity when heightened arousal allows faster progression to later response-related operations.

### 4.6. Effects on N1/N100 Family

N1 amplitude reflects early perceptual discrimination and attention-related sensory gain rather than a passive readout of stimulus energy. Directing attention reliably increases N1 family activity, and visual N1 has been linked to discrimination within the focus of attention rather than to a generic sensory transient [166,167,168,169]. This makes more negative N1 findings mechanistically interpretable: when caffeine improves readiness and attentional allocation, it may enhance early gain and discrimination, yielding larger N1 negativity and sometimes slightly earlier peaks if these operations reach criterion faster. This is especially plausible in sustained attention contexts, where caffeine most reliably improves vigilance and reaction time, particularly under sleep loss or prolonged task demands [114,115,170].

Several converging accounts explain why amplitude increases and modest latency advances can co-occur. By antagonizing adenosine receptors, caffeine disinhibits arousal pathways, including noradrenergic and dopaminergic systems, that regulate cortical signal-to-noise and responsiveness. In LC-NE terms, changing the balance between tonic arousal and phasic gain may increase the impact of relevant events while improving overall responsiveness. In this way, caffeine may make cortex more available for early discrimination and allow stable early representations to emerge more quickly, but only when the task and baseline state permit these gains to be expressed.

This same arousal-and-gain framework also accommodates null effects and reversals of direction. If auditory input is task-irrelevant, early sensory responses may be less susceptible to top-down gain modulation, so behavioral arousal may not translate into stronger N100 [167,171]. Habitual use further complicates interpretation because tolerance and withdrawal reversal can alter baseline state and, therefore, the room available for a phasic evoked response to grow.

A smaller-amplitude but earlier-latency pattern at higher-than-usual intake is also compatible with an inverted-U account. If caffeine raises tonic activation too far, cortical activity may become more desynchronized, often accompanied by reduced alpha power and increased alpha frequency, which may reduce the amplitude of time-locked components even while improving temporal responsiveness [172,173]. Thus, higher-than-usual caffeine may speed early registration while reducing phasic amplitude because tonic activation is already elevated.

### 4.7. Effects on N2b Family and Related Selective Attention Negativities

The observed N2b pattern appears process-dependent rather than tied to the component label itself. Caffeine seems to modulate the strength of selection-for-action and control signals in the N2 time range, usually more in amplitude than in latency, and the direction depends on whether the negativity reflects active selection, conflict, or inhibitory demand. This fits the broader N2 literature, which emphasizes that “N2” is not unitary and that N2b is often linked to the conscious detection or selection of task-relevant events, sometimes superimposed on other negativities when attention is engaged [106,174].

When N2b reflects enhanced selection of relevant information, such as in attended-versus-unattended contrasts or target-relevant discrimination, caffeine-related amplitude increases are naturally accounted for by a gain account. By increasing alertness and improving the efficiency of attention allocation, caffeine may increase the separation between relevant and irrelevant stimulus representations, producing a larger selection-related negativity without necessarily changing onset time. This is consistent with the broader pattern that attention manipulations commonly affect N2/N2b amplitude more reliably than latency [106,171,174].

In inhibition-heavy Go/No-Go contexts, the same arousal shift may produce either increased or decreased N2b-range negativity depending on what the negativity reflects in that task. If the N2-range effect indexes active recruitment of control, caffeine may enlarge it by strengthening proactive engagement with No-Go meaning. If, however, caffeine makes withholding less effortful by stabilizing the task set or shifting control earlier, it may reduce the conflict-related signal. Because anterior N2 amplitude depends on control demands and conflict, both effects are plausible within the N2b window [106,174].

The broader caffeine literature also predicts strong state dependence. Acute caffeine most reliably improves vigilance, alertness, and reaction time, with larger effects under fatigue or sleep loss. If N2b amplitude indexes how strongly the brain is engaging selection or control, caffeine should help prevent time-on-task declines and exaggerate attended–unattended differences when engagement would otherwise be shallow. In this sense, caffeine may reveal an existing N2b attention effect rather than create a new processing stage.

Overall, these findings can be explained by a common mechanism: caffeine antagonizes adenosine, disinhibits arousal and dopamine- or NE-linked gain systems, and shifts tonic-phasic dynamics. When this shift moves participants toward an optimal arousal range, early discrimination and mid-latency selection signals may become larger and sometimes faster. When it overshoots, or when caffeine effects are dominated by tolerance or withdrawal, the same neuromodulatory change may compress latency, attenuate phasic amplitude, or produce null effects because the relevant stream is not attended.

### 4.8. Effects on Processing Negativity (PN)

The PN pattern suggests that caffeine shifts selective processing effort in response to trial demands rather than simply amplifying the mid-latency negativity. This interpretation is broadly consistent with current models of caffeine action: by antagonizing adenosine receptors, especially A1 and A2A, caffeine removes an inhibitory constraint on neural excitability and transmitter release, increasing the likelihood that task-relevant representations will win local competition for processing resources.

Within this framework, a Go/No-Go redistribution of PN is plausible. PN is typically interpreted as an endogenous negativity reflecting selective attention to task-relevant features and sustained effort at stimulus categorization, rather than an obligatory sensory response [174,175]. If caffeine improves earlier sensory discrimination and strengthens already well-supported representations, later PN-like activity may decrease because less sustained selection is needed. Conversely, when control demands are high, especially when a response must be withheld, caffeine may be associated with greater recruitment of medial–frontal control processes, consistent with an adaptive reweighting of attentional control rather than a global gain increase [107,108,176].

Caffeine’s effects on PN may also depend on catecholaminergic systems, especially dopamine and noradrenaline, which shape signal-to-noise ratio, prioritization, and the balance between stable and flexible control. Human PET findings and A2A-D2 interactions in striatal circuits suggest one route by which caffeine could redistribute PN across Go and No-Go conditions by shifting the balance between action preparation and restraint [159].

### 4.9. Effects on Late Slow Activity After Inhibition (LP/LN/SW/NegSW)

Late slow activity likely reflects overlapping sustained control, post-decisional, and disengagement processes, all of which are sensitive to analysis choices. Its variability is therefore consistent with the idea that caffeine acts most strongly on earlier bottlenecks, such as sensory discrimination, selection-for-action, and response competition, with later activity changing only when those earlier stages alter the downstream need for sustained control.

Late slow waves after No-Go trials have often been linked to prolonged maintenance of an inhibitory set and post-inhibitory evaluation, processes that depend heavily on fronto-cingulate circuitry [108,176]. If caffeine improves control implementation earlier, late activity may either shrink because less compensatory control is needed or increase. After all, the system more strongly confirms the success of inhibition and closes out the trial. This bidirectionality is therefore not unexpected.

Mixed late effects may also reflect caffeine’s vascular actions and the susceptibility of slow-wave measures to baseline drifts, fatigue, and decomposition choices [177,178,179,180]. Thus, inconsistent findings in this component family may reflect both real heterogeneity in downstream control demands and substantial methodological sensitivity.

### 4.10. Effects on Temporal T-Complex

The Ta/Tb T-complex suggests that caffeine can alter timing in the secondary auditory cortex without necessarily changing the classic midline N1-P2 complex. This component is thought to reflect activity from lateral temporal auditory regions and can reveal asymmetries and maturational effects that fronto-central measures may miss [181,182,183]. A caffeine-related shortening of Ta latency, together with altered ipsilateral–contralateral timing, is therefore consistent with faster or more synchronous processing in secondary auditory pathways, possibly through reduced adenosine-mediated suppression of excitatory transmission and increased cortical excitability.

Because the T-complex is sensitive to developmental status and temporal-lobe functional integrity, caffeine’s effects may be most visible when temporal–cortical timing is relatively labile across individuals or under low-arousal conditions. Work on temporal auditory maturation supports the idea that these lateral components can shift even when more conventional midline responses appear unchanged [182,183,184].

### 4.11. Effects on Auditory P1

Minimal caffeine effects on auditory P1 fit well with the pharmacology. The earliest sensory components are strongly constrained by afferent drive and primary-cortex circuitry. In contrast, caffeine’s main action through adenosine receptor antagonism is more consistently expressed in sustained attention, prioritization, and control than in uniform amplification of the earliest cortical response. Although adenosine A1 receptor activation can suppress neurotransmitter release and thalamocortical excitation, these effects need not translate into large changes in a small early peak such as P1.

The absence of strong P1 effects, therefore, does not imply that caffeine does not affect the auditory cortex. Rather, it suggests that caffeine’s most behaviorally relevant effects emerge later, when the system must select, categorize, and map sensory input onto action.

### 4.12. Effects on Early Visual P1 and C1/P1-Range Activity

The early visual C1/P1 window provides an informative boundary case because it is early enough to index feedforward sensory encoding but late enough, especially for P1, to be modulated by attention. ERP studies consistently show strong P1 modulation by spatial attention, whereas C1 is less reliably affected and is more strongly influenced by stimulus geometry and configuration [185,186,187]. Against that background, weak or inconsistent caffeine effects suggest that caffeine does not reliably increase early visual sensory gain to the same extent as top-down spatial attention.

A plausible interpretation is that caffeine acts primarily on state variables such as arousal, alertness, and task engagement, as well as on downstream selection-for-action, rather than directly strengthening the earliest feedforward visual response. Under low-engagement or vigilance-challenged conditions, caffeine may stabilize early responsiveness by reducing lapses. In already engaged attention paradigms, however, there may be little room for additional enhancement.

### 4.13. Effects on Error-Related Negativity (ERN) and Error Positivity (Pe)

The action monitoring findings fit well with established functional accounts of ERN and Pe. ERN is strongly linked to the medial–frontal cortex, often the ACC. It is commonly interpreted as either a reinforcement-learning prediction-error signal conveyed via dopaminergic pathways or a conflict monitoring signal reflecting the coactivation of incompatible responses [107,188,189]. Pe is more consistently associated with conscious error awareness and post-error evaluative processing [190,191].

Caffeine’s effect on ERN and Pe is mechanistically important because these components index performance monitoring rather than stimulus evaluation alone. The ERN is usually linked to rapid medial–frontal monitoring of incorrect responses, with major contributions from the anterior cingulate cortex and adjacent medial prefrontal cortex. Reinforcement-learning accounts propose that ERN amplitude reflects a phasic error signal conveyed through dopaminergic systems to the medial–frontal cortex. In contrast, conflict monitoring accounts emphasize the detection of response conflict immediately after an erroneous action. Both interpretations are compatible with caffeine pharmacology: by antagonizing adenosine A1 and A2A receptors, caffeine can increase cortical excitability and alter catecholaminergic signaling, thereby increasing the gain of medial–frontal circuits when a mismatch between intended and executed action is detected. This provides a plausible explanation for the selective caffeine-related enlargement of the ERN reported by Tieges et al., in which 3 and 5 mg/kg caffeine increased ERN amplitude in habitual coffee drinkers during a task-switching paradigm.

The Pe provides a complementary index because it is less closely tied to immediate error detection and is more often associated with conscious error awareness, motivational salience, and later evaluative processing of the error. The same caffeine study found that Pe amplitude increased alongside ERN amplitude, suggesting that caffeine may strengthen both the early detection of an erroneous response and its later conscious or evaluative registration. This dual effect is important because it argues against a narrow interpretation in which caffeine speeds responses or increases general arousal. Instead, the ERN/Pe pattern implies enhanced action monitoring: errors may become more neurally salient, more likely to recruit evaluative processing, or more strongly tagged for subsequent behavioral adjustment. This interpretation aligns with broader evidence that performance monitoring depends on multiple neuromodulators, including dopamine, norepinephrine, serotonin, GABA, and adenosine, rather than a single transmitter system.

### 4.14. Effects on Feature-Selection Components (FSP and SN)

The resistance of FSP and SN to caffeine is informative because these components are widely taken to reflect early implementation of a feature-based selection filter: frontally expressed selection positivity and posterior selection negativity linked to prioritized processing of attended features [172,187,192]. If caffeine mainly acts by altering arousal and downstream selection-for-action, then early feature filter deployment may remain intact. At the same time, later stages, such as task-relevance evaluation, response mapping, or sustained selection under competition, may change measurably.

In this sense, caffeine may not strengthen the feature filter itself, which may already be robust under placebo, but rather reduce attentional lapses and improve the efficiency with which selected information is translated into decision and action. This interpretation is also consistent with broader ERP models in which early sensory selection remains relatively stable while later components and behavior are more state-sensitive.

### 4.15. Effects on Lateralized Readiness Potential (LRP) Onset

LRP onset marks when response preparation becomes lateralized in the motor cortex and is sensitive to bottlenecks at several stages, including stimulus evaluation, stimulus-response translation, and motor programming [193,194,195]. Earlier LRP onset after caffeine, especially under response competition, is therefore best interpreted as faster stabilization of the response code rather than as simple speeding of muscle execution.

This fits a catecholaminergic-and-control account in which caffeine increases cortical excitability and dopamine-linked response vigor, helping the task-relevant response dominate earlier when distractors or incompatible flankers would otherwise delay commitment. The absence of a consistent LRP shift across tasks is also informative: it suggests that caffeine’s benefit depends on the task’s limiting step. When the main bottleneck is evaluative rather than motoric, performance may improve without a measurable shift in LRP onset.

### 4.16. Movement-Related Cortical Potentials and Readiness Potentials

The MRCP pattern, namely reduced negativity during sustained contractions despite unchanged torque or EMG, together with lower perceived effort, fits the idea that caffeine improves motor efficiency under meaningful homeostatic load. Acute caffeine acts mainly as a nonselective antagonist at adenosine A1 and A2A receptors, thereby removing an inhibitory brake that accumulates with prolonged wakefulness and metabolic demand. In motor contexts, this may allow the same net output to be achieved with less global cortical recruitment, producing a smaller MRCP even when peripheral markers remain stable [196,197].

A complementary interpretation is that caffeine reduces the subjective cost of generating motor output by shifting central fatigue computations. Caffeine’s ergogenic effects are consistently associated with lower ratings of perceived exertion and greater tolerance of sustained work [198,199,200]. One plausible bridge is antagonism of A2A receptors in striatal circuits, which interact with dopamine signaling and may alter how effort and fatigue are weighted during action [104,201,202]. In this view, caffeine does not simply speed up movement preparation; it reduces the effort the brain must exert to maintain the same output.

Dose- and context-dependent MRCP findings are also expected. Adenosine tone is lowest when people are rested, and tasks are simple, and highest when wakefulness is prolonged, metabolic stress is elevated, or exertion is sustained. If caffeine’s benefit mainly reflects subtraction of accumulated inhibitory tone, then larger effects under heavy workload and smaller effects after everyday doses in easy tasks are entirely consistent. Thus, caffeine may leave average premovement morphology relatively intact in simple paradigms while still improving motor readiness and psychomotor reliability.

### 4.17. N2 Latency

Selective shortening of No-Go N2 latency under severe sleep deprivation is best interpreted as a state-dependent rescue of control timing rather than a stable “caffeine speeds N2” effect. Prolonged wakefulness increases sleep pressure, in part through adenosine-related mechanisms, and this is associated with slower, less reliable attention and control. By blocking adenosine receptors, caffeine can partially reverse these fatigue-related deficits and restore cortical arousal [203]. Adenosine receptor manipulations in the prefrontal cortex also affect acetylcholine release and EEG arousal, providing a plausible route by which caffeine could restore the readiness of frontal control networks involved in conflict detection and response suppression [158].

In LC-NE terms, N2 timing should be especially sensitive to position on the arousal curve. When fatigue pushes tonic arousal too low or destabilizes it, control timing degrades; stimulant-like interventions may then restore more effective phasic engagement. Under this account, caffeine-related latency benefits should be strongest when baseline state is clearly degraded, such as after total sleep deprivation, which is consistent with broader evidence that caffeine improves vigilance and sustained attention under fatigue [155,204,205].

By contrast, in rested participants performing relatively undemanding tasks, N2-related control processes may already operate near the ceiling. In such settings, caffeine may affect how strongly control networks engage more than how quickly they initiate, helping explain why latency effects are often absent outside deprivation contexts.

### 4.18. N2c

Opposing caffeine effects on Go-related N2c and No-Go-related frontal N2c-like activity in children suggest a shift in the balance between response preparation and reactive inhibitory monitoring rather than scaling of a single N2c generator. Enhanced Go-related N2c is consistent with stronger preparatory control and may reflect greater catecholaminergic support for action selection and the maintenance of task goals. More broadly, caffeine often improves psychomotor readiness and attentional control by antagonizing adenosine and modulating dopaminergic and noradrenergic signaling.

Reduced frontal No-Go N2c-like activity may have at least two interpretations. First, caffeine may permit more efficient inhibitory gating, reducing the need for later conflict-related processing after withholding. Second, caffeine may bias the system toward stronger proactive readiness, thereby reducing the need for reactive monitoring once a No-Go cue appears. Both possibilities are consistent with known adenosine–dopamine interactions in striatal circuits, including A2A-D2 mechanisms involved in action vigor and effort valuation.

Developmental context is important. Children differ in baseline arousal regulation and caffeine sensitivity, and pediatric studies report variable cognitive effects, including mixed outcomes for attention and sleep-related processes [206,207]. In hyperkinetic or related phenotypes, caffeine may improve task engagement in some individuals but increase impulsivity or disturb sleep-dependent regulation in others [208]. Thus, the observed N2c split is more consistent with state-dependent control reweighting than with a uniform enhancing effect. Caution is required in the mechanistic interpretation of this component. The brain’s bioelectrical responses measured by this component may differ between healthy individuals and adults.

### 4.19. N140-P200 Complex Amplitude

Dose-related attenuation of an early visual N140-P200 complex in hyperkinetic children shows that caffeine’s electrophysiological effects are not uniformly enhancing. Early sensory responses are highly sensitive to gain control and baseline state. Because adenosine, especially via A1 receptors, suppresses excitatory synaptic transmission, blocking it may raise ongoing excitability and alter the excitation–inhibition balance. When baseline activity rises, early evoked components may become smaller rather than larger because the system has less room to move. Thus, a reduced N140-P200 may reflect altered or normalized sensory gain rather than weaker sensory processing, especially when latency is unchanged.

A second possible contributor is caffeine’s vascular effect. Acute caffeine reliably reduces cerebral blood flow through adenosine receptor-mediated vasoconstriction [209,210,211]. Although ERPs are not hemodynamic signals, vascular changes may still alter the metabolic conditions that support sustained sensory responsiveness, potentially contributing to amplitude reductions in the occipital cortex.

This pattern may be especially evident in children with hyperkinetic profiles, as early visual processing and integration may already be atypical, making evoked responses more sensitive to caffeine-related changes in arousal-and-gain control [212]. Developmental differences in adenosine receptor maturation may also alter the extent to which caffeine perturbs sensory circuits [213]. Together, these factors make it plausible that caffeine can dampen early visual complexes in a developing and clinically atypical system without producing a consistent latency shift.

### 4.20. N170 and N250 Negative-Bias Indices in Face Processing

Null effects on N170 and N250 negative-bias indices in older adults are theoretically coherent if caffeine’s most reliable acute action is on global arousal and task engagement rather than on the specialized computations underlying face processing. N170 is widely regarded as an index of early structural encoding of faces and is relatively robust across many task manipulations [214,215,216]. Although it can show some sensitivity to emotional expression, it is not a simple marker of affective bias and is strongly shaped by perceptual and attentional constraints [217,218]. N250 is more consistently linked to familiarity and the activation of stored face representations than to immediate arousal state [219,220].

Against that background, it is not surprising that acute caffeine, despite altering subjective alertness, did not reliably change sad-versus-happy differentiation at N170 or N250. Caffeine’s effects are more often expressed as improved vigilance, faster responding, and sometimes better executive control, all of which may occur without measurable changes in early face-evoked components, especially when face processing is already highly automatized [114,219]. These null findings therefore support a useful boundary condition: caffeine may improve overall engagement with a task while leaving relatively encapsulated face-processing mechanisms largely unchanged.

### 4.21. P3a and Novelty-Related P3 Activity

The overall pattern, with P3a or novelty-P3 often unchanged and sometimes reduced, fits the idea that caffeine does not primarily strengthen bottom-up orienting but instead shifts the brain toward a more stable, goal-directed operating mode. By antagonizing adenosine A1 and A2A receptors, caffeine lifts inhibitory pressure on ascending arousal systems and striatal circuits involved in motivation and action selection. In this way, caffeine may increase alertness and improve vigilance without necessarily increasing responsiveness to irrelevant novelty.

A useful interpretation comes from LC-NE gain-control accounts. If caffeine raises baseline alertness and helps maintain task goals, orienting responses to novelty may become less pronounced because fewer attention resets are needed [220,221]. Thus, null or dampened P3a effects may reflect more efficient filtering rather than weaker stimulant action. Because P3a indexes orienting and involuntary attention shifts to unexpected events [86], caffeine-related increases in readiness can coexist with unchanged or reduced novelty responses when the system is already stabilized around task-relevant selection.

### 4.22. P450 Amplitude

An increase in P450 after a caffeine-containing multi-ingredient drink is most plausibly interpreted as greater sustained engagement or control. Caffeine reliably improves vigilance and reduces reaction time variability, effects that map naturally onto later positive activity linked to continued attention allocation and updating [156,222]. By antagonizing adenosine receptors, caffeine may facilitate arousal-promoting pathways and stabilize task set maintenance, thereby increasing late frontal or fronto-central positivity.

However, interpretation is limited because the intervention also contained citicoline. Citicoline may support membrane phospholipid metabolism and cholinergic or dopaminergic function relevant to sustained attention and working memory [156,223,224,225]. The observed P450 increase may therefore reflect synergy between caffeine-related arousal and citicoline-supported sustained control processes rather than an isolated caffeine effect.

### 4.23. Parietal NP1

The subtle, lateralized NP1 interaction fits a broader pattern in caffeine effects: early sensory responses are often relatively stable, whereas more consistent caffeine effects emerge through state regulation and later selection processes. Because caffeine’s primary action is adenosine receptor antagonism, its strongest neural influence is usually on neuromodulatory tone and network excitability rather than on uniform amplification of the earliest feedforward sensory responses. This makes a small hemisphere-dependent modulation biologically plausible while also predicting the absence of a robust global NP1 change.

Early lateralized components are also highly sensitive to task geometry and top-down biasing signals. If caffeine improves the stability of attentional control settings, its earliest measurable effects may emerge only where the task already induces strong hemispheric asymmetries. In this sense, caffeine may not broadly boost early encoding but may slightly alter how efficiently one hemisphere implements an attention bias.

### 4.24. Peak-to-Peak Visual Evoked Potential Amplitude

The finding of unchanged early VEP amplitude and latency despite marked effects of caffeine on cerebral blood flow and BOLD responses is consistent with the established neurovascular actions of caffeine. By antagonizing adenosine receptors, caffeine induces cerebral vasoconstriction and reduces baseline blood flow, a major confound for hemodynamic measures of activation [209,226,227]. Calibrated-BOLD and neurovascular-coupling studies further show that caffeine can alter the relation between blood flow and oxygenation, meaning that BOLD changes may reflect vascular state shifts as much as changes in neuronal firing [177,228,229,230].

This helps explain why an electrophysiological measure of early visual responsiveness can remain stable while hemodynamic measures change substantially. The initial cortical response to simple visual stimulation may be preserved, even as vascular tone and oxygen-delivery parameters shift. Thus, no VEP change should not be interpreted as evidence that caffeine does not affect the brain; rather, it suggests that caffeine’s most consistent neural effects may arise at later, context-dependent stages, whereas early stimulus-locked sensory responses can remain relatively robust.

### 4.25. Effects of N400

The available caffeine–ERP literature contains comparatively little direct work on language-related ERPs. Still, the N400 is important to include because it extends the review beyond attention and oddball paradigms into semantic and syntactic processing. The N400 is a centro-parietal negativity linked to meaning processing, semantic access, and the ease with which a word can be integrated into its preceding context. Larger N400 amplitudes are typically observed for semantically unexpected or incongruent words, whereas predictable or contextually supported words elicit smaller N400 responses. On this basis, caffeine would not necessarily be expected to increase N400 amplitude in a simple linear manner. If caffeine improves alertness, vigilance, and maintenance of contextual information, it could reduce semantic-processing effort and attenuate N400 amplitude for contextually constrained material. Conversely, in tasks requiring the detection of semantic anomalies, caffeine could sharpen the contrast between expected and anomalous input by improving sustained attention to sentence context.

This distinction is important because caffeine’s effects on language processing are likely to be indirect and state-dependent. Caffeine does not appear to act as a language-specific enhancer; rather, it modulates arousal, attentional control, working-memory maintenance, and executive checking, all of which can influence the neural conditions under which semantic access and integration occur. Evidence from behavioral language studies supports this interpretation. Brunyé and colleagues showed that caffeine enhanced real-world language processing during a proofreading task, with effects that depended on dose and error type [231,232]. Such findings suggest that caffeine may improve the monitoring and correction of linguistic errors, especially when the task requires sustained attention and executive control, rather than uniformly improving all aspects of lexical or semantic processing. In ERP terms, this predicts stronger caffeine effects on late monitoring or reanalysis components than on the N400 alone, particularly when language tasks require explicit error detection.

### 4.26. P600

The P600 should therefore be discussed alongside the N400. Although originally associated with syntactic violations and syntactic reanalysis, the P600 is now often interpreted more broadly as reflecting controlled integration, reanalysis, repair, or late evaluative processing in response to linguistic conflict [233,234]. Some accounts also emphasize overlap between the P600 and the P3b family, suggesting that the P600 may partly reflect domain-general context updating or task-relevant evaluation when the language system detects a conflict that requires revision. This is directly relevant to caffeine, because the most consistent ERP finding across the caffeine literature is not a uniform enhancement of early sensory components, but modulation of later attention- and evaluation-related positivities, especially P3/P300 family activity under fatigue, high workload, or task demand. Thus, if caffeine affects the P600, the most plausible mechanism is increased late-stage evaluative control, enhanced detection of linguistic conflict, or more efficient reanalysis/repair, rather than a direct effect on syntax-specific mechanisms.

### 4.27. Acute and Chronic Caffeine Exposure, Regular and Occasional Consumers

An important interpretive issue is the distinction between acute caffeine effects and effects related to habitual or chronic caffeine exposure. Most studies included in this review tested acute caffeine administration, so the conclusions mainly concern short-term modulation of ERP indices rather than long-term neuroadaptation to caffeine. However, many acute studies were conducted in regular caffeine consumers after several hours of abstinence. In these cases, observed ERP changes may reflect both acute adenosine receptor antagonism and the reversal of early withdrawal symptoms. This is especially relevant for late cognitive components such as P3/P300, CNV, and error-related activity, which are sensitive to arousal, fatigue, motivation, and withdrawal state.

The distinction between regular and occasional or low consumers is also important. Regular consumers may show tolerance to some subjective or physiological effects of caffeine, but may also show larger apparent benefits after abstinence if caffeine reverses withdrawal-related slowing, reduced alertness, or reduced attentional engagement. In contrast, occasional or low consumers may show clearer pharmacological stimulation but may also be more susceptible to overstimulation, anxiety, or sensory-gating changes at higher doses. Therefore, differences in habitual caffeine use may partly explain why some studies reported shorter P3 latency or increased P3 amplitude, whereas others found minimal ERP changes.

### 4.28. Dose, Timing of Administration, and Individual Variability as Mechanistic Moderators

Dose, timing of administration, and individual variability are central to interpreting caffeine-related EP/ERP findings. Across the included studies, caffeine was administered across a broad range of fixed and weight-adjusted doses, from low doses approximating a single cup of coffee to higher doses around 3–6 mg/kg or more. This variation is mechanistically important because caffeine effects are unlikely to be linear. Moderate doses may increase alertness, improve vigilance, and shorten P3/P300 latency, whereas higher doses may produce excessive arousal, anxiety, distractibility, or impaired sensory filtering. This may help explain why late evaluative components sometimes show faster timing without greater amplitude, and why early auditory gating measures, such as P50, may worsen at higher doses.

Timing the ERP assessment is also critical. Many studies recorded ERPs approximately 30–60 min after caffeine administration, but this does not guarantee that all participants or all ERP components were measured at their maximal caffeine-sensitive interval. Caffeine absorption and clearance vary substantially between individuals, and different neural processes may have different time courses. Early sensory responsiveness, attentional selection, late stimulus evaluation, and fatigue resistance may not peak at the same post-ingestion interval. Therefore, studies using a single post-dose measurement may underestimate or miss caffeine effects, especially if the component of interest has a delayed, transient, or state-dependent response.

Individual variability further complicates interpretation. Habitual caffeine use may produce tolerance, while acute abstinence may produce withdrawal symptoms that caffeine reverses. In such cases, improved P3 latency, increased P3 amplitude, or restored N1/P2 responsiveness may reflect the reversal of withdrawal or fatigue rather than enhancement above a true non-withdrawn baseline. Conversely, low or infrequent caffeine users may show stronger pharmacological sensitivity but also a greater risk of overstimulation. Baseline sleep status, fatigue, anxiety proneness, age, sex, body mass, oral contraceptive use, smoking status, genetic variation in caffeine metabolism, and variation in adenosine receptor sensitivity may all shift the dose–response curve.

These moderators may partly explain the apparently inconsistent component-level findings. P3/P300 latency effects may be most reliable because late stimulus evaluation is highly sensitive to arousal and vigilance. P3 amplitude may vary because caffeine can either increase resource allocation under fatigue or reduce compensatory effort when processing becomes more efficient. Early sensory components may remain unchanged in alert participants but become caffeine-sensitive during sleep loss, prolonged wakefulness, or sustained task performance. Preparatory potentials may vary depending on whether caffeine primarily changes motivational readiness, motor vigor, or perceived effort. Future studies should therefore report absolute dose, mg/kg dose, administration vehicle, abstinence duration, habitual caffeine intake, timing of ERP recording, and biological verification of caffeine exposure. They should consider repeated post-dose ERP recordings rather than a single fixed assessment point.

### 4.29. Possible Genetic, Sex-Related, and Age-Related Influences

Inter-individual biological differences may also contribute to variability in caffeine-related ERP effects. Genetic variation affecting caffeine metabolism, adenosinergic signaling, or dopaminergic function may influence both the magnitude and direction of ERP responses. Still, current evidence is insufficient to identify specific profiles that reliably moderate these effects.

Sex-related factors may also matter. Hormone status, body composition, and caffeine pharmacokinetics can all affect neural and behavioral responses to caffeine. Yet, existing ERP studies do not allow firm conclusions about systematic sex differences in caffeine-related ERP modulation.

Age is another likely source of heterogeneity because caffeine metabolism, baseline cortical excitability, attentional control, and ERP morphology all change across the lifespan. Younger and older individuals may therefore show different electrophysiological responses to caffeine, but the available evidence remains too limited and heterogeneous to define clear age-related patterns.

Future studies should examine genetic background, sex-related factors, and age more systematically as potential moderators of caffeine-related ERP effects.

### 4.30. Unified Mechanistic Framework

The findings synthesized in this review appear more consistent with a state-dependent neuromodulatory model than with a simple “caffeine increases ERP amplitude” account. At typical acute doses, caffeine primarily antagonizes adenosine A1 and A2A receptors, reducing the inhibitory effects of adenosine that accumulate with prolonged wakefulness, fatigue, and sustained task performance. This action does not appear to uniformly amplify cortical responses. Instead, it may shift the brain toward a more alert and responsive operating state, with the consequences of that shift depending on baseline state, task demands, and which stage of processing is rate-limiting in a given paradigm. It should be emphasized that the interpretation of the mechanisms of action of caffeine on EPRs is cautious due to the high heterogeneity of studies.

A receptor-informed extension of this framework helps clarify why caffeine effects may differ across ERP components. A1 antagonism is likely most relevant to early sensory processing because A1 receptors are widely distributed across cortical networks and regulate adenosine-mediated inhibition, synaptic release, and neuronal excitability. In ERP terms, this pathway may influence sensory gain, habituation, and inhibitory filtering, making it particularly relevant to P50 sensory gating, N1/N100, P2, and early AEP/VEP measures. However, the reviewed evidence suggests that these effects are not uniformly expressed. Early sensory components were often stable in rested participants, whereas N1/P2 changes or P50 gating disruption appeared more clearly under fatigue, prolonged task performance, higher-dose exposure, or other conditions in which baseline arousal or inhibitory filtering may be altered.

By contrast, A2A antagonism may be more relevant to later cognitive and control-related ERPs because A2A receptors are strongly implicated in striatal and cortico-striatal circuits and interact with dopamine D2 receptor signaling. Through this pathway, caffeine may influence effort allocation, response readiness, action selection, proactive control, and performance monitoring. This provides a plausible receptor-level explanation for why caffeine more consistently shortened or preserved P3/P300/P3b latency and modulated CNV or ERN/Pe than it uniformly amplified early sensory responses. In this interpretation, P3/P300 effects may reflect improved late-stage stimulus evaluation or context updating, CNV effects may reflect altered preparatory control, and ERN/Pe effects may reflect changes in performance monitoring or error awareness.

This receptor-component mapping should be interpreted cautiously. None of the included ERP studies directly isolated A1- versus A2A-mediated effects using receptor-selective pharmacological probes or molecular imaging. Therefore, the proposed A1–early sensory and A2A–late control distinction is best understood as a biologically plausible organizing framework rather than direct evidence that specific ERP receptor components are generated subtypes.

A useful way to organize the ERP results is along a processing hierarchy. The earliest sensory components (e.g., auditory P1, early visual P1/C1-range activity, many VEP measures) appeared comparatively resistant to caffeine, suggesting that caffeine may not reliably strengthen the earliest feedforward sensory response when baseline processing is already adequate. Mid-latency components involved in early selection and attentional gating (especially P2, N1, N2b-related activity) were more sensitive, but in a context-dependent way: caffeine could increase amplitude when additional engagement was needed, shorten latency when fatigue had slowed processing, or reduce positivity/negativity when processing became more efficient and advanced more quickly to later stages. The clearest effects appeared in late evaluative, preparatory, and control-related ERPs—including P300 latency, CNV-related preparation, action monitoring (ERN/Pe), and some motor-preparatory measures—suggesting that caffeine’s strongest electrophysiological signature may lie in changes related to improving the stability, timing, and efficiency of selective processing-for-action rather than in globally enhancing sensory encoding.

This hierarchy is also broadly compatible with neurochemical and systems-level models. Adenosine antagonism is the best-supported primary pharmacological action of caffeine, but the ERP studies reviewed here do not directly test this mechanism. In particular, adenosine–dopamine coupling, especially involving A2A-D2 receptor interactions, offers one plausible interpretive bridge from caffeine pharmacology to changes in effort allocation, response vigor, and proactive control. In parallel, models of locus coeruleus–norepinephrine (LC-NE) function may help explain why caffeine is often associated with influence components linked to selective attention, novelty evaluation, conflict monitoring, and late stimulus processing more strongly than obligatory sensory peaks. In this view, rather than acting on a single ERP generator, caffeine may influence the gain and readiness of large-scale control systems, thereby altering how strongly and efficiently different processing stages are expressed at the scalp.

A central implication of this framework is that some seemingly inconsistent findings may reflect different positions on a shared state-regulation framework, although genuine inconsistency across studies also remains possible. When baseline arousal is suboptimal—because of sleep deprivation, time-on-task, sustained effort, or other fatigue-related factors—caffeine was often associated with patterns consistent with partial restoration of diminished processing, yielding larger amplitudes, shorter latencies, or preservation of component integrity. When participants are already well-rested and performing near ceiling, the same dose may produce little change or only subtle latency shifts. In other cases, caffeine may reduce the amplitude of a component, not because processing is weakened, but possibly because less compensatory effort is required once the system is operating more efficiently. Thus, increases, decreases, and null effects may all be compatible with a state-dependent account, although they may also reflect methodological heterogeneity, task differences, or limited statistical power. However, many of the component-specific patterns summarized here are based on small studies, often with fewer than 20 participants, so apparent consistencies should be interpreted cautiously until replicated in larger samples.

**Figure 2:**
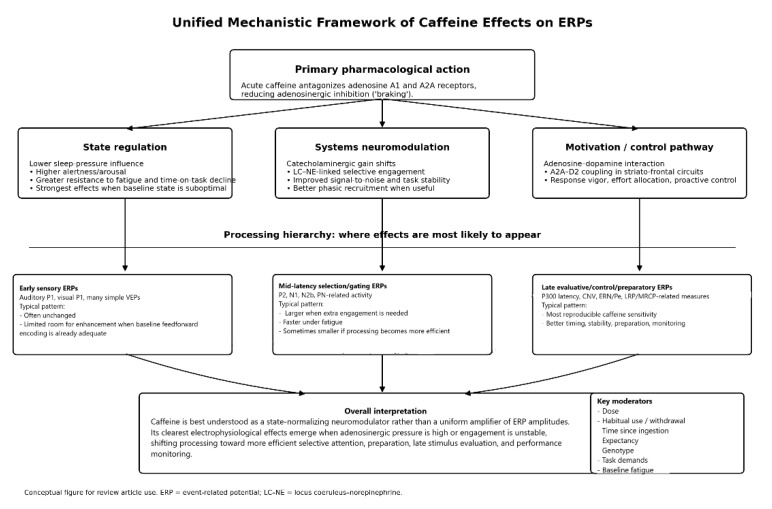
Conceptual mechanisms of action of caffeine. The framework distinguishes two partially overlapping receptor-informed pathways. The first is an A1-dominant cortical sensory/gating pathway, in which blockade of adenosine-mediated inhibition may alter cortical excitability, sensory gain, habituation, and inhibitory filtering, potentially affecting P50, N1/N100, P2, and early AEP/VEP components. The second is an A2A-dominant cortico-striatal control pathway, in which interactions with dopaminergic D2 signaling may influence effort allocation, response readiness, action selection, proactive control, and performance monitoring, with potential effects on P3/P300/P3b, CNV, ERN/Pe, and motor-preparatory potentials. Because ERP studies cannot directly assign scalp-recorded components to receptor subtypes, these pathways are intended as mechanistic hypotheses moderated by dose, timing, baseline arousal, fatigue or sleep loss, habitual use, withdrawal, task demand, and expectancy.

This framework also helps explain why heterogeneity across studies is substantial. Acute caffeine effects are shaped by dose, time since administration, habitual use, withdrawal reversal, expectancy, genetic variability, and task architecture. Components that depend strongly on strategy, effort allocation, or proactive versus reactive control are especially likely to show bidirectional effects across paradigms. Accordingly, the most parsimonious overall conclusion is not that caffeine has a single fixed ERP signature, but that it may reflect a shift in neural processing toward improved alertness, stronger selective engagement, and more efficient control under some conditions when the organism is operating below its optimal state. This unified mechanistic framework is presented in Figure 2.

Importantly, the findings were not uniform across components or paradigms. For several ERP measures, studies reported increases, decreases, or null effects, and these differences likely reflect variation in task demands, baseline arousal, dose, timing, habitual use, withdrawal control, and analytic choices. Accordingly, the mechanistic interpretation offered here should be read as a provisional organizing framework rather than as a single confirmed account.

Although caffeine’s primary pharmacological action at typical dietary doses is adenosine receptor antagonism, its ERP effects should also be interpreted in relation to downstream dopaminergic modulation. This is especially relevant for A2A receptors, which are functionally coupled to dopamine D2 receptor signaling in striatal and cortico-striatal circuits. By blocking A2A receptors, caffeine may indirectly alter dopaminergic influences on response readiness, effort allocation, action selection, reward sensitivity, and performance monitoring [136,235]. These processes are closely aligned with ERP components that were most consistently affected in the present review, including P3/P300/P3b latency, CNV or slow preparatory activity, ERN/Pe, and motor-preparatory potentials.

This dopaminergic interpretation is particularly useful for explaining why caffeine often affects late evaluative and control-related ERPs more clearly than obligatory early sensory components. P3/P300 latency shortening may reflect more efficient stimulus evaluation or faster context updating, whereas changes in CNV or slow negativity may reflect altered anticipatory control and preparation for action. Similarly, caffeine-related enhancement of ERN/Pe or reward-related slow potentials may reflect increased salience of action outcomes, improved error monitoring, or greater mobilization of control after performance feedback. These effects should not be interpreted as evidence that caffeine directly “generates” these ERP components through dopamine. Rather, they suggest that caffeine-induced adenosine blockade may shift dopaminergic control systems that influence the timing, stability, and efficiency of late-stage cognitive processing.

Caffeine-related ERP effects may also involve cholinergic mechanisms. Acetylcholine is central to cortical activation, sensory gain, attentional selection, and signal-to-noise regulation. Experimental evidence indicates that caffeine can increase acetylcholine transmission in cortical regions, including the medial prefrontal cortex, providing a plausible route through which caffeine could modulate ERP components associated with attentional gating and target detection [236]. In ERP terms, this pathway may be especially relevant to P50 sensory gating, N1/N100, P2, MMN, N2b, P3a, and P3b, all of which are sensitive to changes in vigilance, sensory filtering, novelty detection, and attentional allocation.

A cholinergic account may help explain why caffeine’s effects on early and mid-latency ERPs are variable across studies. When participants are well-rested and task demands are low, baseline sensory registration may already be sufficient, leaving little room for caffeine to amplify early components. Under fatigue, prolonged wakefulness, sustained attention demands, or higher workload, however, caffeine-related increases in cortical cholinergic tone may help preserve sensory responsiveness, sharpen attentional selection, or stabilize target processing. This could appear as enhanced or preserved N1/N100 and P2 responses, altered P50 gating, stronger N2b attentional discrimination, or increased P3a/P3b amplitude when additional attentional resources are required. In summary, multiple components are responsible for caffeine’s cholinergic modulation seen in ERPs, making it difficult to draw a single, definitive conclusion about caffeine’s cholinergic effects based on ERPs. It is important to note that studies using ERPs have varied in terms of components, paradigms, and caffeine doses. Further studies are needed to confirm caffeine-induced cholinergic modulation measured by ERPs.

### 4.31. Factors of Heterogeneity

#### 4.31.1. Individual Differences and Biological Traits

Participants’ baseline characteristics play a major role in how they respond to caffeine.

Habitual Caffeine Consumption: Habitual use can lead to tolerance or altered baseline arousal levels. Some studies focus on low-habitual consumers to avoid the confounding “withdrawal relief” effect. Others suggest that heavy users show stronger early cortical responses when caffeine is reintroduced after withdrawal.

Personality Traits: Personality significantly moderates neural responsiveness. For instance, extraverts—who are presumed to have lower baseline cortical arousal—show larger drug-related decreases in early contingent negative variation (CNV) amplitudes compared to introverts. Similarly, emotionally stable individuals may show caffeine-related ERP enhancements that are absent or reversed in emotionally labile participants.

Age: Caffeine can interact with age-related changes in “energetic” resources. In older adults, caffeine has been shown to eliminate age-related delays in P3b latency, suggesting that some cognitive slowing is due to reduced neural energy rather than structural decline. However, in children, caffeine’s effects on ERPs can be highly specific, such as amplifying active inhibitory control (N2b) while reducing post-inhibitory evaluation (fN2c).

Health and Clinical Conditions: Patients with generalized anxiety disorder (GAD) exhibit significantly greater ERP reactivity to caffeine than healthy controls, indicating heightened sensory gain. Conversely, in children with hyperkinesis, caffeine has shown a trend toward reduced ERP latency but often fails to reach statistical significance due to high inter-individual variability.

#### 4.31.2. Dosage and Pharmacokinetics

The amount of caffeine administered often dictates the type of neural response.

Dose–Response Patterns: Effects are not always linear. For example, while reaction times may improve at low-to-moderate doses (1.5 to 3.0 mg/kg), these benefits can disappear or lead to overstimulation at higher doses (6.0 mg/kg).

Threshold Effects: Some neurophysiological processes, such as P50 sensory gating, show impairment only at higher doses (200–400 mg) and are unaffected at lower doses (100 mg). In contrast, very low doses (55 mg) in energy supplements may be sufficient to reduce P3 latency and attenuate fatigue-related changes in the P2 component.

#### 4.31.3. Baseline State: Fatigue and Sleep Deprivation

Caffeine’s effects are frequently state-dependent, acting more as a compensator for impaired states than a general enhancer.

Fatigue vs. Rested States: Caffeine consistently shows more robust effects in fatigued or sleep-deprived individuals. In well-rested participants, the same dose may produce minimal or no changes in P3 latency and amplitude.

Stress: While caffeine increases physiological arousal, it may not fundamentally change how individuals respond to stress; rather, its effects are often purely additive to the stress response.

#### 4.31.4. Task Modality and Cognitive Demand

The nature of the cognitive task being performed significantly influences which neural markers are modulated.

Task Modality: Some research indicates that caffeine’s fatigue-reducing effects are more evident for visual attention than for auditory attention.

Task Complexity: Caffeine may selectively improve performance and accelerate stimulus selection (N200 latency) in choice reaction tasks but does not affect simpler reaction tasks.

Selective Attention: Caffeine often amplifies neural responses to task-relevant (target) stimuli while leaving the processing of irrelevant stimuli unchanged, suggesting it supports controlled, focused processing rather than global arousal.

Inhibitory Demand: In Go/No-Go tasks, caffeine frequently enhances components related to response execution (Go-P3b) but may have little to no effect on those related to inhibitory processing (No-Go-P3a).

#### 4.31.5. Synergistic and Antagonistic Interactions

Heterogeneity is also introduced when caffeine is consumed alongside other substances.

L-theanine: When combined with caffeine, L-theanine can produce additive improvements in selective attention and greater increases in N2–P300 amplitudes than either substance alone.

Alcohol: Caffeine can antagonize some of alcohol’s effects, such as reversing the alcohol-induced delay in MMN latency. However, it may also exacerbate impairments, such as reducing the overall magnitude of working memory engagement (N500) when co-administered with alcohol.

Taurine: Taurine has been noted to attenuate the “overshoot” of caffeine-induced acceleration in premovement cortical activity, leading to a more efficient motor preparation pattern.

Glucose: The combination of caffeine and glucose can enhance early sensory responses in the visual cortex (C1/P1) and later cognitive components (N2, P3).

#### 4.31.6. Methodological and Topographical Factors

Finally, where and how brain activity is measured contributes to differences in reports.

Site-Specific Sensitivity: Caffeine often shows more pronounced effects at frontal electrode sites (Fz) on P300 latency shortening, whereas other sites, such as Cz or Pz, may show only nonsignificant trends.

Component-Specific Effects: Caffeine may selectively modulate early perceptual processes (P2) or late cognitive evaluation (P3b), but rarely impacts the entire processing chain from sensory input to motor output.

Expectancy and Ritual: Some “caffeine effects” observed in habitual drinkers are driven by the act and ritual of drinking coffee (expectancy/conditioned responses) rather than the pharmacology of caffeine itself.

### 4.32. Generalizability Across Age, Clinical Status, and Habitual Caffeine Exposure

The generalizability of the present conclusions is limited by the demographic structure of the caffeine–ERP literature. Most included studies recruited carefully screened, healthy young adults, often university students, with restrictive eligibility criteria regarding neurological or psychiatric illness, medication use, smoking, handedness, and sensory function. This sampling strategy improves internal validity and reduces ERP variability, but it limits inference to broader populations.

Evidence from children, older adults, clinical populations, and long-term high-dose caffeine users remains comparatively sparse. Pediatric evidence is limited to a small number of studies, including typically developing children and children with hyperkinetic symptoms. Older-adult evidence is also limited, despite the likelihood that aging-related changes in baseline ERP latency, amplitude, vascular function, sleep quality, and caffeine metabolism could alter caffeine responsiveness. Clinical evidence is similarly narrow, with only limited data from groups such as anxiety-related disorders or specific neurodevelopmental/behavioral samples. Therefore, the conclusions of this review should not be generalized uncritically to children, older adults, or clinical populations.

Habitual caffeine exposure further constrains generalizability. Many studies tested low-to-moderate habitual consumers, while some recruited heavier users or imposed abstinence protocols that may have introduced withdrawal reversal. Long-term high-dose users may differ in tolerance, withdrawal susceptibility, baseline arousal, sleep disruption, and adenosinergic adaptation. As a result, acute caffeine effects observed in low- or moderate-use young adults may not directly apply to heavy daily users or individuals with long-term high-dose exposure.

Overall, the strongest conclusions of this review apply to healthy young adults under controlled laboratory conditions. Findings in children, older adults, clinical populations, and long-term high-dose users should be considered preliminary and hypothesis-generating until adequately powered, age-stratified, clinically characterized, and habitual-use-stratified studies are available.

## 5. Study Limitations and Future Directions

**Figure 3:**
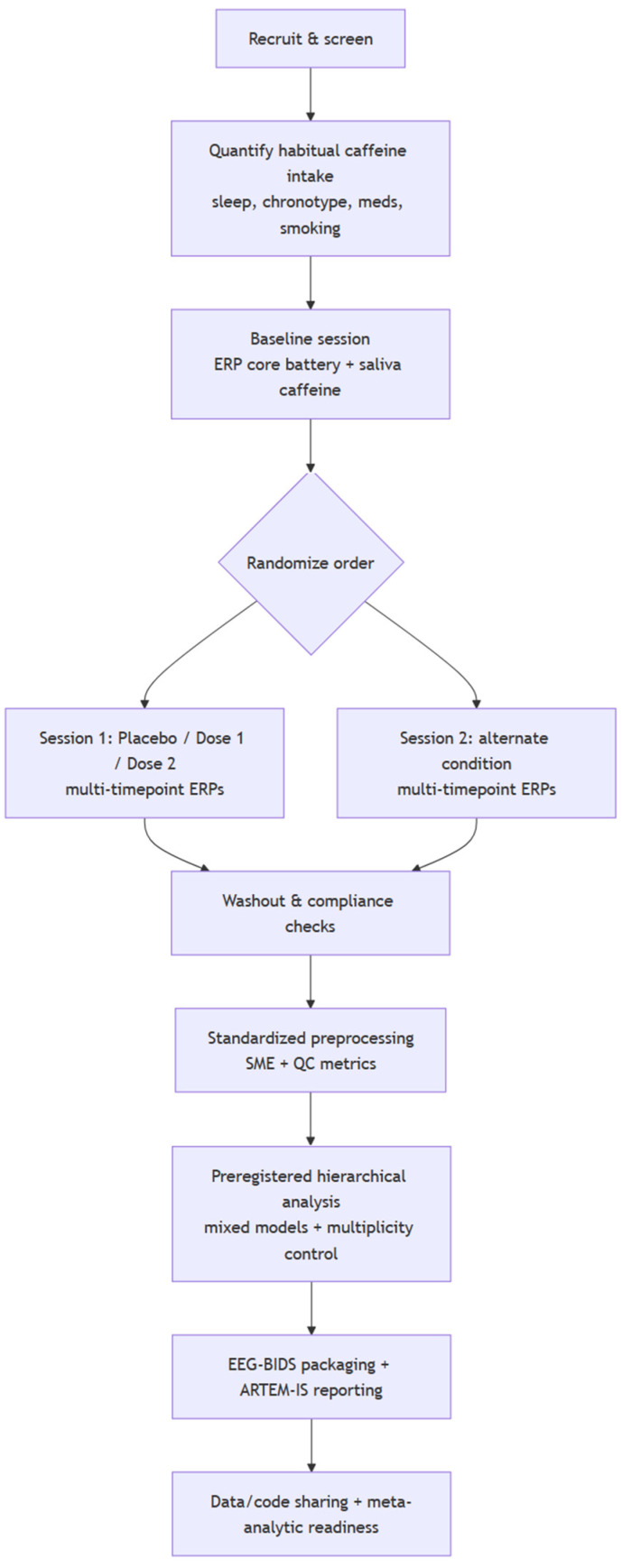
Proposed workflow for future caffeine–ERP research.

**Figure 4:**
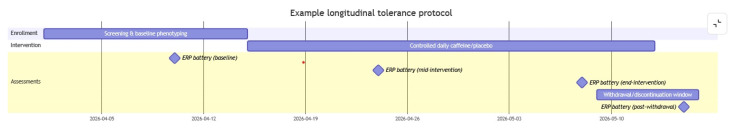
Example of protocol.

The studies included in this review have several limitations that hinder the identification of consistent patterns and reduce comparability across findings. The most important limitations, together with recommendations for addressing them in future research, are outlined below. Figure 3 presents a proposed workflow for future caffeine–ERP research, and Figure 4 illustrates an example longitudinal protocol for investigating tolerance.

### 5.1. Population Limitations and Underexamined Moderators

A major limitation of the caffeine–ERP literature is its reliance on small, often underpowered samples. Many studies included relatively few participants, which weakens statistical power, increases the risk of unstable estimates, and limits confidence in both positive and null findings. Samples have also been relatively narrow, consisting mainly of healthy young adults with restricted age ranges. In some cases, studies used single-sex samples or highly specific groups, such as athletes. As a result, the generalizability of the evidence remains limited, and it is difficult to determine whether reported ERP effects reflect broadly applicable neurocognitive mechanisms or effects specific to particular subgroups.

This limitation is especially important because caffeine’s physiological and cognitive effects are unlikely to be uniform across individuals. Age is one of the clearest potential moderators in the current evidence base. Although most studies have focused on late adolescence or early adulthood, findings from pediatric and older-adult samples suggest that caffeine’s electrophysiological effects may vary across the lifespan. In older adults, caffeine-related ERP effects may be more detectable when baseline processing is slowed, although findings remain inconsistent across paradigms and components. Pediatric findings are also mixed and may differ qualitatively from adult patterns, suggesting that developmental stage may influence both the magnitude of caffeine effects and the processing stage at which they emerge.

Sex and gender may also contribute to between-study variability, but they have rarely been examined directly. The literature includes all-male, all-female, and mixed-sex samples; however, these designs were generally used for convenience or experimental control rather than for explicit tests of sex-related differences. Consequently, the current evidence cannot determine whether males and females differ systematically in caffeine-related ERP responsiveness. Future studies should therefore include adequately powered mixed-sex samples and test sex or gender as a planned moderator rather than treating sample composition as incidental.

Genetic, metabolic, and habitual-use factors represent additional sources of heterogeneity that remain largely underexamined. None of the included ERP studies appears to have systematically tested whether variation in caffeine metabolism or adenosine signaling predicts ERP responsiveness, despite the biological plausibility of such effects. Differences in caffeine clearance or sensitivity could influence whether caffeine primarily affects early sensory, attentional, preparatory, or evaluative processes. Habitual caffeine consumption is similarly important. Regular users may differ from low-use or caffeine-naïve individuals because of tolerance, dependence, or withdrawal-related effects, yet habitual intake was often measured inconsistently and rarely treated as a primary explanatory variable. This creates a major interpretive problem: apparent enhancement after caffeine administration in abstinent regular users may partly reflect withdrawal reversal rather than improvement beyond normal baseline functioning.

Future caffeine–ERP studies should therefore recruit larger and more diverse samples and should treat individual differences as central to interpretation rather than as background characteristics. At minimum, studies should report age, sex or gender, habitual caffeine intake, abstinence duration, and withdrawal symptoms in sufficient detail to support comparison across studies. More informative designs would test these factors as planned moderators and, where feasible, incorporate metabolic or genetic measures related to caffeine clearance and adenosine sensitivity. Such work would help determine not only whether caffeine alters ERP measures on average, but also for whom these effects are most likely to occur.

### 5.2. Heterogeneity in Caffeine Administration, Timing, and Exposure Control

A second major limitation of the caffeine–ERP literature is the substantial heterogeneity in how caffeine exposure has been administered, timed, and controlled. Across studies, caffeine doses have varied widely, from relatively low amounts delivered through coffee to fixed high doses such as 400 mg, while other studies have used weight-adjusted doses, typically ranging from 1 to 6 mg/kg. This variability makes direct comparison difficult because the same nominal dose may represent a mild stimulant exposure for one participant but a much stronger pharmacological challenge for another. As a result, clear dose–response conclusions remain difficult.

The route and vehicle of administration introduce further inconsistency. Some studies used caffeine capsules, whereas others used coffee, energy drinks, or similar beverages. These vehicles are not experimentally equivalent. Capsules allow tighter control over dose and reduce sensory cues, while coffee and energy drinks introduce taste, smell, expectancy, and learned associations that may independently affect arousal and performance. In habitual consumers, the context or ritual of consuming a caffeinated beverage may itself produce conditioned responses. Consequently, observed ERP changes may reflect caffeine’s pharmacological effects, expectancy effects, withdrawal relief, beverage context, or some combination of these factors.

The timing of ERP assessment has also been inconsistent and often limited. Most studies recorded ERPs at a single post-ingestion interval, commonly 30–60 min after administration, based on the assumption that this approximates peak plasma caffeine concentration. However, caffeine absorption and clearance vary substantially across individuals, and a single recording point may miss the onset, peak, or decline of component-specific effects. It therefore remains unclear when caffeine-related ERP changes emerge, whether different components follow distinct time courses, and how long electrophysiological modulation persists.

Interpretation is further complicated by inconsistent control of habitual caffeine use, abstinence duration, and withdrawal state. Many studies required participants to abstain from caffeine before testing, but did not always standardize or thoroughly measure abstinence length, usual intake, dependence, or withdrawal symptoms. This creates an important confound. In regular users, post-dose improvements in ERP or behavioral outcomes may partly reflect reversal of withdrawal-related slowing or reduced alertness rather than enhancement above a normal, non-withdrawn baseline. Conversely, low-use or caffeine-naïve participants may show different effects because they are less tolerant and more sensitive to acute stimulation. Without careful measurement of daily intake, abstinence duration, dependence, and withdrawal symptoms, it is difficult to distinguish pharmacological enhancement from withdrawal reversal or differential sensitivity.

These issues are compounded by the fact that most studies infer caffeine exposure from administered dose rather than directly verifying internal exposure. Yet circulating caffeine levels can differ substantially between individuals who receive the same dose at the same time, due to factors such as age, smoking status, oral contraceptive use, metabolic phenotype, and other individual characteristics. Future studies would be strengthened by incorporating repeated post-dose assessments and feasible biomarkers of exposure, such as salivary caffeine. Where possible, measures such as paraxanthine-to-caffeine ratios could also help estimate metabolic differences. These approaches would allow researchers to model internal dose more accurately instead of relying only on nominal dose.

To reduce this source of heterogeneity, future work should standardize dosing, administration route, abstinence procedures, and ERP recording schedules as much as possible. At minimum, studies should report absolute and weight-adjusted caffeine dose, specify vehicle and sensory context, quantify habitual intake and withdrawal symptoms, and include multiple post-ingestion ERP recordings to capture the time course of effects. Designs that separate pharmacological effects from expectancy and withdrawal reversal, such as balanced placebo approaches or comparisons across abstinence conditions, would be especially valuable. Greater attention to exposure verification and pharmacokinetic variability would improve the interpretability, comparability, and mechanistic precision of the caffeine–ERP literature.

### 5.3. Heterogeneity in Cognitive Tasks, ERP Measures, and Analytic Procedures

Another major limitation of the caffeine–ERP literature is the substantial heterogeneity in cognitive paradigms, ERP components, and analytic procedures. Studies have used a wide range of tasks, including oddball, Go/No-Go, working-memory, vigilance, sustained attention, emotional processing, reward, and fatigue paradigms. Because these tasks engage different stages of information processing and partially distinct neural systems, caffeine is unlikely to produce a single uniform electrophysiological pattern across all contexts. This diversity makes direct comparison across studies difficult and limits the extent to which findings can be integrated into a coherent account of caffeine’s neural effects.

This variability in task design is closely tied to variability in the ERP outcomes selected for analysis. Some studies focused primarily on P300 or P3b, whereas others examined earlier sensory and attentional components such as P1, N1, and P2, or later and task-specific indices such as N2, CNV, and slow preparatory potentials. As a result, studies described broadly as examining the effects of caffeine on ERPs are often not measuring the same neurophysiological process. This helps explain why findings vary across latency effects, amplitude effects, mixed results, and null findings. Taken together, the current evidence suggests that caffeine does not have a single ERP signature, but rather produces effects that are stage-specific and dependent on task demands.

An additional source of inconsistency concerns how ERP components were defined and quantified. Studies differed in the time windows used to identify components, the scalp sites selected for analysis, the methods used to score amplitude and latency, and the broader preprocessing and data-reduction strategies applied to EEG data. Some studies averaged activity across a small number of midline electrodes, others analyzed peak values at individual sites, and others used task-specific indices that are difficult to compare across paradigms. Thus, even nominally similar components, such as P300, may not be directly comparable across studies. This problem is further exacerbated by the limited electrode coverage used in some older studies, which restricts the ability to detect spatially specific caffeine effects and reduces comparability with more recent high-density EEG work.

Reporting practices have also been inconsistent. Not all studies reported both amplitude and latency, and the rationale for selecting specific time windows, scalp sites, or scoring procedures was not always explicit. This creates concern about analytic flexibility and selective interpretation, especially because caffeine-related ERP effects are often subtle, state-dependent, and sensitive to trial loss, signal quality, and between-subject variability. Increasing sample size alone will therefore not be sufficient unless accompanied by greater measurement precision and more transparent analytic practice.

Future research should place greater emphasis on methodological harmonization. A useful step would be to adopt a shared set of well-validated paradigms covering key cognitive domains such as vigilance, response inhibition, working memory, attentional orienting, and response preparation. These paradigms should be paired with a core set of ERP components analyzed comparably across studies. Greater consistency is also needed in defining time windows, electrode selections, amplitude and latency scoring rules, preprocessing decisions, and criteria for trial inclusion or rejection. Such standardization would make it easier to determine whether inconsistent findings reflect genuine context-dependent effects of caffeine or methodological differences across studies.

At the same time, standardization should not prevent methodological development. Future studies may benefit from approaches such as time-frequency analysis, trial-level mixed modeling, or other methods capable of detecting distributed and temporally complex caffeine effects. However, these approaches should complement rather than replace a common core of interpretable ERP measures. Combining standardized paradigms with transparent and appropriately advanced analytic methods would allow the field to compare findings more directly while still capturing the complexity of caffeine’s effects on neural processing.

### 5.4. Acute Bias of the Evidence Base and the Need for Longitudinal, Dose–Response, and Repeated-Exposure Designs

A further limitation of the caffeine–ERP literature is its strong reliance on acute, short-term experimental designs. Most studies assessed electrophysiological outcomes during a single testing session and focused on the period shortly after caffeine administration, when arousal-related effects are expected to be most evident. This approach is useful for detecting immediate changes in neural processing, but it provides only a narrow view of caffeine’s effects. Acute findings alone cannot determine whether ERP changes reflect transient state effects, sustained functional enhancement, or temporary reversal of fatigue- or withdrawal-related impairment.

This limitation is important because real-world caffeine use is rarely isolated to a single exposure. Most consumers ingest caffeine repeatedly and habitually, yet the reviewed literature provides limited evidence on how repeated use influences ERP responses across days or weeks. As a result, it remains unclear whether tolerance develops, whether electrophysiological effects persist with continued intake, or whether repeated consumption changes the processing stages most affected by caffeine. Acute benefits observed in infrequent users or experimentally abstinent participants may not generalize to habitual consumers, whose responses may be shaped by tolerance, dependence, withdrawal, sleep disruption, or adaptation in arousal-regulating systems.

The lack of longitudinal and repeated-exposure designs also limits interpretation of dose effects. Although existing studies have used a broad range of caffeine doses, few have examined whether the same dose produces stable, diminishing, or changing ERP effects over time. A dose that appears beneficial during a single acute session may have weaker effects after repeated exposure, or may operate differently depending on tolerance, withdrawal state, fatigue level, or baseline arousal. Future work should therefore examine dose response not only within isolated sessions, but also across repeated-use conditions that better reflect everyday consumption.

Longitudinal studies should also include repeated post-dose ERP assessments. Measuring ERPs at multiple time points would help distinguish acute stimulation, peak effects, clearance-related changes, and withdrawal-related effects. This is especially important because different ERP components may follow different time courses. Early sensory or attentional effects may emerge rapidly, whereas later evaluative, preparatory, or fatigue-sensitive processes may show delayed or more sustained modulation. Without repeated assessments, apparent null effects may reflect measurement outside the relevant dose–time window rather than true absence of caffeine-related modulation.

Future research should therefore move beyond single-session acute paradigms toward longitudinal, repeated-measures, and explicitly dose-responsive designs. Studies that track ERP and behavioral outcomes over days or weeks of controlled caffeine intake would be especially valuable for determining whether acute effects persist, attenuate, or shift with repeated exposure. These designs should ideally include discontinuation or withdrawal phases, allowing researchers to distinguish sustained enhancement from tolerance and withdrawal reversal. By combining repeated exposure, systematic dosing, and multiple post-dose recordings, future work could provide a more realistic account of how caffeine affects neurocognitive processing over time, across doses, and under habitual-use conditions.

### 5.5. Toward a Theory-Driven and Mechanistically Informed Research Agenda

Beyond methodological improvement, future caffeine–ERP research needs a more theory-driven account of how caffeine influences neural processing. The current evidence does not support a single, uniform caffeine-related ERP signature. Instead, the mixed pattern of latency, amplitude, and null findings suggests that caffeine’s effects are conditional on the processing stage examined, the task used, and the characteristics of the participants studied. Caffeine may influence some aspects of sensory, attentional, preparatory, conflict monitoring, or evaluative processing while leaving others unchanged.

Future work should therefore move beyond broad claims that caffeine generally improves cognition and instead test specific mechanistic hypotheses. A more informative approach would ask whether caffeine primarily affects processing speed, as reflected in component latency, or neural recruitment, as reflected in amplitude. Studies should also test whether caffeine has stronger effects on early sensory–attentional selection, later evaluative processing, response preparation, or conflict monitoring. These questions are especially important under conditions in which caffeine’s effects may be more likely to emerge, such as fatigue, prolonged vigilance, novelty processing, inhibitory demand, or high cognitive load.

A theory-driven agenda should also incorporate moderators into explanatory models rather than treating them as incidental background variables. Age, sex or gender, habitual caffeine use, withdrawal state, expectancy, metabolic phenotype, and genotype are all plausible sources of variability in caffeine-related ERP responsiveness. Including these factors in preregistered hypotheses would help determine whether inconsistent findings across studies reflect methodological noise, genuine individual differences, or context-dependent neurocognitive effects.

Future studies should be designed around explicit, narrowly defined questions rather than broad exploratory comparisons across many components and tasks. Particularly valuable would be studies testing whether caffeine preferentially alters latency rather than amplitude, whether it differentially affects early sensory–attentional processes versus later evaluative or preparatory processes, and whether fatigue or task demands determine which ERP components are modulated. These questions are best addressed using harmonized paradigms that span multiple stages of processing, from early sensory–attentional components through conflict-related N2 activity to later P3-related evaluative processing. When combined with careful control of dose, timing, habitual use, abstinence, and participant characteristics, such designs can test competing accounts of caffeine as either a nonspecific arousal enhancer or a stage-dependent neuromodulator.

In this sense, the field would benefit from a small number of strategically designed benchmark studies with strong internal standardization and clearly prioritized endpoints. One priority is to establish a replicable acute effect map of caffeine using a randomized, placebo-controlled, within-subject design with a standardized ERP battery, multiple doses, and repeated post-dose assessments. A second priority is to separate pharmacological effects from expectancy and withdrawal reversal through balanced placebo designs and abstinence manipulations in both habitual and low-use participants. A third is to examine repeated caffeine use longitudinally, including discontinuation phases, to determine whether acute ERP changes persist, attenuate, or shift with continued exposure. A fourth is to combine ERP outcomes with metabolic phenotyping and, where feasible, genotype-informed analyses to test whether internal dose dynamics and biological sensitivity predict stage-specific neural responses. Together, these approaches would move the literature from cataloging inconsistent component findings toward a coherent mechanistic model of when, how, and for whom caffeine alters neurocognitive processing.

### 5.6. Translational and Applied Implications of Caffeine-Related ERP Findings

If future research produces more standardized and mechanistically interpretable findings, the caffeine–ERP literature could become more useful for applied and translational contexts. At present, the evidence does not justify simple claims that caffeine is a universal cognitive enhancer. However, several recurring patterns suggest that targeted applications may be worth investigating. Findings involving preserved or shortened P3/P3b latencies point to a possible role for caffeine in situations that require rapid stimulus evaluation under demanding conditions. Effects involving CNV, readiness-related activity, or selected N2 measures may also be relevant to sustained preparation, anticipatory control, and conflict monitoring. Translational value is therefore most likely to come from identifying which electrophysiological processes are reliably responsive to caffeine, and in which real-world contexts those processes matter.

One important area for translation is clinical research. Future studies could examine whether moderate caffeine doses improve ERP and behavioral measures in groups characterized by slowed processing, fatigue, attentional instability, or reduced alertness. In such contexts, caffeine should not be viewed as a stand-alone treatment, but it may be worth studying as a temporary countermeasure or adjunct that selectively supports information processing. ERP measures may be useful because they can detect component-specific changes even when behavioral effects are subtle or inconsistent. At the same time, any clinical application would require caution because individuals differ substantially in caffeine sensitivity, tolerance, anxiety proneness, cardiovascular risk, and sleep vulnerability. The translational value of this work will depend on determining whether predefined ERP markers can identify subgroups most likely to benefit under controlled conditions.

A second area of application concerns occupational and safety-critical settings. Caffeine is already used to sustain alertness during prolonged wakefulness, shift work, and other fatigue-sensitive conditions. ERP methods could make this research more precise by identifying which neural processes are preserved or restored by caffeine and whether those changes correspond to meaningful performance benefits. For example, reliable caffeine-related effects on P3 latency, preparatory activity, or conflict monitoring components would be especially relevant in contexts requiring prolonged vigilance, rapid signal discrimination, or sustained readiness to respond, such as transportation, military, healthcare, and monitoring environments. Future studies in these settings should move beyond general demonstrations of improved alertness and instead test intervention rules linking ERP targets to timing, dose, delivery method, and user subgroup.

Athletic and performance-oriented contexts also represent a plausible area of application. Caffeine is widely used to improve physical endurance, reaction speed, and perceived energy, and the ERP evidence suggests that some of its effects may extend to attentional engagement, response preparation, and rapid evaluation of task-relevant stimuli. These effects may be particularly relevant in sports or e-sports environments that require fast tactical decisions, sustained concentration, and efficient processing under pressure. Future work should determine whether caffeine-related changes in components such as P3 or CNV predict meaningful improvements in performance outside the laboratory.

More broadly, the long-term translational promise of this literature may lie in using ERP markers not only as mechanistic outcomes, but also as decision-support biomarkers. If component-specific effects can be replicated and linked to distinct task demands, ERPs could help guide individualized caffeine strategies by identifying which aspects of processing are vulnerable in a given context and whether caffeine improves them. However, such applications remain provisional. The current evidence base is still dominated by small laboratory studies, heterogeneous paradigms, and limited ecological validity. Future translational research should therefore focus on realistic tasks, clearly specified subgroups, and predefined ERP targets tied to actionable decisions. Only then can the caffeine–ERP literature move from general claims about possible benefits toward a practical framework for individualized and context-sensitive cognitive support.

## 6. Conclusions

This systematic and mechanistic review shows that caffeine produces conditional, component-specific EP/ERP effects rather than a uniform enhancement of cortical responses. The clearest cross-study pattern is shorter or preserved P3/P300/P3b latencies, consistent with faster or maintained late-stage stimulus evaluation and context updating. However, this latency-centered account should not be read as excluding amplitude effects: in fatigue, sleep deprivation, sustained attention, and high-demand paradigms, caffeine can also increase or restore P300/P3b amplitude, most plausibly reflecting the support of attentional resource allocation when baseline arousal or task capacity is compromised.

Amplitude-based outcomes were less consistent overall, but they were not random. P3/P300 amplitude was most likely to increase when caffeine counteracted fatigue, sleep restriction, declining vigilance, or high cognitive load. In contrast, amplitude was often unchanged in rested or lower-demand paradigms and could decrease or stabilize during prolonged/repetitive testing. Thus, caffeine does not simply amplify sensory or cognitive brain responses across all contexts. Rather, its amplitude effects appear context-dependent and may reflect either restoration of depleted resources, reduced compensatory effort, or altered efficiency depending on baseline state and task demands.

The clinical and applied relevance of these findings depends on context. Caffeine may be most useful when reduced alertness, sleep pressure, or fatigue slows stimulus evaluation and decision-related processing. However, higher doses, poor timing, withdrawal, tolerance, anxiety sensitivity, and individual pharmacokinetic differences may reduce benefit or increase variability. Therefore, caffeine-related ERP changes should not be interpreted without considering dose, post-ingestion timing, habitual use, sleep status, and baseline arousal.

For clinicians, researchers, and practitioners, the main message is that caffeine is not a general cognitive enhancer from an electrophysiological perspective. Its most reproducible effect is to preserve or accelerate late evaluative processing, particularly when performance is challenged by fatigue or sustained attention demands. Future studies should use standardized dosing, abstinence verification, repeated post-dose ERP recordings, harmonized ERP methods, standardized exercise and fatigue protocols, and multimodal neuroimaging. These approaches will clarify which individuals benefit, under which conditions, and through which neural mechanisms.

These conclusions are most applicable to healthy young adults, who constitute most of the caffeine–ERP literature. Evidence from children, older adults, clinical populations, and long-term high-dose caffeine users is limited and should be interpreted cautiously. Because age, neurodevelopmental status, clinical symptoms, habitual caffeine exposure, tolerance, withdrawal liability, sleep quality, and caffeine metabolism may all modify ERP responses, the present findings should not be assumed to generalize across the full population of caffeine consumers.

## Figures and Tables

**Table 1:** Component-level summary of consistent and inconsistent caffeine-related EP/ERP findings.

Interpretation	Consistency of Evidence	Overall Pattern Across Studies	EP/ERP Component or Component Family
Caffeine does not appear to produce a uniform amplification of early sensory encoding; effects are state- and task-dependent	Inconsistent	Often unchanged; occasional increases in N1 amplitude or shorter N1 latency, especially under fatigue or time-on-task conditions	Early sensory components: P1, N1, N100, VEP
Suggests enhanced early attentional engagement or arousal-related facilitation rather than purely sensory gain	Moderately consistent	Frequently sensitive to caffeine in attention, Go/No-Go, and sleep deprivation paradigms, often showing increased frontal positivity or shorter latency	P2
Caffeine may enhance conflict monitoring or attentional control when task demands are high	Mixed but suggestive	Increased or more negative N2/N2b reported in some attention, conflict, and inhibition tasks; null effects in others	N2/N2b/conflict-related negativity
Supports faster stimulus evaluation or context updating rather than general ERP amplification	Most consistent finding	Frequently shortened, or fatigue-related slowing is prevented, especially in oddball, attention, and sleep loss contexts	P3/P300/P3b latency
P3 amplitude is not a reliable general marker of caffeine enhancement; it likely reflects baseline state and resource demands	Inconsistent	Increased in some studies, reduced or unchanged in others; effects often depend on fatigue, task relevance, dose, and time-on-task	P3/P300/P3b amplitude
Caffeine does not consistently increase novelty orienting or inhibitory positivity	Inconsistent	Often unchanged, reduced, or selectively modulated depending on task context	P3a/novelty-P3/No-Go-P3
Caffeine may alter preparation and effort allocation, but not in a uniform direction	Inconsistent but mechanistically informative	Selective changes in anticipatory or motor-preparatory activity, but direction varies across task and personality/exertion contexts	CNV/slow preparatory activity/readiness potentials
Evidence for pre-attentive auditory deviance modulation is weak; sensory gating effects may emerge at higher doses	Limited and inconsistent	MMN usually unchanged with caffeine alone; higher caffeine doses may worsen P50 sensory gating	MMN/sensory gating/P50
Caffeine may increase performance monitoring, but the evidence base is small	Limited but suggestive	Some evidence of increased ERN and Pe amplitudes	ERN/Pe/error-related components
Current data do not support a stable conclusion regarding semantic processing	Insufficient	Sparse evidence; no clear consistent caffeine effect	N400/language-related components

**Table 2:** A summary of dose–response trends.

High Dose (5–6 mg/kg)	Moderate Dose (3–5 mg/kg)	Low Dose (1.5–3 mg/kg)	Feature
Faster, but risk of overstimulation	Consistently faster	Generally faster	Reaction Time
Can increase up to 150%	Robustly increased	Enhanced under fatigue	P300 Amplitude
Significantly shortened	Shortened under fatigue	Usually unaffected	P300 Latency
Significantly impaired (400 mg)	Impaired (200 mg)	No effect at 100 mg	Sensory Gating
Enhanced (5 mg/kg)	(Not specifically compared)	Enhanced (3 mg/kg)	Error Monitoring

**Table 3:** Risk of bias (RoB-2) assessment.

Study	Bias Arising from the Randomization Process	Bias Due to Deviations from Intended Interventions	Bias Due to Missing Outcome Data	Bias in Measurement of the Outcome	Bias in Selection of the Reported Result
[21]	Some concerns	Some concerns	Low risk	Low risk	Some concerns
[22]	Some concerns	Low risk	Low risk	Low risk	Some concerns
[24]	Some concerns	Some concerns (objective outcomes); High risk (subjective outcomes)	Low risk	Low risk for physiological/EEG; Some concerns for performance; High risk for self-reported outcomes	High risk
[25]	Some concerns	Some concerns	Low risk	Low risk	Some concerns
[26]	Some concerns	Low risk	Low risk	Low risk	Some concerns
[28]	Some concerns	Low risk	Behavioral (accuracy/RT): Low risk, EEG/N2: Some concerns	Low risk	Some concerns
[29]	Some concerns	Low risk	Some concerns	Low risk	High risk (for ERP findings); Some concerns (for RT)
[30]	Some concerns	Low risk	Some concerns	Low risk	Some concerns
[31]	High risk	Some concerns	Low risk	Low risk (QEEG/ERP); Some concerns (mood)	Some concerns
[32]	Some concerns	Some concerns	Low risk	Low risk	Some concerns
[33]	Some concerns	Low risk	Low risk	Low risk	Some concerns
[34]	Some concerns	Low risk	Some concerns	Low risk	Some concerns
[35]	Some concerns	Low risk	High risk	Low risk	Some concerns
[36]	Some concerns	Low risk	Some concerns	Low risk	Some concerns
[38]	Some concerns	Low risk	Low risk	Low risk	Some concerns
[40]	Some concerns	Low risk	Some concerns	Some concerns	Some concerns
[41]	Some concerns	Low risk	Low risk	Low risk	Some concerns
[42]	Some concerns	Low risk	Low risk	Low risk	Some concerns
[43]	Some concerns	Some concerns	Some concerns	Low risk	High risk
[44]	High risk	Some concerns	Low risk	Low risk	Some concerns
[45]	Some concerns	Low risk	Low risk	Low risk	Some concerns
[46]	Some concerns	Some concerns	Some concerns	Low risk (for objective behavioral/ERP outcomes); Some concerns (for self-report mood outcomes)	Some concerns
[47]	Some concerns	Some concerns	Some concerns	Low risk	Some concerns
[48]	Some concerns	Low risk	Some concerns	Low risk	Some concerns
[49]	Some concerns	Low risk	Some concerns	Some concerns	Some concerns
[50]	Some concerns	Low risk	Some concerns	Low risk	Some concerns
[51]	Some concerns	Low risk	Some concerns	Low risk	High risk
[52]	Some concerns	Low risk	Some concerns	Low risk	Some concerns
[53]	Some concerns	Low risk	High risk (for ERP outcomes), Some concerns (for RT)	Low risk	Some concerns
[54]	Some concerns	Low risk	Low risk	Low risk	Some concerns
[55]	Some concerns	Low risk	Low risk	Low risk	Some concerns
[56]	Some concerns	Low risk	Some concerns	Low risk	Some concerns
[58]	Some concerns	Low risk	Low risk	Low risk	High risk
[59]	Some concerns	Low risk	Low risk	Low risk	Some concerns
[60]	Some concerns	High risk	Some concerns	Some concerns (objective outcomes), High risk (subjective outcomes)	High risk
[61]	Some concerns	Low risk	Low risk	Low risk	Some concerns
[62]	Some concerns	Low risk	Some concerns	Low risk	Low risk
[63]	Some concerns	Some concerns	Low risk	Some concerns	Some concerns
[64]	Some concerns	Low risk	Some concerns	Low risk	Some concerns
[66]	Some concerns	High risk	Low risk	Low risk	Some concerns
[67]	Some concerns	Low risk	Some concerns	Low risk	Some concerns
[68]	Some concerns	Some concerns	Some concerns	Low risk	Some concerns
[69]	Some concerns	Low risk	Low risk	Low risk	Some concerns
[70]	Some concerns	Low risk	Some concerns	Low risk	Some concerns
[71]	Some concerns	Low risk	Low risk	Some concerns	Some concerns
[72]	Some concerns	Some concerns	High risk	Low risk	Some concerns
[73]	Some concerns	Some concerns	Some concerns	Low risk	Some concerns
[74]	Some concerns	Low risk	High risk	Low risk	High risk
[75]	Some concerns	Some concerns	RT outcomes: Low risk, ERP outcomes: Some concerns	Low risk	Some concerns
[76]	Some concerns	Low risk	Some concerns	Low risk	Some concerns
[80]	Some concerns	Low risk	Low risk	Low risk	Some concerns
[81]	Some concerns	Low risk	Some concerns	Low risk	Some concerns
[82]	Some concerns	Low risk	Low risk	Some concerns	Some concerns
[83]	Some concerns	High risk	Some concerns	Some concerns (objective outcomes), High risk (subjective outcomes)	Some concerns

**Table 4:** Risk of bias (ROBINS-I) assessment.

Study	Bias Due to Confounding	Bias in Selection of Participants	Bias in Classification of Interventions/Exposures	Bias Due to Deviations from Intended Interventions	Bias Due to Missing Data	Bias in Measurement of Outcomes	Bias in Selection of the Reported Result
[23]	Serious	Moderate	Moderate	Low to Moderate	Moderate	Low	Moderate
[27]	Serious	Moderate	Low	Serious	Low	Moderate	Serious
[37]	Critical	Moderate	Low	Serious	Moderate	Moderate	Moderate
[39]	Serious	Low	Low	Moderate	Low	Moderate	Moderate
[57]	Critical	Serious	Low	Moderate	Moderate	Moderate	Serious
[65]	Serious	Moderate	Low	Moderate	Moderate	Low to Moderate	Moderate
[77]	Serious	Moderate	Moderate	Serious	Low to Moderate	Low	Moderate
[78]	Moderate	Moderate	Age/sex: Low, Caffeine: Serious	Low	Low	Moderate	Moderate
[79]	Serious	Moderate	Low	Serious	Moderate	Moderate	Moderate

**Table 5:** The effects of acute versus chronic exposure in ERP.

Chronic/Habitual Exposure and Withdrawal	Acute Exposure (Single Dose)	ERP Component/Process
Significant decline in auditory P300 amplitude occurs during caffeine withdrawal in habitual users	Frequently increased, particularly during states of fatigue or high task demand	P300 Amplitude
Visual P300 latency may progressively shorten during a period of complete caffeine abstinence	Often shortened, indicating faster stimulus evaluation and categorization speed	P300 Latency
Regular users show larger amplitude changes in P1-N1 and N1-P2 components when caffeine is reintroduced after withdrawal	Can enhance N1 and P2 amplitudes and shorten latencies, reflecting accelerated early perceptual encoding	Early Sensory Processing (N1, P1, P2)
Habitual daily consumption levels do not significantly predict differences in neural interference markers like the N450	Often enhances N2/N2b amplitudes, indicating increased neural contrast between relevant and irrelevant information	Conflict and Cognitive Monitoring (N2, N450)

**Table 6:** The effects of caffeine on ERPs in habitual and non-habitual users.

Effects in Non-Habitual/Low-Level Users	Effects in Habitual/Regular Users	ERP Component/Process
Low-habitual consumers show increased N1 amplitude following caffeine ingestion. In non-habitual users who are sleep-deprived, caffeine enhances P2 amplitude and shortens P2 latency, indicating accelerated early stimulus processing.	High-habitual users exhibit significantly lower baseline S2 amplitudes (stronger sensory suppression) compared to low users. Reintroducing caffeine after withdrawal in high users triggers larger amplitude changes in P1-N1 and N1-P2 components than in low users. Taking more caffeine than usual reduces N100 amplitude but speeds up its latency.	Early Sensory (N1, P1, P2)
Caffeine increases P300 amplitude in low-habitual athletes following fatigue and in sleep-deprived low users. In low-level users, the N2–P300 peak-to-peak amplitude is significantly increased by caffeine. One study found no difference between habitual and non-users in P300 response under nighttime fatigue.	Caffeine typically increases P300 amplitude and shortens latency. In habitual users, the act of drinking coffee (expectancy/ritual) may drive these changes as much as the caffeine itself. Visual P300 latency can actually shorten during complete abstinence.	Attentional Processing (P300/P3b)
In sleep-deprived non-users, caffeine shortens No-Go-N2 latencies, helping preserve neural mechanisms involved in detecting response conflict and initiating inhibitory control.	Caffeine significantly increases ERN and Pe amplitudes during error detection. However, an individual’s level of habitual daily intake does not predict the size of their neural conflict processing markers, such as the N450.	Error and Conflict Monitoring (ERN, N2, N450)
Acute caffeine ingestion impairs sensory gating similarly to the impairment seen in high-habitual users.	Acute doses (200–400 mg) impair sensory gating (increased P50 ratio).	Sensory Gating (P50)

## Data Availability

No new data were created or analyzed in this study. Data sharing does not apply to this article.

## Supplementary Materials

The following supporting information can be downloaded at: https://www.mdpi.com/article/10.3390/nu18101489/s1, Table S1. Characteristics and main findings of included caffeine–ERP studies.
