# Supplementary material for: The Caffeinated Brain Part 1: The Effects of Caffeine on Event-Related Potentials (ERPs)—A Systematic and Mechanistic Review
Source: Nutrients. 2026 May 7;18(10):1489. doi: 10.3390/nu18101489 (PMC13209804; doi:10.3390/nu18101489)
Supplement: Supplementary file 1 [file nutrients-18-01489-s001.zip › nutrients-4247416-supplementary.pdf]

Supplementary Table S1. Characteristics and main findings of included caffeine–ERP studies.

Study	Sample	Design	Caffeine dose (timing)	Fatigue / state manipulation	Task + ERP focus	Main ERP finding(s)
[21]	n=13 male taekwondo (18–25), low habitual caffeine	Double-blind crossover; 1-week washout; 3 time points	**200 mg (~3 mg/kg)**, ~1 h pre-fatigue	Mental + physical fatigue (Corsi task + taekwondo protocol)	Auditory oddball **P300** at **Fz/Cz/Pz**	After fatigue: **P300 amplitude ↑ with caffeine** vs placebo at **Cz (+27±9% vs −31±27%, p=0.028)** and **Pz (+21±9% vs +2±2%, p=0.028)**; **no latency effects**; no Fz amp effect
[22]	n=15, mean 26±5, moderate users	Randomized double-blind crossover	**400 mg** (acute)	None	Visual oddball **P300** (Fz/Cz/Pz) + Stroop + RT	**P300 latency ↓ at Fz** (313±61 ms vs 339±78 ms); Cz/Pz latency trends ↓ (ns); **amplitude ns**
[23]	n=20 (10 poor sleepers, 10 normal sleepes), mixed sex	Controlled lab; single-dose; group comparison	**2.5 mg/kg**	Sleep-quality groups (baseline difference), not deprivation	**CNV** + auditory AEP (**incl. P300**)	**CNV larger in poor sleepers** (baseline trait; P<0.001) and RT faster (P<0.01); **caffeine did not change CNV, RT, or AEP/P300**
[24]	n=20 women, habitual users	Double-blind within-subject crossover; 4 doses	**0 / 1.5 / 3 / 6 mg/kg**	Mental stress (RIP) + cold pressor	RIP-task ERP late positive (250–500 ms)	**No caffeine effect on ERP amplitude or latency at any dose**
[25]	n=16 male students, non-users	Double-blind randomized within-subject; 2 sessions	**400 mg total** (2 doses during deprivation)	**36 h total sleep deprivation**	Visual Go/No-Go ERPs (**P2/N2/P3**)	Caffeine mainly ↑ **P2 amplitude** (frontal, esp Fz) and ↓ **P2 latency** after deprivation; modest/region-specific N2 effects; **P3 (amplitude ↓, latency ↑ with deprivation) not restored by caffeine**
[26]	n=9 men, moderate users	Double-blind crossover; 2–4 wk washout	**5 mg/kg** (~275–375 mg), ERP starts ~30 min	Repetition (6 blocks)	Auditory oddball **P300** (Fz/Cz/Pz)	**P300 amplitude lower with caffeine** (main effect p<0.001) + **P300 latency shorter** (p<0.05); amplitude reduction vs placebo **grew across blocks**
[27]	n=10 (7M/3F), regular users	Single-blind crossover	**500 mg**, measured pre / +30 min / +210 min	None	Auditory **oddball vs single-tone**; P300 mainly at Pz	In **oddball**: at +30 min caffeine **P300 amplitude & area ↑ (~150% of baseline)**; **no latency change**; effect gone by 210 min. **Single-tone**: no ERP effects. Placebo showed **habituation-like P300 decrease** at 30 min in oddball
[28]	n=94 social drinkers (21–32)	Double-blind **between-subjects** (4 beverage groups)	**4 mg/kg** caffeine; alcohol to ~0.08% BrAC	Alcohol intoxication; conflict adaptation	Flanker task; **N2 (220–320 ms)** conflict monitoring	Caffeine ↑ **N2 to incompatible trials** and **disrupted normal N2 adaptation pattern** (esp after low-conflict trials); alcohol tended to blunt N2 but didn't abolish monitoring
[29]	n=11 (one exluded due to technical problems) habitual users (3–6 cups/day)	Double-blind crossover	**~250 mg**	Slow vs fast ISI task versions	Color attention task; **P2, FSP/SN, N2b, P3b**	Caffeine ↑ **early frontal P2** (general arousal); **enhanced attentional N2b effect** (greater attended–unattended contrast); **P3b for relevant targets: no change**, but **P3b ↑ for irrelevant targets**
[30]	n=15 (9F/6M), <4 cups/day	Randomized double-blind crossover; 3 sessions (1 caffeine, 2 placebo)	**400 mg**	None	Visual oddball **P300 latency** (Fz/Cz/Pz) + Stroop + RT	**P300 latency ↓ at Fz** (361±33 → 334±30 ms; p<0.05); Cz trend ↓ (ns); Pz ~no change; qualitative note: clearer frontal P300
[31]	n=10 (5M/5F), moderate users	Randomized double-blind parallel (caffeine vs placebo after deprivation)	**400 mg** after deprivation	**24 h total sleep deprivation**	Visual oddball **P300 amplitude & latency**	**No significant P300 changes** from deprivation or caffeine; no interactions
[32]	24 healthy uni students (mean age 22.9), non-smokers,	Randomized, double-blind, placebo-controlled,	**250 mg oral capsule**; task ~**30 min** post-ingestion	None (afternoon testing; 4h caffeine abstinence to	**Auditory Go/NoGo**; Go vs NoGo ERPs: P1, N1, P2, N2, P3	**Selective Go effects: ↑ Go P2** (focal midline enhancement) and robust ↑ **Go P3b amplitude** (centroparietal),

	moderate caffeine users	**within-subject crossover**; 2 sessions ~1 week apart		minimize withdrawal)	(P3b for Go; P3a for NoGo)	**no latency shifts**; **NoGo ERPs unchanged**
[33]	15 healthy undergrads (18–26), habitual coffee drinkers; non-smokers	Double-blind, placebo-controlled, **within-subject**; 3 sessions	**3 mg/kg** and **5 mg/kg** (decaf coffee); pre-session **12h abstinence**; saliva verified	None	**Task-switching** (error-prone); **response-locked ERPs**: **ERN** (0–150 ms), **Pe** (200–400 ms); plus stimulus-locked P2/N2/P3 on correct trials	**Both doses: ↑ ERN amplitude** and ↑ **Pe amplitude** vs placebo; **no low vs high dose difference**; **stimulus-locked P2/P3 unchanged** (N2 only marginal) ⇒ **error-processing selective**
[34]	16 right-handed undergrads (18–29), regular coffee drinkers	Double-blind, placebo-controlled, **crossover**; expectancy minimized (told "normal coffee" both times)	**3 mg/kg** in decaf coffee (vs lactose placebo); **12h abstinence**	None	**Visual flanker selective attention** (compatible/neutral/incompatible); ERP **P3**, **stimulus-locked LRP** (prep), **response-locked LRP**, EMG	**P3 latency:** affected by flanker difficulty, **not by caffeine**; **P3 amplitude:** no midline effect, but **right-hemisphere (C4) P3 area** ↑ with caffeine; **stimulus-locked LRP onset earlier** with caffeine (esp. flanker conditions); response-locked LRP differences disappear
[35]	30 students (18–25), regular coffee consumers; split groups	Double-blind, placebo-controlled, **crossover** within each group	**200 mg + 50 mg maintenance in decaf coffee**	**Well-rested (AM)** vs **overnight sleep deprivation** (fatigued; tested early AM)	**Choice-RT tasks targeting stages: stimulus degradation (encoding), S–R compatibility (selection), time uncertainty (motor prep); ERPs: N1, P3**	**N1: more negative + ~5–6 ms shorter latency with caffeine (occipital); fatigue lengthens N1 latency, caffeine partially normalizes. P3: amplitude ↑ (Cz/Pz), especially in fatigued; P3 latency not changed by caffeine**
[36]	18 healthy young adult women (3 excluded due to technical issues (final sample n=15; mean ~22), moderate caffeine use	Randomized, double-blind, counterbalanced **crossover**; 2 sessions 1 week apart	**6 mg/kg**	None	**3-stimulus oddball with speeded responses**; **single-trial P3b latency** (stimulus-locked vs response-locked) + mediation of RT	**Stimulus-locked P3b latency earlier** with caffeine; **response-locked P3b latency unchanged**; **P3b amplitude unchanged**; stimulus-locked latency **partially mediated (~⅓)** caffeine RT benefit
[37]	40 healthy male medical students (18–25)	**Pre–post within-subject** (each served as own control); no placebo condition described	**3 mg/kg oral**, tested **pre** and **40 min post**	None	**Auditory oddball**; ERPs at Fz/Cz/Pz: N1, P2, N2, P3; plus RT to targets	**Latency:** general decreases; **P2 latency significantly shorter** (ANOVA; site-wise post-hoc less consistent). **Amplitude: P3 amplitude ↑ robustly** at all sites (largest at Pz); earlier components amplitude ↑ but not significant
[38]	14 right-handed men (20–32), **low/infrequent caffeine** users	Double-blind, placebo-controlled, **crossover**; scanned **pre-dose** and **~30 min post-dose** with simultaneous EEG-fMRI	**250 mg oral**; post-dose ~**30 min**	None	Simultaneous **EEG + fMRI** across tasks: visual checkerboard (VEP), finger-tapping (BOLD), auditory oddball (P300)	**CBF ↓ ~19%** (vascular). Visual task: **VEP amp/lat unchanged** despite **reduced visual BOLD** ⇒ vascular effect. Oddball: **P300 latency ↓ ~80–95 ms**, **P300 amplitude unchanged**; non-target P200/novelty P300 unchanged
[39]	26 healthy adults (14F/12M), mean age 25	**Within-subject pre–post** across three sessions (2 baseline, 1 post-coffee)	**~85 mg** (1 cup coffee) during ~10-min break before session 3	None	Voluntary **ankle dorsiflexion**; **MRCP components**: readiness potential (RP), negative slope (NS), motor	**MRCP morphology:** no systematic caffeine-related amplitude change; variability mostly between baseline sessions. **Detection:** small but consistent ↑ classification accuracy (~+2% for RF/LDA) post-caffeine

					potential (MP); plus MRCP vs idle classification (BCI-style)	
[40]	15 endurance-trained men (22–30); 14 in EEG analyses, 1 excluded due to EEG artifacts	Randomized, double-blind, placebo-controlled, **crossover**; 3 drink conditions	Placebo vs **caffeine-only drink** vs **caffeine+taurine drink**	**Physical exertion manipulation**: rest → warm-up → submaximal → maximal exercise	Self-paced right-leg "kicks" during cycling stages; ERP: **readiness potential / BP** (onset, amplitude, power, topography)	Placebo: exhaustion ⇒ **BP amplitude** ↑ broadly + **frontal expansion**. **Caffeine-only: shorter BP onset** (later start), earlier/stronger central-parietal activation at lower workload; **less frontal recruitment** at max. **Caffeine+taurine:** attenuated/gradual pattern vs caffeine-only; more focused central-parietal distribution
[41]	20 habitual coffee drinkers (10F/10M), early–mid 20s	Randomized, double-blind, placebo-controlled **between-groups** (caffeine vs decaf); **pre–post** measures within participant	Decaf coffee + **6 mg/kg caffeine (≤550 mg)** vs decaf alone; all believed "regular coffee"	None (controls for expectancy/ritual)	Rest EEG + **auditory oddball** ERPs (N1/P2/N2/P3); plus mental arithmetic, cardiovascular measures	**Omnibus ERP:** significant **pre–post ingestion effect** at Cz late P3 window (≈363–386 ms) **regardless of caffeine**; no group main effect/interaction. Post-hoc: **caffeine group** showed significant ↑ amplitude (≈243–381 ms) at Cz; placebo similar direction, NS
[42]	30 young adults (18–25), moderate coffee consumers	Double-blind, placebo-controlled, **crossover**; between-subject factor for fatigue	**~200 mg**	**Well-rested daytime** vs **sleep-deprived (fatigued)**	**Visual selective search/attention**; ERPs: N1 (early), N2 (200–350 ms), P3 (300–600 ms)	**N1:** mainly relevance effects; **no robust caffeine/fatigue changes**. **N2:** fatigue ↓ amplitude; caffeine ↑ amplitude (esp. fatigued). **P3:** fatigue ↓ amplitude + ↑ latency; caffeine ↑ **P3 amplitude** (targets; strongest in fatigued) and **shortened P3 latency mainly in fatigued**; selective for relevant stimuli
[43]	n=20 healthy young adults (19–29); low habitual caffeine (<150 mg/day)	Double-blind, placebo-controlled, within-subject; 2 sessions (1–2 week washout); ANT pre + post	200 mg caffeine vs placebo; ANT again **30 min** post-ingestion	Time-on-task/fatigue indexed by placebo showing slight decline vs caffeine maintenance	Attentional Network Task; occipital **N1 (≈80–140 ms)** at **Oz** across cue types (no/central/spatial)	**Drug × Time**: post-caffeine **N1 more negative/increased amplitude** vs pre; placebo unchanged/slight reduction (fatigue-consistent). No selective change in cue-dependent N1 modulation (global enhancement across cue types)
[44]	n=40 healthy adults (18–30), right-handed	Randomized, double-blind, placebo-controlled **between-groups**	Drink: **40 mg caffeine + 60 g glucose** (330 ml); timing not precisely stated (task after consumption)	None	Rapid visual selective-attention (focus vs divide); ERPs: early **C1/P1 (≈80–100 ms)**, **N1 (≈120–180 ms)**, **N2 (≈200–320 ms)**, **P3 (≈400–500 ms)**	Early interval more positive-going (stronger contralateral effect) with drink; **N1 less negative** (likely carryover of early positivity). **N2 more negative** and **P3 larger** in drink group. No drink × attention interactions → broad processing enhancement rather than selective attention modulation
[45]	n=60 healthy adults (20–40; 27M/33F), low–moderate caffeine users	Randomized, double-blind, placebo-controlled **between-groups**	Citicoline + caffeine beverage vs matched placebo; testing **30 min** post-consumption	None	Cognitive battery (CPT, Go/No-Go, etc.); ERP focus: **P450** (working memory updating/sustained attention) across many electrodes	**Higher P450 amplitudes** for beverage group across a broad fronto-central-parietal network (strongest in frontal/prefrontal sites), interpreted as greater attentional/WM engagement during task performance
[46]	n=39 healthy older adults (53–79), light coffee consumers	Randomized, double-blind, placebo-controlled **crossover**; 4 acute treatments	"3 cups equivalent" of: caffeinated coffee; regular-CGA decaf; high-CGA decaf; placebo;	None	ERP paradigms: (1) RVIP sustained attention: **P300/P3b (280–480 ms at Pz**	Despite behavioral RVIP accuracy benefit with caffeinated coffee, **no reliable treatment effects** on RVIP P300 AUC or CNV; **no effects on MMN**; EFRT: high-CGA reduced *behavioral* negative bias, but **no**

			outcomes ~**40 min** post		**AUC) + CNV** (Fz); (2) **MMN** (110–210 ms AUC at Fz); (3) EFRT: **N170 (138–198 ms)**, **N250 (260–380 ms)** at PO7/PO8 + negative-bias indices	**significant N170/N250 bias changes**
[47]	n=12 recreationally active young women	Randomized, counterbalanced, double-blind, placebo-controlled **crossover**	**6 mg/kg** caffeine vs placebo; exercise ~**1.5 h** post-ingestion	Strong time-on-task manipulation: 100 intermittent isometric knee extensions (~15 min); perception of effort tracked	Exercise task; ERP/EEG-derived **MRCP** (motor-related cortical potential), especially at **Cz** during contraction epochs	Caffeine **attenuated MRCP amplitude** (less negative) during contraction (0–1 s and 1–2 s), not readiness/recovery → less cortical motor drive for same force. Time-on-task increased MRCP negativity over protocol (central adaptation). MRCP amplitude correlated with perceived effort
[48]	n=17 children (8–11) diagnosed "hyperkinetic" (stimulant responders)	Double-blind, placebo-controlled, within-subject **crossover**; 3 treatments	Placebo; **~3 mg/kg** (low); **~6 mg/kg** (high); testing **1 h** post	None	CPT + activity recording; visual cortical evoked responses at O1/O2, P3/P4; focus on **N140–P200** complex	**Dose-related reduction in evoked-response amplitude** (attenuation) of N140–P200, strongest occipital (right > left). Limited parietal effects (mainly right P4). Latencies largely unchanged (only weak trend)
[49]	n=16 healthy undergrads (19–29), moderate caffeine users	Double-blind, placebo-controlled, within-subject; 2 sessions (2-week gap)	**3 mg/kg** caffeine vs placebo (lactose in decaf coffee); abstain ≥12 h; timing via saliva verification (post-dose)	None	Visual selective attention (feature-based); ERPs at Fz/Cz/Pz/Oz + motor derivation C3–C4; focus on exogenous sensory effects, selection-related endogenous components, **P3**, **LRP**	No caffeine effect on early **sensory discrimination** ERPs to spatial frequency/orientation. Caffeine induced **early selection-related positivity (≈50–160 ms)** for relevant vs irrelevant stimuli (novel/anomalous). **P3 latency shortened** for targets/frequency-relevant stimuli (not others). **LRP onset unchanged** → no speed-up of response preparation
[50]	n=12 healthy young adults (20–25), habitual coffee drinkers (3–6 cups/day)	Double-blind, placebo-controlled, repeated-measures (within-subject)	**250 mg** caffeine in decaf coffee vs placebo; task ~**40 min** post	Sustained attention (~10 min) with clear time-on-task fatigue measures (but similar across conditions)	Self-paced sustained attention (Bourdon-like); ERP focus: frontal **P2 (200–240 ms at AFz/Fz/F3/F4)**; parietal **P3 (420–616 ms at Pz)**; lapse-related early negativity	Caffeine **increased frontal P2 positivity**; caffeine **increased late P3 positivity** (esp. 520–616 ms). Lapses showed a distinct early negative shift (100–152 ms) **independent of caffeine**
[51]	n=11 healthy young adults (20–25), habitual caffeine users (3–6 cups/day)	Within-subject, double-blind, placebo-controlled; 2 sessions ~1 week apart	**250 mg** caffeine vs placebo (morning); abstain ≥12 h	None	Spatial-selective attention (attend left/right; detect rare targets); ERPs: occipital **P1/N1**, later **P2/N2**, plus frontal effects	Caffeine **did not change latencies**. Main caffeine effect: **more positive frontal P2 (≈180–240 ms)** regardless of attention. Interactions: occipital **P2 attention pattern reversed** under caffeine; occipital **N2 attention effect emerged under caffeine** (stronger negativity for attended). No general amplification of early occipital P1/N1 by caffeine
[52]	n=24 typically developing children (8–12; 12F/12M), light–moderate caffeine users	Randomized, double-blind, placebo-controlled **crossover**;	**80 mg** caffeine vs placebo; timing implied pre-task	None	Equiprobable auditory go/no-go; ERP component decomposition for go and no-go (P1,	Early components mostly small/local effects; **go**: enhanced **PN** (temporal negativity), enhanced **N2c**, enhanced **P3b**. **no-go**: **N1-1 enhanced**, **N2b strongly increased** (inhibitory control), but **fN2c**

		sessions 1 week apart			N1-1, PN, N2 variants, P3 variants, slow waves)	**reduced**; **P3a reduced**; later components weak/nonsignificant
[53]	Healthy young adults (university sample); analyzed n varied ~16–23 after exclusions	Placebo-controlled, double-blind, within-subject crossover; 4 conditions (placebo/sham; caffeine; UMTS; caffeine+UMTS)	**3 mg/kg** caffeine (verified via saliva); timing across task blocks	Fatigue indexed by within-session time/segment increases in P300 timing (across all conditions)	Visual oddball target detection; ERP focus: **P300** metrics (latency, fractional area latency, AUC)	Caffeine reliably **shortened RT** and **reduced/shortened P300 timing indices** and **reduced late P300 AUC** (more efficient/less prolonged processing). UMTS exposure **no P300 effects** and **no interaction** with caffeine (combined condition ≈ caffeine alone)
[54]	24 healthy university students (13F), 17–36; non-smokers; moderate caffeine users	Randomized, double-blind, placebo-controlled, repeated-measures crossover; 2 sessions ~1 week apart	250 mg caffeine capsule vs placebo (order counterbalanced); ≥4 h caffeine abstinence pre-session	None beyond brief abstinence	Equiprobable **auditory Go/NoGo**; PCA-derived ERPs: N1, PN, P2, P3 (Go P3b / NoGo P3a), slow wave, late positivity	**Go:** faster RT + fewer omissions; **PN (Go) slightly ↑**; **Go P2 ↓**; **Go P3b ↑ robustly**. **NoGo:** P3a largely unchanged; small **NoGo slow wave ↑**; LP unaffected; N1 largely insensitive (small Go enhancement)
[55]	24 healthy young adults (mostly female), ~21; right-handed; non-smokers; moderate caffeine users	Randomized, double-blind, placebo-controlled, repeated-measures crossover; 2 sessions ~1 week apart; PCA methods	250 mg caffeine capsule vs placebo; abstained several hours pre-test	None beyond brief abstinence	Equiprobable **auditory Go/NoGo**; PCA-isolated components incl. N1-1, PN, P2/N2b (NoGo), P3b/P3a, SW1/SW2, LP	Behavioral effects minimal. **Go: N1-1 ↑**, **PN (frontal midline) ↓**, **P3b ↑**, SW2 topography shifted (more frontal negativity). **NoGo:** N1-1 ~no change; **PN ↑ (frontal)** with central redistribution; **P2/N2b ↓**; **P3a central positivity ↑ (relative redistribution)**; **SW1 ↑ positivity**, **SW2 ↑ frontal negativity + central positivity**; **LP ↑ globally**
[56]	47 recruited; 40 in final ERP (placebo vs supplement groups); young healthy adults (27W), ~26; right-handed; non-smokers; moderate caffeine users	Double-blind, placebo-controlled, **between-groups**; EEG at baseline, +30 min, +90 min	Energy supplement with **55 mg caffeine** (multi-source) vs placebo tablet; tested pre, 30 min, 90 min	**Time-on-task / accumulating fatigue** across long protocol (inferred from placebo P3 amplitude drift)	Visual oddball + auditory oddball + simple motor task; **P3 amplitude & latency**	No behavioral change. **Visual oddball:** placebo showed **P3 amplitude ↑ across sessions** (fatigue/effort); supplement **attenuated this P3 amplitude rise** (reduced fatigue-related effort). **P3 latency:** supplement **↓ at 30 min** (visual & auditory), returned to baseline by 90 min. **Motor ERPs:** no effects
[57]	13 habitual users (≥300 mg/day), 18–48 (4M/9F)	Open, within-subjects; baseline on usual intake then **4-day abstinence**; measures on withdrawal days 1, 2, 4	**Withdrawal (0 mg)** after baseline habitual intake; biochemical verification	**Caffeine withdrawal** over days	Auditory & visual oddball; **P300 amplitude/latency**	**Auditory P300:** latency unchanged; **amplitude ↓ significantly** during withdrawal. **Visual P300: latency ↓ significantly** across withdrawal days; amplitude unchanged
[58]	10 grad students (6W/4M), 20–28; mixed habitual users (5) + nonusers (5)	Double-blind, placebo-controlled, randomized crossover; 2 nighttime sessions ≥1 week apart	Capsules: **80 mg caffeine + 1 g taurine + 600 mg glucuronolactone** vs placebo; measured baseline and **~1 h post** (final testing ~midnight)	**Late-night fatigue / circadian challenge** (testing around midnight)	Auditory oddball during nighttime; **P300** + RT; plus d2 attention + mood scales	Placebo: **P300 latency** ↑ and RT slowed by midnight. Active mix: **prevented latency/RT deterioration** (latencies stayed near baseline; RT faster vs placebo at end). **P300 amplitude:** no significant change. Mood decline under placebo prevented by active mix; effects not dependent on habitual caffeine use.
[59]	30 adults: 15 young (18–23) + 15 older (60–72); non-smokers; moderate users	Double-blind, placebo-controlled; 2 sessions	**250 mg caffeine (in two doses)** in decaf coffee vs placebo; ≥12 h abstinence	**Aging** (group factor), not an induced fatigue manipulation	Visual selective search / memory search task; ERPs: **N1**, **N2b**, search-	Aging: **N1 delayed & larger**; **N2b onset delayed (~25 ms) & smaller**; P3b reduced/broader and (placebo) **latency longer**. Caffeine: **N1 amplitude** ↑ (no latency change);

					related negativity, **P3b**	**N2b amplitude** ↑ (no onset change); modest ↑ in search-related negativity later; **P3b amplitude** ↑ and **eliminated age-related P3b latency delay**
[60]	40 healthy adults (19M/21F), mean ~36; non-smokers; varying habitual intake (~360 mg/day avg)	Controlled, double-blind withdrawal/reintroduction with placebo substitution; pharmacokinetic profiling (500 mg challenge)	**Withdrawal then reintroduction** (capsule dosing during decaf beverages; exact daily dose schedule not specified in summary)	**Caffeine withdrawal syndrome** (fatigue/sedation, headache)	Battery incl. physiological + performance; **ERP measures** (early auditory components; latencies/amplitudes)	Withdrawal produced strong **subjective sedation** but **ERP latencies largely unchanged**; RT largely unchanged (minor practice). Some **early ERP amplitude changes (P1–N1, N1–P2)** correlated with habitual intake during resumption; ERPs not strongly related to metabolic rate indices
[61]	16 undergrads (8M/8F), ~21.5; right-handed; moderate users	Double-blind, placebo-controlled, within-subjects; 2 sessions 2 weeks apart	**3 mg/kg caffeine** in decaf coffee vs placebo; ≥12 h abstinence; saliva verification	Attentional demand manipulation via **focused vs divided attention** and **display load (2 vs 4 items)**	Visual search / target detection; ERPs: early P1/N1, search negativity, **P3b (latency/amplitude)**	Behavior: RT **faster** under caffeine (esp. lower load/focused), accuracy ~unchanged; load effects persist. ERPs: early sensory & search negativity **not reliably changed** by caffeine. **P3b latency shortened** (faster evaluation), strongest in easier/focused conditions; **P3b amplitude not significantly changed**. Earlier target–nontarget divergence under caffeine, but no evidence of improved suppression of irrelevant-item processing
[62]	48 healthy young adults, ~23; moderate daily users	Double-blind, within-subjects **3-session expectancy-balanced** design (coffee): DECA/told CAF; DECA/told DECA; CAF/told DECA	**Caffeinated coffee vs decaf**; sessions ≥1 week apart	**Expectation manipulation** (belief about caffeine)	RVIP sustained attention (**Attention-P3**) + Go/NoGo (**NoGo-N2, NoGo-P3**)	Behavior: no reliable caffeine effects reported. ERPs: Caffeine (CAF/told DECA) ↑ **Attention-P3 amplitude** (voltage + GFP; sources incl. TPJ/STG/precuneus); **no reliable Attention-P3 latency change**. Inhibition: **NoGo-N2 not modulated**, but **NoGo-P3 amplitude** ↑ (and GFP; sources incl. MFG/ACC). **Expectation alone** (DECA/told CAF) did **not** significantly modulate these ERPs; caffeine effects > expectancy
[63]	24 healthy young adult volunteers; split into habitual high (≥200 mg/day) vs low (<100 mg/day) users	Randomized, single-blind, placebo-controlled, within-subject; **4 sessions** ~1 week apart	Placebo, **100 mg**, **200 mg**, **400 mg** caffeine; P50 measured baseline and **15/30/60/90 min post**	None (dose-response / user-group factor)	Auditory paired-click **P50 sensory gating**; outcomes: S2/S1 ratio, S1/S2 amplitude, latency	Using summary post-dose metrics: **P50 ratio ↑ (worse gating)** after **200 & 400 mg** vs placebo (and 200 > 100); **100 mg ~ placebo**. No major latency effects. Trend pattern consistent with **S1 ↓ + S2 ↑** driving ratio. Effects independent of gender and habitual use (though high users had lower baseline S2)
[64]	18 healthy male military volunteers, 19–28	Double-blind, counterbalanced; 3 drug test days (plus familiarization); ≥48 h washout	**200 mg caffeine** vs **25 mg diphenhydramine** vs placebo; AEPs sampled during vigilance at ~10/40/70/100 min into task	**2-hour time-on-task fatigue** (vigilance) + drug effects	Continuous visual vigilance (primary) with simultaneous auditory clicks; long-latency auditory AEPs: **N1, P2, N2** (latency & amplitude)	Latencies (N1/P2) **increased over time** similarly across all drugs (time-on-task). Amplitudes: N1–P2 **declined over time** (no drug effect). Critical: **P2–N2 amplitude lower under caffeine vs placebo** (less processing of irrelevant auditory input; maintained focus on visual task). Diphenhydramine showed a **transient P2–N2 amplitude spike** in mid-cycle (~40–70 min), interpreted as attention drifting to auditory stimulus with sedation
[65]	31 healthy adults (27M/4F), 20–44	Between-groups; pre–post recordings;	**300 mg caffeine** beverage; AER re-recorded **~80 min**	None	Auditory click AER; **N1–P2 (vertex)** and **T-**	Placebo: **N1–P2 amplitude** ↓ (~79% baseline), latencies ~unchanged → habituation. Caffeine: **preserved**

	yrs, normal hearing	ethanol also at 1h & 4h	post (caffeine/placebo)		**complex (Ta/Tb; temporal)**; hemispheric latency asymmetries	**N1–P2 amplitude** (countered habituation), **Ta latency shortened** (~98% baseline), Ta–Tb amp ~unchanged; some ↑ in **ipsi–contra Ta latency difference** in subset (via shorter contralateral Ta). Ethanol: stronger ↑ in Ta asymmetry (mainly via **prolonged ipsilateral Ta**)
[66]	20 healthy male undergrads, 21–23 yrs, low habitual caffeine	Placebo-controlled **5-way randomized crossover** (each subject all treatments)	**160 mg caffeine** pre vs post testing each session	None	Oddball auditory ERP during attention testing; **N2–P300 complex** (plus VEP/MEP; SVRT/RVRT)	**N2–P300 amplitude** ↑ with caffeine (and with theanine); **combo greatest** (additive). **Latencies (P2/N2/P300)**: no meaningful treatment effects
[67]	37 analyzed (21M), 22–30 yrs; low habitual caffeine; sleep-deprived overnight	Double-blind, placebo-controlled, **counterbalanced crossover** (2 sessions)	**160 mg caffeine + 200 mg L-theanine** capsule; tested **50 min** post-dose	**Acute sleep deprivation** (overnight wakefulness)	Visual traffic-scene discrimination; ERP focus **P3b** (centro-parietal)	Active combo: **P3b latency** ↓ (~27–35 ms across sites) and **P3b amplitude** ↑ (~1.5–2.0 µV at several sites). Placebo: no reliable P3b changes
[68]	10 healthy male adults, mean ~27 yrs; habitual caffeine users (24h abstinence)	Randomized, double-blind, placebo-controlled **crossover** (3 trials)	**Caffeine mouth rinse** (20 s); no ingestion	None	**Stroop task**; ERP focus **P300**; plus sLORETA during rinse	**No significant P300 amplitude/latency changes** (caffeine vs maltodextrin vs placebo; pre vs post). Behavioral effects mostly null by standard stats
[69]	Healthy adult volunteers (mixed sex; mostly young adults); wide extraversion range	Multiple double-blind sessions; caffeine vs d-amphetamine vs placebo (within-subjects implied)	Caffeine given	Personality state: **extraversion** (trait moderator)	Warned reaction-time task; ERP focus **CNV (early vs late)**	**Early CNV amplitude** ↓ under caffeine (and d-amphetamine) vs placebo; **larger decreases in high-extraversion** individuals (drug × extraversion). Late CNV less drug-specific (more practice/order-related)
[70]	20 undergrads (5M/15F), 18–26 yrs; regular coffee users	Within-subjects, double-blind; 4 sessions separated ≥1 week	**1.0, 3.0, 7.5 mg/kg** High cognitive load via **dual-task difficulty** in decaf coffee	High cognitive load via **dual-task difficulty**	Visual dual-task; ERP focus mainly **P3** (plus P1/N1)	**P3 amplitude ↑ dose-dependently**, strongest over **parietal–occipital** sites; **P3 latency unchanged**. Early components largely unaffected (minor site-specific early effect at Fz)
[71]	44 healthy adults, 20–45 yrs; broad extraversion range	Double-blind **3-session crossover** (caffeine, chlordiazepoxide, placebo), ≥1 week apart	Caffeine given; CNV recorded **pre and post** within session	Trait: **extraversion–introversion**; strong **session habituation** (baseline CNV ↓ across sessions)	Reaction-time task; CNV components: **O wave**, **E wave**, average CNV at Cz	Drug effects strongest for **O wave** and depended on extraversion: caffeine tended to **increase O-wave** in higher extraversion and **decrease** in lower extraversion (opposite for chlordiazepoxide). Placebo ~no change
[72]	14 healthy right-handed male medical students, 20–27 yrs	Double-blind, placebo-controlled **randomized crossover** (3 sessions)	**200 mg caffeine**; repeated recordings across morning; **biphasic CNV-I** (early ~1–2h ↑, later ↓)	Trait moderator: **emotional lability vs stability**	Reaction-time S1–S2 task; ERP focus **CNV-I (early)**, CNV-II, RES	Caffeine: **CNV-I biphasic** then overall **suppression later**; diazepam: **CNV-I attenuation** vs placebo. Strong moderation: emotionally stable showed early caffeine enhancement; emotionally labile showed pronounced suppression under caffeine and diazepam
[73]	26 analyzed, ~18–31 yrs; regular coffee drinkers	Double-blind, counterbalanced **2-session crossover**, 1 week apart	**3 mg/kg caffeine** in decaf coffee (pre-task)	**Reward prospect cues** (motivational state)	Cued-reward **Stroop**; ERP focus **CNV (700–1100 ms post-cue)**, Ninc (400–500 ms), **LPC (700–800 ms)**	CNV: reward > no-reward, and **caffeine amplified reward-related CNV** (Reward × Caffeine interaction). Ninc robust but **not** modulated. LPC: reward-related increases, with **stronger reward effects under caffeine** (and overall larger LPC with caffeine)
[74]	47 healthy adults, mean ~26 yrs	Double-blind, placebo-controlled	Supplement drink with **55 mg caffeine** (+ other	**Time-on-task / mental fatigue across runs**	Visual oddball; ERP focus **P2 (200–260 ms)** and	Placebo: **run-related changes** decodable in **P2** and strong in **P3** (consistent with fatigue-related

		**between-groups**; ERPs over 3 runs	ingredients); runs **baseline, +30 min, +90 min**		**P3 (350–550 ms)**; MVPA decoding	evolution/increased effort). Supplement: **attenuated run-related differentiation**, especially reduced decoding differences in P2 and lower decoding accuracy in P3 windows → **more stable attentional processing over time**
[75]	18 right-handed undergrads (9M/9F), 18–31 yrs; caffeine users (12h abstinence)	Double-blind, placebo-controlled **within-subjects crossover**; 3 sessions	**3 mg/kg** (low) and **6 mg/kg** (high) in decaf coffee	Task state: **switch vs repeat**; **shift load** (single vs dual shift)	Cued task-switching; ERP focus cue-locked **P3** and **slow negativity (600–1000 ms)**; plus poststimulus ERPs	Key caffeine effect: **enhanced shift-related slow negativity** late in prep interval (esp. 900–1000 ms), **independent of shift load →** boosts general anticipatory control. Earlier cue-locked P2/N2 effects complex/nonspecific; poststimulus switch effects largely **not** caffeine-modulated
[76]	N=18 healthy undergrads (8M/10F), 18–30, regular coffee drinkers	Double-blind, placebo-controlled, **within-subjects**; 3 sessions counterbalanced; caffeine abstinence verified (saliva)	Placebo vs **3 mg/kg** vs **5 mg/kg** (in decaf coffee), per session (tested within session after ingestion)	None	Predictable task-switching (letter **color** vs **vowel/consonant**); ERP emphasis on **response–stimulus interval** anticipatory activity (early negativity 200–600 ms; late slow negativity 800–1200 ms), plus post-stimulus **P2/N2/P3**	**Key:** caffeine **amplified switch-specific late slow negativity** (more negative on switch trials; increased switch–repeat difference, mainly posterior/centro-parietal), suggesting stronger task-set updating preparation. Early negativity switch–repeat pattern **not significantly modulated** by caffeine. Post-stimulus: caffeine **shortened P2/P3 latencies**, **increased N2 amplitude** across conditions; **did not increase P2/P3 amplitudes** (no generalized perceptual/evaluation boost)
[77]	N=35 adults, 19–53 (mean reported ~22), predominantly female; habitual caffeine range 0–440 mg/day	Cross-sectional/individual-differences EEG study; habitual caffeine (mg/day) as continuous covariate	**Habitual intake**	None	**Stroop Colour and Word Test**; ERP focus on **N450** (325–465 ms at Cz) and **late occipital-parietal positivity** (600–900 ms at Pz/PO3/PO4)	**No reliable association** between habitual caffeine and Stroop ERP interference markers: N450 showed **no significant congruency effect** and **no congruency×caffeine** interaction. Late posterior positivity showed a **congruency effect**, but **no significant caffeine main effect** and **interaction only a trend** (p≈.06)
[78]	N=92 downhill MTB athletes (66M/26F), ~13–45 (eligibility ≥14) at 2024 Whistler Crankworx	Cross-sectional baseline field assessment at competition check-in; multivariable models for age/sex/caffeine	**Self-reported caffeine** in prior 24h (beverage count) and **deviation vs usual** (less/same/more)	None	Portable NeuroCatch auditory protocol (~375 s): oddball tones (evoke **N100, P300**) + semantic word pairs (evoke **N400**); midline electrodes (Fz/Cz/Pz)	Caffeine **presence vs absence** in prior 24h: **no meaningful ERP differences**. But **more-than-usual caffeine** was linked to **smaller N100 amplitude** and **faster N100 latency**; other components not clearly associated with caffeine deviation. (Also: females higher N100/P300 amplitudes; age associated with smaller N400 amplitude.)
[79]	N=40 healthy male undergrad medical students, 18–25	Within-subject pre–post (baseline vs post-caffeine)	**3 mg/kg** "pure caffeine" in water (with sugar/milk powder); ERPs measured **baseline and 40 min post-ingestion**	None	Auditory oddball (targets 20%): button press to target; ERP components **N1/P2/N2/P3** at Fz/Cz/Pz	**P3 amplitude increased significantly** at all sites (largest at Pz), indicating greater attentional/resource allocation. Latencies generally trended shorter; **P2 latency showed a significant overall effect**, while N1/N2/P3 latency decreases were **not significant**. Other component amplitudes not significantly changed
[80]	N=12 healthy right-handed adults, 19–34;	Double-blind, within-subjects; 4 sessions (≥72 h	**Caffeine 100 mg** (pill) in caffeine-alone and combo	None	Dichotic listening: attend one ear, respond to	Alcohol **prolonged MMN latency** (slower deviance detection) without amplitude change.

	light–moderate caffeine/alcohol users	apart): placebo, caffeine, alcohol, caffeine+alcohol	sessions; alcohol **0.55 g/kg** beverage; ERPs recorded after dosing; BAC ~0.06–0.07% during alcohol conditions)		deviants in attended stream; ERP focus **MMN** (change detection), **processing negativity** (PNe/PNl), plus **N2b** and **P3b**	**Caffeine+alcohol reversed MMN latency delay** toward placebo; caffeine alone had **no MMN effect**. Alcohol reduced **PNe amplitude** (early selective attention) and increased **P3b latency**; caffeine **did not** reverse those alcohol effects
[81]	N=16 healthy female university students (mean age ~24), moderate caffeine/alcohol consumers	Double-blind, within-subjects; 4 sessions: placebo, alcohol, caffeine, alcohol+caffeine	**Caffeine 200 mg**; alcohol **0.7 ml/kg** (as administered in study); ERPs during tasks	None	Simple RT + choice RT; ERP focus **P200, N200, P300**, and **N500 difference wave** (working memory–related, 220–900 ms area)	Caffeine: **shortened N200 latency** (esp. in choice RT; right sites) and **increased P300 amplitude only in choice RT**; no clear P200 effects; no P300 latency reduction. N500: caffeine alone **no major effect**, but **caffeine+alcohol reduced N500 area** (front/left/centro-parietal sites), suggesting reduced sustained WM engagement under the combination. Alcohol altered N500 scalp distribution and slowed decision time behaviorally
[82]	N=10 healthy young men (mean age ~22), very low habitual caffeine	Randomized, double-blind, placebo-controlled crossover; 3 sessions (≥2 days apart)	**Caffeine nasal spray**, **glucose spray**, or placebo; Stroop **pre vs post** 20 s spray	None	Stroop task with continuous EEG; ERP focus **P300 amplitude/latency** (plus ongoing oscillations during spray)	**Caffeine nasal spray: no significant P300 amplitude/latency effects** (only nonsignificant trends). By contrast, glucose spray increased P300 amplitude on incongruent trials and altered/shortened P300 metrics for low-demand stimuli (included here only as context)
[83]	3 groups, each n=12: **GAD**, **panic disorder**, **healthy controls**; adults 18–65; habitual caffeine users	Double-blind, placebo-controlled, **triple-crossover** caffeine challenge; extensive 5-hour monitoring	Placebo vs **250 mg** vs **500 mg** caffeine; effects peaked **~1 hour** post-dose	**None**	Broad EEG + **auditory evoked potentials/ERPs** (components including **N1/P2/N2** measures); multiple physiological and subjective anxiety outcomes	Dose-related arousal/ERP changes overall (shorter latencies, amplitude changes). **GAD showed exaggerated ERP reactivity** vs controls, notably greater **reduction in N1–P2 amplitude** (heightened sensitivity in early auditory processing), plus altered alpha activity. **Panic disorder** showed more selective changes (e.g., **N2 latency** and **N2–P2 amplitude**), generally smaller than GAD
